# Ultrasound-Assisted Synthesis of Piperidinyl-Quinoline Acylhydrazones as New Anti-Alzheimer’s Agents: Assessment of Cholinesterase Inhibitory Profile, Molecular Docking Analysis, and Drug-like Properties

**DOI:** 10.3390/molecules28052131

**Published:** 2023-02-24

**Authors:** Rubina Munir, Sumera Zaib, Muhammad Zia-ur-Rehman, Nadia Hussain, Faryal Chaudhry, Muhammad Tayyab Younas, Fatima Tuz Zahra, Zainab Tajammul, Noman Javid, Ayed A. Dera, Hanan A. Ogaly, Imtiaz Khan

**Affiliations:** 1 School of Chemistry, University of the Punjab, Lahore 54590, Pakistan; 2 Department of Chemistry, Kinnaird College for Women, Lahore 54000, Pakistan; 3 Department of Basic and Applied Chemistry, Faculty of Science and Technology, University of Central Punjab, Lahore 54590, Pakistan; 4 Applied Chemistry Research Centre, PCSIR Laboratories Complex, Lahore 54600, Pakistan; 5 Department of Pharmaceutical Sciences, College of Pharmacy, Al Ain University, Al Ain P.O. Box 64141, United Arab Emirates; 6 AAU Health and Biomedical Research Center, Al Ain University, Abu Dhabi P.O. Box 144534, United Arab Emirates; 7 Department of Chemistry, Quaid-i-Azam University, Islamabad 45320, Pakistan; 8 Chemistry Department (C-Block), Forman Christian College, Ferozepur Road, Lahore 54600, Pakistan; 9 Department of Clinical Laboratory Sciences, College of Applied Medical Sciences, King Khalid University, Abha 62529, Saudi Arabia; 10 Chemistry Department, College of Science, King Khalid University, Abha 61421, Saudi Arabia; 11 Biochemistry and Molecular Biology Department, Faculty of Veterinary Medicine, Cairo University, Giza 12211, Egypt; 12 Department of Chemistry and Manchester Institute of Biotechnology, The University of Manchester, 131 Princess Street, Manchester M1 7DN, UK

**Keywords:** quinoline, *N*-acylhydrazone, piperidine, Alzheimer’s disease, neurological disorder, cholinesterases, molecular docking, physicochemical properties

## Abstract

Alzheimer’s disease (AD) is one of the progressive neurological disorders and the main cause of dementia all over the world. The multifactorial nature of Alzheimer’s disease is a reason for the lack of effective drugs as well as a basis for the development of new structural leads. In addition, the appalling side effects such as nausea, vomiting, loss of appetite, muscle cramps, and headaches associated with the marketed treatment modalities and many failed clinical trials significantly limit the use of drugs and alarm for a detailed understanding of disease heterogeneity and the development of preventive and multifaceted remedial approach desperately. With this motivation, we herein report a diverse series of piperidinyl-quinoline acylhydrazone therapeutics as selective as well as potent inhibitors of cholinesterase enzymes. Ultrasound-assisted conjugation of 6/8-methyl-2-(piperidin-1-yl)quinoline-3-carbaldehydes (**4a**,**b**) and (un)substituted aromatic acid hydrazides (**7a-m**) provided facile access to target compounds (**8a-m** and **9a-j**) in 4–6 min in excellent yields. The structures were fully established using spectroscopic techniques such as FTIR, ^1^H- and ^13^C NMR, and purity was estimated using elemental analysis. The synthesized compounds were investigated for their cholinesterase inhibitory potential. In vitro enzymatic studies revealed potent and selective inhibitors of AChE and BuChE. Compound **8c** showed remarkable results and emerged as a lead candidate for the inhibition of AChE with an IC_50_ value of 5.3 ± 0.51 µM. The inhibitory strength of the optimal compound was 3-fold higher compared to neostigmine (IC_50_ = 16.3 ± 1.12 µM). Compound **8g** exhibited the highest potency and inhibited the BuChE selectively with an IC_50_ value of 1.31 ± 0.05 µM. Several compounds, such as **8a-c**, also displayed dual inhibitory strength, and acquired data were superior to the standard drugs. In vitro results were further supported by molecular docking analysis, where potent compounds revealed various important interactions with the key amino acid residues in the active site of both enzymes. Molecular dynamics simulation data, as well as physicochemical properties of the lead compounds, supported the identified class of hybrid compounds as a promising avenue for the discovery and development of new molecules for multifactorial diseases, such as Alzheimer’s disease (AD).

## 1. Introduction

In the current healthcare system, dementia has proven as a devastating health issue and a major societal challenge around the globe and continues to impact the global population with time. Alzheimer’s disease (AD) is the paramount cause of vascular dementia, a prevalent, multifactorial, and neurological disorder identified at molecular level by proteopathies, mitochondrial aberrations, oxidative stress, and neuroinflammation [1,2]. The expected but alarming rise in number of patients affected by dementia with AD from 55 million to 139 million by 2050, 60% of them belonging to low- and middle-income countries, underscores the severity and continuous failure to produce anti-Alzheimer’s drugs [3].

Alzheimer’s disease (AD) is a severe and irreversible progressive neurodegenerative disorder with a complex pathophysiology and clinical symptoms that causes cognitive impairment, decreased thinking ability, and motor and executive functions. The distinctive brain changes associated with AD result from the decline of inter-neuronal connectivity and neuronic apoptosis, particularly in areas such as the limbic system [4]. Though the pathophysiology and etiology of AD remain ambiguous, cholinergic dysfunction has been predicted to play a vital role in the progress of AD [5]. The important parts of the brain, such as the hippocampus and cerebral cortex, become deficient in the neurotransmitter acetylcholine (ACh), which is closely linked with AD [6]. Currently, the strategies leading to enhanced levels of cholinergic neurotransmission with reduced hydrolysis of ACh are still considered the most effective treatment for AD. The hydrolysis of ACh is mediated by two well-known cholinesterases: acetylcholinesterase (AChE) and butyrylcholinesterase (BuChE) [7]. AChE is more vigorous than BuChE by 10^13^-fold up and is responsible for nearly 80% of ACh hydrolysis [8]. There are two binding sites in the enzymatic pocket of AChE; the catalytic active site (CAS) and the peripheral anionic site (PAS), which acts as an allosteric modulation site of enzymatic activity [9,10]. The involvement of AChE, through the PAS, in the aggregation of the Aβ peptide has given a new meaning to the therapeutic use of mixed or non-competitive acetylcholinesterase inhibitors (AChEIs), as they could act both by restoring the acetylcholine levels and interfering on the aggregation of the Aβ peptide [11,12]. BuChE, whose expression and activity increase in advanced AD states, is also responsible for the hydrolysis of acetylcholine and may have a supporting role when AChE activity is reduced [13,14].

The current treatment approaches give only symptomatic remedies to AD patients. The marketed drugs such as tacrine, donepezil, rivastigmine, and galantamine prove ineffective at halting or reversing the progression of AD [15,16,17,18], although they vary in structural features and pharmacokinetic profiles [17]. These drugs are hampered by their after-effects, including gastric disturbance, liver toxicity, and hypotension [19,20,21,22,23]. Among the FDA-approved drugs, only aducanumab is used as an etiological treatment, but poor efficacy in the phase III trials makes this drug controversial [18,24]. Therefore, the development of new and more effective drugs to treat multifaceted diseases remains a promising field of research.

**Figure 1:**
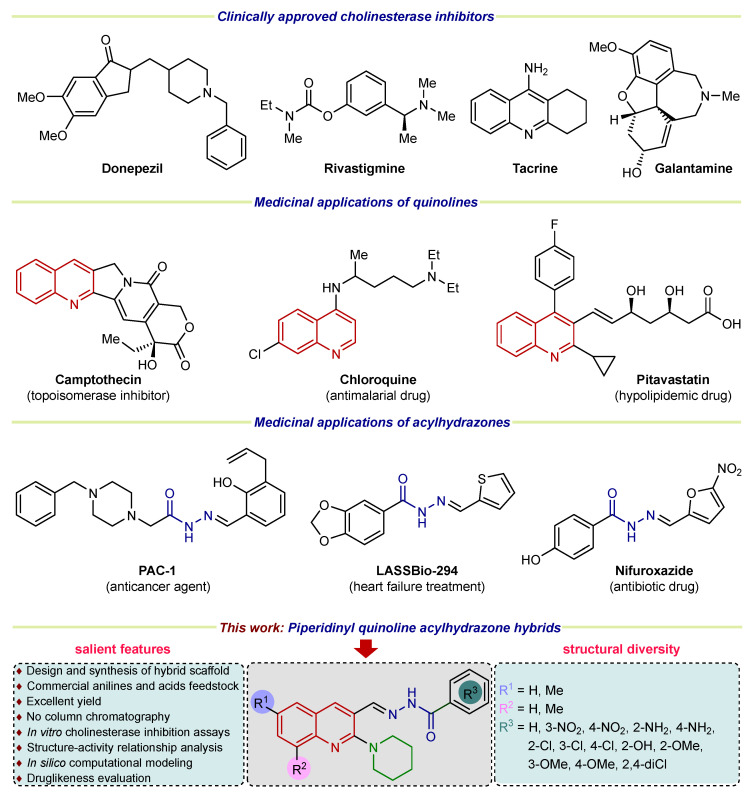
Examples of clinically approved drugs for AD and rationale of current study.

On the other hand, *N*-acyl hydrazones represent a class of organic compounds recognized for their various pharmacological applications [25]. Acyl hydrazone pharmacophore is widely distributed in numerous natural products, agrochemicals, and bioactive drug candidates [26]. The important medicinal applications including antiplatelet [27], antiproliferative [28], antitrypanosomal [29], antimicrobial [30], anti-inflammatory, anti-analgesic [31], antitumor [32], antioxidant, antihypertensive [33], inhibitory potential of alkaline phosphatase [34], and treatment of neurological disorders [26] have been reported. In parallel, the literature reports evidenced a well-established medicinal chemistry profile of nitrogen-containing heterocycles (quinoline and piperidine) [35,36,37,38,39,40]. Therefore, combining three biologically active pharmacophores into a single unit giving rise to a hybrid framework, could produce beneficial effects in treating neurodegenerative disorders such as Alzheimer’s disease. Figure 1 demonstrates the representative examples of commercial cholinesterase inhibitors and hybridization strategies leading to the piperidinyl-quinoline substituted *N*-acylhydrazone scaffold investigated in this study.

Building on our previous track record in the exploration of anti-Alzheimer’s compounds [41,42,43,44] and the current lack of treatment modalities, possibly due to a large gap between basic research and translation to novel therapeutics and a very little success at the late-stage clinical trials inspire the drug discovery and medicinal chemists to explore the wider chemical space, thus delivering new and promising structural leads with potent anti-Alzheimer’s potential. Therefore, we herein established a hybrid scaffold incorporating quinoline, piperidine, and acyl hydrazone fragments as a favorable candidate for the generation of a distinct library of cholinesterase inhibitors. The concept of hybridization was used for the design of novel cholinesterase inhibitors, which is based on the assembly of two or more bioactive pharmacophores into a single unit. In vitro cholinesterase, inhibitory assays were performed against acetylcholinesterase and butyrylcholinesterase enzymes, and the acquired potency was strengthened using computational modeling analysis. The investigation of druglikeness properties also revealed favorable data and a safer profile of the synthesized hybrid compounds.

## 2. Results and Discussion

### 2.1. Synthetic Chemistry

**Scheme 1:**
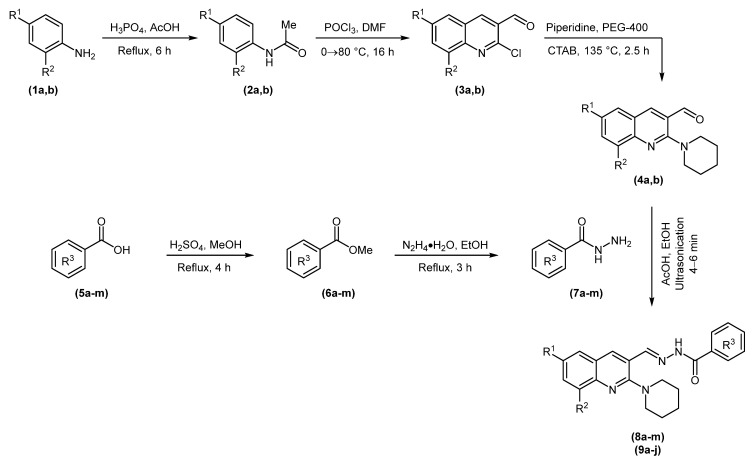
Synthetic route to piperidinyl-quinoline *N*-acylhydrazones (**8a-m** and **9a-j**).

The target compounds were synthesized following the synthetic route illustrated in Scheme 1. *N*-Acetylation of *o*- and *p*-toluidine **1** followed by Vilsmeier–Haack formylation of **2** yielded 2-chloro-3-formylquinolines (**3a** and **3b**). Nucleophilic aromatic substitution of **3a** or **3b** with piperidine produced 2-(piperidin-1-yl)quinoline-3-carbaldehydes (**4a** and **4b**) [41,43,45]. In parallel, Fischer esterification of substituted benzoic acids (**5a-m**) gave their corresponding methyl esters (**6a-m**), which were treated with hydrazine monohydrate to obtain acid hydrazides (**7a-m**) [46,47]. The acid-catalyzed condensation of synthesized hydrazides (**7a-m**) and aldehydes (**4a** and **4b**) delivered two sets of piperidinyl-quinoline *N*-acylhydrazones (**8a-m** and **9a-j**) in 15–45 min in good yields. Another set of experiments was performed in an ultrasonic bath that reduced the reaction time to 4–6 min with slightly improved yields. The comparative results are presented in Table 1.

### 2.2. In Vitro Cholinesterase Inhibition and Structure–Activity Relationship Analyses

**Figure 2:**
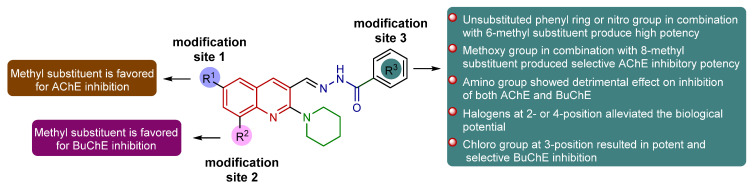
Schematic representation of structural modifications and their effect on inhibition profile.

The newly designed and synthesized piperidinyl-quinoline acylhydrazone derivatives (**8a-m** and **9a-j**) with diverse substitution patterns were evaluated for their in vitro inhibitory efficacy against cholinesterases (AChE and BuChE) using Ellman’s method [48,49]. In vitro inhibition results of tested compounds are presented in Table 2 and Table 3. The commercially available compounds neostigmine and donepezil were used as positive controls for AChE and BuChE, respectively. Various potencies of different orders of magnitude were achieved towards both targets. The designed scaffold comprised three modification sites (R^1^, R^2^, R^3^) for structural variations and three rigid motifs, including quinoline, piperidine, and acylhydrazone moieties. In particular, the benzohydrazide region served as a central motif in the formation of key interactions such as hydrogen bonding. Nitrogen atom of the hydrazone nucleus was found to establish hydrogen bonding and acts as a hydrogen bond donor, whereas carbonyl oxygen also interacted through H-bonding contacts. The introduction of a methyl group at the 6- or 8-position of the quinoline ring established alkyl linkages with various amino acid residues. The piperidine ring was involved in the formation of π-sigma bonds and π-alkyl linkages, whereas the quinoline core displayed π–π stacked interactions. Consequently, all the bioactive pharmacophoric regions and structurally stretchy sites are crucial for inhibitory potency due to their involvement in the formation of multiple significant interactions with several amino acid residues within the active pocket of both enzymes. The introduction of a substitution pattern in the form of electron-donating group (EDG) or electron-withdrawing group (EWG) not only provided the desired structural diversity but also contributed pharmacokinetically to attaining drug-like properties. As the in vitro cholinesterase inhibitory profiles presented herein depict a diverse picture, the structure–activity relationship analyses against both targets (AChE/BuChE) are worth investigating to propel the drug discovery endeavor to new avenues. Figure 2 illustrates a graphical representation of key pharmacophores alongside structural modification sites and crucial outcomes.

**Figure 3:**
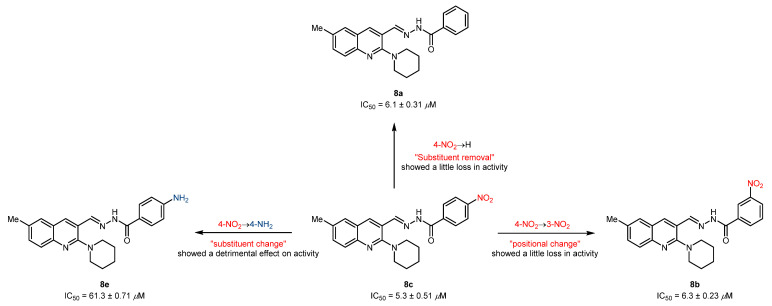
Illustration of positional and substituent effect on the inhibitory efficacy against AChE.

The striking feature of the present study is the exceptional role of various substituents/functional groups towards the inhibitory efficiency of cholinesterases, therefore leading to a distinct degree of biological inhibition potential. Keeping this in mind, we have divided the tested derivatives into two sets of compounds (**8a-m** and **9a-j**) primarily based on the substitution pattern (R^1^ and R^2^) at the quinoline ring. In the first set of compounds, **8a-c** were identified as potent and dual inhibitors of AChE and BuChE enzymes; however, **8c** showed better results and remained as a lead candidate for the inhibition of AChE with an IC_50_ value of 5.3 ± 0.51 µM. This inhibitory strength was 3-fold higher compared to neostigmine (IC_50_ = 16.3 ± 1.12 µM). A slight loss in activity was noticed when the nitro group at the 3-position of the phenyl ring in compound **8c** was moved to the 4-position (**8b**) or completely removed (**8a**). Both compounds exhibited similar inhibitory strength with IC_50_ values of 6.3 ± 0.23 and 6.1 ± 0.31 µM, respectively. Although the biological activities were slightly diminished, the positional change or substitution removal imparted beneficial effects, thus supporting the generation of compound libraries. However, a drastic effect on inhibition efficacy was realized when a highly polarizable electron-deficient (NO_2_) group was replaced with an electron-rich (NH_2_) group. This effect could possibly be attributed to the removal of a hydrogen bond acceptor and incorporation of a hydrogen bond donor, thus alleviating the explicit interaction formed by the former group (Figure 3). The remaining compounds in the first set showed <50% inhibitory efficacy (31–43%) against AChE.

**Figure 4:**
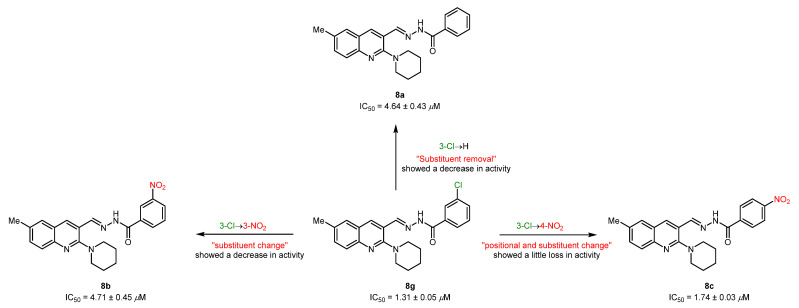
Illustration of positional and substituent effect on the inhibitory efficacy against BuChE.

In parallel, the synthesized piperidinyl-quinoline acylhydrazones (**8a-m**) were also tested against the butyrylcholinesterase enzyme. A similar inhibition profile was obtained, revealing compound **8g** as the lead and selective candidate with an IC_50_ value of 1.31 ± 0.05 µM, a 5.5-fold stronger inhibition than donepezil (IC_50_ = 7.23 ± 0.12 µM). This derivative incorporates a chloro substituent at the 3-position of benzohydrazide, which fits well in the active pocket of BuChE. The exchange of an inductively electron-withdrawing group (Cl) with a stronger electron-withdrawing (NO_2_) group demonstrated a significant influence on the activity strength, thus reducing the inhibition ability to 3-folds (**8b**; IC_50_ = 4.71 ± 0.45 µM). The deletion of the chloro group also produced similar results to the substituent swapping approach (**8a**; IC_50_ = 4.64 ± 0.43 µM). However, the loss in inhibitory strength was compensated with the introduction of the nitro group at the 4-position of benzohydrazide (**8c**; IC_50_ = 1.74 ± 0.03 µM). This change contributes to the rehabilitation and retention of biological potency (Figure 4). Although these hybrid structures show a common trend with a decline in inhibitory potency in comparison to the lead structure **8g**, they still represent potent inhibitors compared to standard drug (donepezil). The remaining compounds exhibited <50% inhibition in the range of 13–39%.

**Figure 5:**
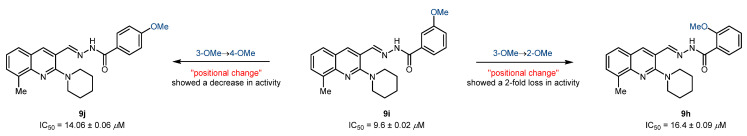
Illustration of positional effect on the inhibitory efficacy against AChE.

The second set of compounds (**9a-j**) bearing a methyl group at 8-position of the quinoline core showed a completely opposite trend for the inhibition of cholinesterases. The inhibitory potential of acylhydrazones against cholinesterase enzymes is reported in Table 3. Compounds **9h-j** bearing an electron-donating (OMe) group at 2-, 3-, and 4-position of benzohydrazide moiety were found as the only potent but completely selective inhibitors with IC_50_ values in the range of 9.6–16.4 µM. Compound **9i** bearing a 3-OMe substituent on benzohydrazide moiety displayed the highest inhibition with an IC_50_ value of 9.6 ± 0.02 µM. Subsequently, the effect of the positional change was investigated, and results revealed that the movement of the methoxy group to 2-, or 4-position showed a decline in inhibitory efficacy with IC_50_ values of 16.4 ± 0.09 (**9h**) and 14.06 ± 0.06 µM (**9j**), respectively (Figure 5). The remaining compounds of set 2 were poor inhibitors and showed <50% inhibition (27–41%). In comparison to the first set of compounds, **9a-j** failed to produce any potent lead against BuChE, and mild inhibition potency (27–40%) was observed. These results clearly suggested that the tested compounds were not reasonably accommodated in the active site; therefore, no beneficial interactions and inhibition potential were noted.

To summarize, the first round of in vitro evaluation of AChE and BuChE inhibitory potency and diverse structure–activity relationships in both sets of compounds confirmed our design strategy and provided explicit guidance for further optimization of piperidinyl-quinoline acylhydrazones into potential drug candidates for the treatment of Alzheimer’s disease.

### 2.3. Molecular Docking Studies

To justify the in vitro biological results, the most potent and selective compounds were docked within the active site of AChE and BuChE enzymes. In vitro results elucidated that several compounds show potent inhibitory efficacy against both enzymes, as reported previously [50]. Compound **9i** showed complete selectivity towards AChE with two-fold inhibition, while **8g** inhibited the BuChE enzyme exclusively. In parallel, **8a–c** were significantly active and identified as dual inhibitors of AChE and BuChE. Therefore, both selective and potent inhibitors were docked within the active pocket of both cholinesterases. For the purpose of exploring the binding interactions of potent compounds, crystallographic structures were downloaded from the protein databank. For docking analysis, the crystallographic structure of human acetylcholinesterase (4BDT) [41,43] and butyrylcholinesterase (4BDS) [41,43] were selected.

**Figure 6:**
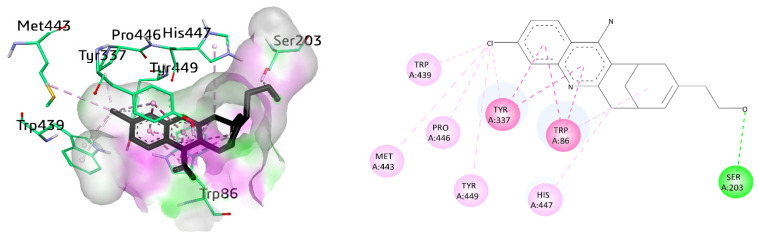
Three-dimensional and two-dimensional visualization of **huprine W**, π–π stacking interactions exhibited as fuchsia, hydrogen bond as green, and alkyl interactions as light pink dashed lines.

Various amino acid residues such as Leu76, Tyr124, Phe338, Gly122, Trp286, Tyr337, Val 340, Phe297, Leu289, Tyr72, Ser298, Ser125, Arg 296, Ser203, Tyr341, Ala204, and His447 surrounded the active pocket of AChE. The active compounds **8a**, **9i**, and cognate ligand **huprine W** exhibited hydrogen bonding and π–π interactions. The cognate ligand displayed conventional hydrogen bonding with Ser203 and stacked with two π–π stacking interactions against Trp86 and Tyr337. Additionally, π–alkyl interactions were also observed with Trp86, and multiple alkyl linkages with Trp439, Pro446, Tyr449, Met443, His447, and Tyr337 were also noticed (Figure 6).

**Figure 7:**
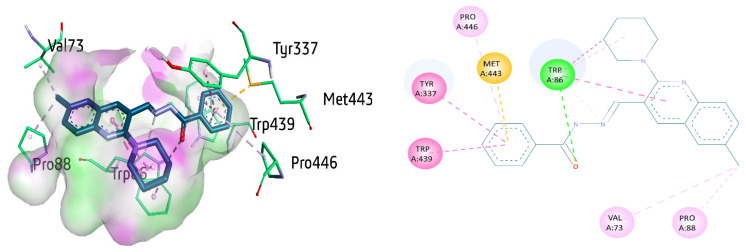
Three-dimensional and two-dimensional visualization of compound **8a** against AChE. π–π stacking interactions are shown as fuchsia, hydrogen bond as green, π-sulfur as yellow, π-sigma as purple, π-alkyl and alkyl interactions as light pink dotted lines.

The active compound **8a** showed multiple interactions with amino acid residues within the active site of AChE. These interactions include π–π stacking with Tyr337, Trp439, π-alkyl interaction with Pro446, and π-sulfur interaction with Met443 and benzohydrazide ring. Conventional hydrogen bonding was unveiled between the carbonyl oxygen atom and Trp86 (2.54 Å), the most significant residue present in the active site of AChE. Trp86 also depicted π–π stacked interaction (4.92 Å) against the 6-methyl quinoline ring of **8a** as well as the π–sigma bond and π-alkyl linkage with piperidine ring. Trp86 also showed a carbon hydrogen bond with benzohydrazide. Moreover, Val73 and Pro88 exhibited alkyl linkage with methyl quinoline ring (Figure 7).

**Figure 8:**
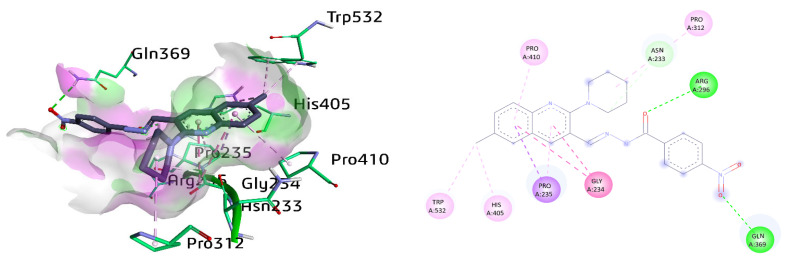
Three-dimensional and two-dimensional visualization of compound **8c** against AChE. Amide-π stacking interactions are shown as dark fuchsia, hydrogen bond as green, C-H bond as light green, π-alkyl and alkyl interactions as light pink, and π-sigma as dark purple dotted lines.

The most potent compound **8c** against AChE exhibited different vital interactions with the amino acid residues of the active pocket of protein receptors. These include amide-π stacked against Gly234 and conventional hydrogen bonding between Arg296 and carbonyl oxygen atom as well as Gln369 and nitro group, respectively. Moreover, Pro235 also revealed π-sigma and π-alkyl interaction with the methyl quinoline ring with a distance of 3.92 Å and 4.07 Å, respectively. Subsequently, alkyl linkages were observed with amino acids Trp532, His405, and Pro312. Moreover, Asn233 also exhibited a carbon-hydrogen bond with a distance of 3.20 Å with the piperidine ring of compound **8c**. π-Alkyl linkage was also realized between Pro410 and the quinoline ring, as shown in Figure 8.

**Figure 9:**
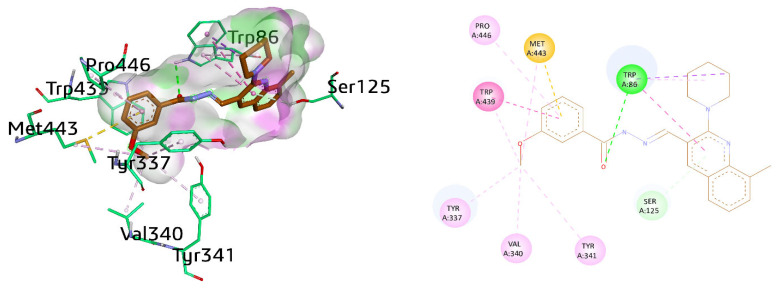
Three-dimensional and two-dimensional visualization of compound **9i** against AChE. π–π stacking interactions are shown as fuchsia, hydrogen bond as green, π-sulfur as yellow, π-alkyl and alkyl interactions as light pink, and C-H bond dotted lines.

Similarly, another selective and active inhibitor **9i** docked within the AChE pocket revealed multiple important interactions. Trp439 showed π–π stacked interactions against benzohydrazide ring of **9i** and an alkyl linkage with a methyl substituent. Furthermore, Met443 and Pro446 showed π-sulfur and π-alkyl interactions, respectively, with the benzohydrazide ring. Trp86 exhibited conventional hydrogen bonding with a carbonyl oxygen atom in addition to π–π stacked interaction (5.26 Å) with an 8-methyl quinoline ring. Trp86 also showed π-sigma and π-alkyl linkage with the piperidine ring. Several alkyl bonds involving Tyr337 (4.28 Å), Val340, Tyr341, and Met443 were also present with the methoxy group present at benzohydrazide moiety. π-Donor hydrogen bond was also noticed between Ser125 and the quinoline ring, as shown in Figure 9.

**Figure 10:**
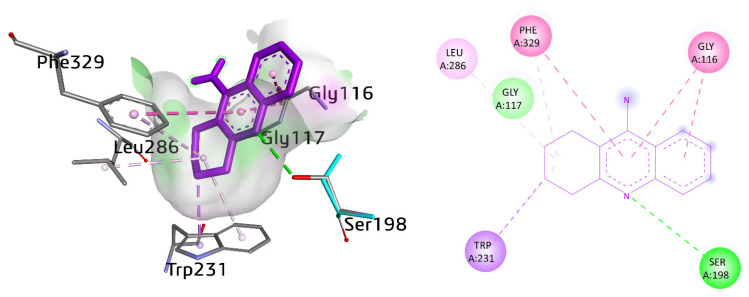
Three-dimensional and two-dimensional visualization of **tacrine**. π–π T shaped interactions are shown as light fuchsia, amide-π stacked as dark fuchsia, hydrogen bond as green, alkyl interactions as light pink, C-H bond as light green, and π-sigma as purple dotted lines.

On the other hand, the active pocket of butyrylcholinesterase encompassed several amino acid residues such as Trp82, His438, Tyr332, Leu286, Val288, Phe398, Trp231, Gly116, Gly117, Ala328, Pro285, and Phe329. Several interactions were observed by selective as well as potent compounds with BuChE. The cognate ligand tacrine exhibited various interactions. For instance, π–π T-stacked interaction with Phe329 with a distance of 5.62 Å. Gly116 also showed amide-π stacked interactions (4.13, 4.37 Å). Conventional hydrogen bonding was also noticed between the nitrogen atom of the ring and the hydrogen atom of Ser198, with a distance of 2.80 Å. Additionally, Trp231 exhibited π-sigma and π-alkyl linkage with a distance of 3.57 and 4.63 Å, respectively. A π-linkage was also observed with Phe329 (5.48 Å) (Figure 10).

**Figure 11:**
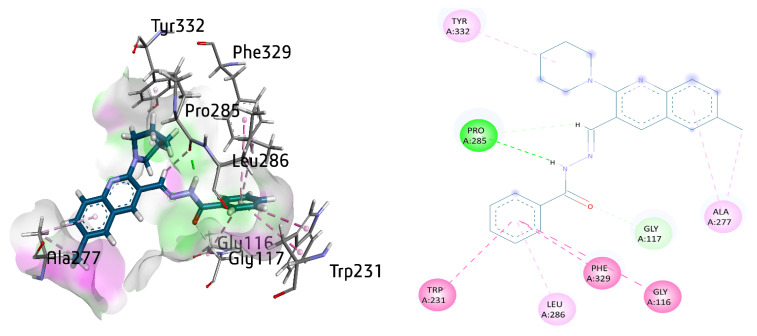
Three-dimensional and two-dimensional visualization of compound **8a** against BuChE. π–π stacking interactions are shown as light fuchsia, amide-π interaction as dark fuchsia, hydrogen bond as green, π-alkyl and alkyl interactions as light pink, and C-H bond as light green dotted lines.

The active inhibitor **8a** against BuChE exhibited several important interactions with the amino acid residues of the active site. Among them, π–π T-shaped interactions between Phe329 and Trp231, as well as amide-π stacked interaction between Gly116 and benzohydrazide moiety, were the most prominent. A π-alkyl linkage between the Leu286 aromatic ring was also observed, whereas conventional hydrogen bonding and carbon hydrogen bond were also noticed with amino acid residue Pro285 with distance of 1.93 and 3.08 Å, respectively. Gly117 also exhibited a hydrogen bond with carbonyl oxygen atom. Additionally, Ala277 showed π-alkyl and an alkyl linkage with the methyl quinoline ring of **8a**. Finally, a π-alkyl linkage was also noticed between the piperidine ring and Tyr332 with a distance of 4.07 Å, as shown in Figure 11.

**Figure 12:**
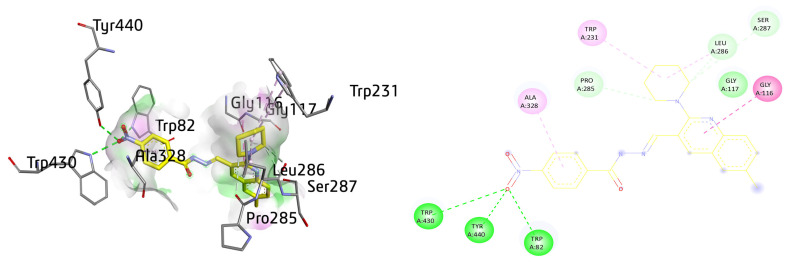
Three-dimensional and two-dimensional visualization of compound **8c** against BuChE. Amide-π stacking interactions are shown as dark fuchsia, hydrogen bond as green, π-alkyl and alkyl interactions as light pink, and C-H bond as light green dotted lines.

Compound **8c** depicted multiple important interactions such as amide-π stacked, alkyl and π-alkyl, conventional hydrogen bond, C-H bond, and van der Waals interactions within the active pocket of BuChE. The oxygen of the -NO_2_ group present at benzene is making multiple hydrogen bonds with the amino acid residues such as TRP430, TYR440, and TRP82. The quinoline ring of compound **8c** is amide-π stacked against GLY116, whereas van der Waals interactions were noticed with GLY117. The piperidine ring also revealed alkyl interactions with TRP231 (6.90 Å) and LEU286 (4.66 Å) and multiple C-H bonds with PRO285, SER287, and LEU286. The aromatic ring of the compound also exhibited π-alkyl linkage with the ALA328 with a distance of 4.36 Å, as shown in Figure 12.

**Figure 13:**
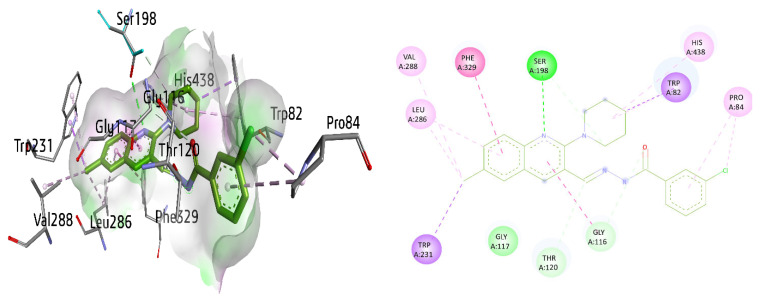
Three-dimensional and two-dimensional visualization of compound **8g** against BuChE. π–π stacking interactions are shown as light fuchsia, hydrogen bond as green, π-sigma as purple, π-alkyl and alkyl interactions as light pink, and C-H bond as light green dotted lines.

Finally, the potent and selective lead **8g** against BuChE exhibited multiple interactions, including π–π T-shaped interactions with Phe329 and amide-π stacked interaction with Gly116 by the quinoline ring, π-sigma interaction with Trp231, conventional hydrogen bonding with Ser198, alkyl and π-alkyl linkage with Leu286 and alkyl bond with Val288 in the active pocket of the enzyme. Furthermore, the piperidine ring revealed a π-sigma linkage with Trp82, a carbon hydrogen bond with Ser198 as well as a π-alkyl linkage with His438. Pro84 exhibited an alkyl and a π-alkyl linkage with a 3-chlorophenyl ring. Gly116 and Thr120 also interacted through carbon hydrogen bonds, as shown in Figure 13.

In summary, all the potent and selective compounds, as well as co-crystallized ligands, showed various crucial interactions against acetylcholinesterase and butyrylcholinesterase enzymes. Some amino acid residues do not reveal the binding at a similar position but interact through other residues and are moderately aside from the active site.

Various types of interactions between compounds (**8a**, **8c**, and **9i**) and receptor (PDB; 4BDT), and compound (**8a**, **8c**, and **8g**) and receptor (PDB; 4BDS) with appropriate distances and atoms involved in the binding interactions are demonstrated in Figure 6, Figure 7, Figure 8, Figure 9, Figure 10, Figure 11, Figure 12 and Figure 13 and Tables S1 and S2.

### 2.4. SeeSAR Visual Drug Design

**Figure 14:**
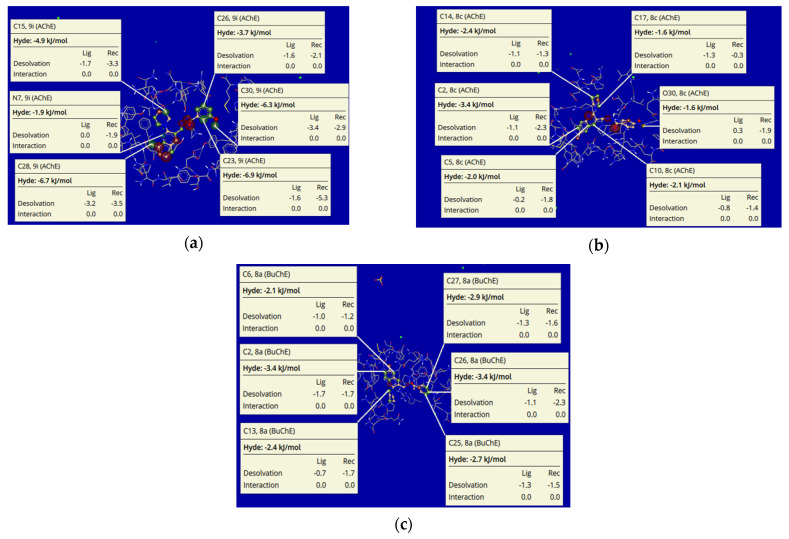
Visualization and investigational modes of the docked pose of compounds **9i** (**a**) **8c** (**b**), and **8a** (**c**) within the active pocket of AChE (pdb: 4BDT) and BuChE (pdb: 4BDS), respectively. The green color depicts the favorable and contributing atoms revealing interactions within the active site; subsequently, light green are chlorine atoms, red color are oxygen atoms, and blue are nitrogen atoms.

Among all the tested compounds, **8c** and **9i** showed exceptional Hyde energies against AChE, while **8a** was identified with a similar profile against BuChE. The visual and explore modes of the docked poses of inhibitors **9i** (a), **8c** (b), and **8a** (c) depicted expressible, inventive, and important conformations using the SeeSAR tool of LeadIT software [51,52]. Figure 14 shows the iterative and interactive optimization of leads exhibiting the binding and non-binding capacity of inhibitors. Different atoms exhibited Hyde energies in **9i** (AChE), such as C15 (−4.9 kj/mol), C26 (−3.7 kj/mol), C30 (−6.3 kj/mol), C28 (−6.7 kj/mol), C23 (−6.9 kj/mol) and N (−1.9 kj/mol). In parallel, compound **8c** also showed Hyde energies such as C14 (−2.4 kj/mol), C17 (−1.6 kj/mol), C2 (−3.4 kj/mol), C5 (−2.0 kj/mol), C10 (−2.1 kj/mol) and O30 (−1.6 kj/mol). Likewise, compound **8a** (BuChE) also depicted Hyde energies such as C6 (−2.1 kj/mol), C2 (−3.4 kj/mol), C13 (−2.4 kj/mol), C27 (−2.9 kj/mol), C26 (−3.4 kj/mol) and C25 (−2.7 kj/mol) as shown in Figure 14. Desolvation and interactions for compounds **9i**, **8c**, and **8a** are also shown in Figure 14. The approach predicts the visual and interpretable feedback for implicit hydrogen bonds and dehydration and confirms our molecular docking results obtained using FlexX default parameters.

### 2.5. Molecular Dynamics Simulation

**Figure 15:**
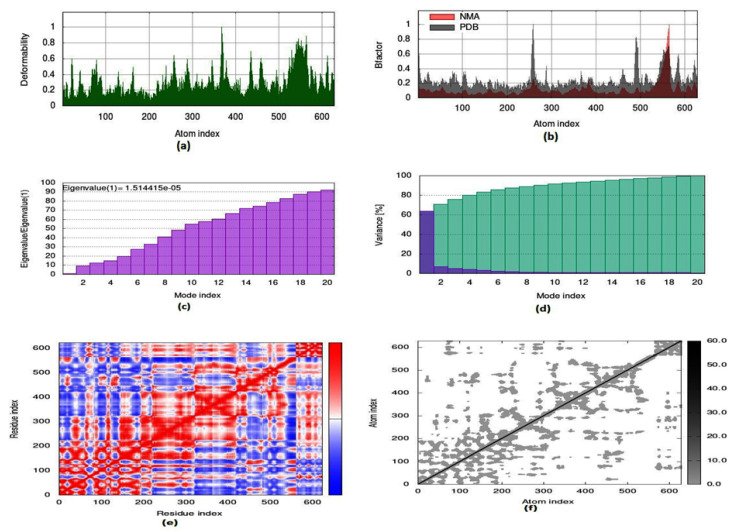
Molecular dynamics simulation of **8c** complex (AChE) by iMODS server. (**a**) Deformability, (**b**) B-factor values, (**c**) eigenvalue, (**d**) variance, (**e**) covariance, and (**f**) elastic network model.

**Figure 16:**
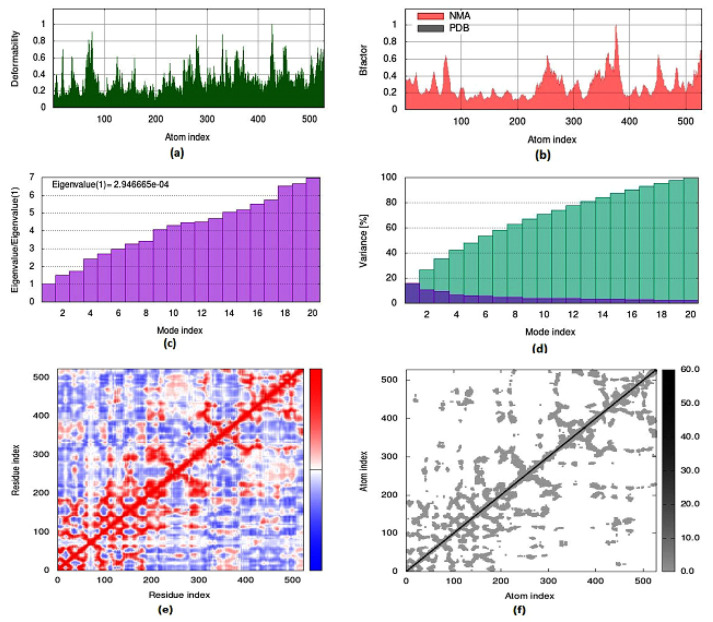
Molecular dynamics simulation of **8a** complex (BuChE) by iMODS server. (**a**) Deformability, (**b**) B-factor values, (**c**) eigenvalue, (**d**) variance, (**e**) covariance, and (**f**) elastic network model.

To evaluate the strength, movement of atoms and stiffness of the highest ranked docked pose of protein complex **8c** (AChE) and **8a** (BuChE), molecular dynamics simulation was carried out by employing the online web server iMODs (http://imods.chaconlab.org/ accessed on 20 October 2022) at a specific temperature (300 K), and constant pressure (1 atm). Molecular dynamics simulation was performed in normal mode analysis, and the results of docked complexes are displayed in Figure 15 and Figure 16. The deformability graphs of protein complexes are shown in Figure 15a and Figure 16a, with the location of different hinges (high and low) depicting the deformed region of residues in docked complexes. The experimental B-factor in Figure 15b and Figure 16b determines the value of root mean square (RMS) and reveals the unpredictability of atoms in the protein complexes. The eigenvalues of **8c** and **8a** were presented to be 1.514415 × 10^−05^ and 2.946665 × 10^−04^, respectively, as shown in Figure 15c and Figure 16c. Eigenvalue connected to normal mode exhibiting the motion inflexibility and proportional to the related binding energy to deform the globular structure of protein. Figure 15d and Figure 16d showed the variance graph of amino acid residues, which is inversely proportional to the eigenvalue. On the other hand, the covariance matrix shown in Figure 15e and Figure 16e stipulated the pairing between amino acid residues experience motions in different colors, such as red-color (correlated), white-color (uncorrelated), and blue-color (anti-correlated). The elastic model describes the coupling pair of atoms that are associated with springs. The different dots (grey color) present in the graph indicate one spring between interrelating atoms that define the inelastic springs, as shown in Figure 15f and Figure 16f.

### 2.6. Physicochemical Properties

The pharmacokinetic properties of compounds **8a**, **8c**, **8g**, and **9i** were predicted to estimate the impact of various parameters using earlier published prediction tools [53,54,55]. These parameters include molecular weight, polar surface area, number of atom types (donor/acceptor), molecular refractivity and lipophilicity i-e., the partition coefficient such as log Po/w, n-octanol, MLOGP, WLOGP, and XLOGP3, etc. illustrating the binding energies of solvation and solvent accessible surface area. The most important parameters, such as molecular weight, are that small (<500 Da), hydrophobicity of the compounds (Log P values), and polar surface area (PSA) exhibited permeability of blood–brain barrier, whereas number of HBA and HBD showed as described in Table S2. Additionally, water solubility demonstrates the solubility of the compounds in water, such as Log S (ESOL) exhibited moderate solubility, and Log S (SILICOS-IT) depicted poor solubility. Pharmacokinetics also presented P-gp substrate, cytochrome inhibitors, and Log k_p_ (skin permeation). These properties predict the drug-likeness and blood-brain barrier permeation of experimental compounds. All the selected compounds exhibit high gastrointestinal absorption, which makes them more feasible for the drug candidate. Compounds also showed up to mark efficacy with respective to drug likeliness followed by Lipinski, Ghose, Veber Egan, and Muegge rules with a bioavailability score of 0.55. The results presented in Table S3 reveal that synthetic derivatives could be developed as safe drugs.

### 2.7. Anticancer Activity

**Figure 17:**
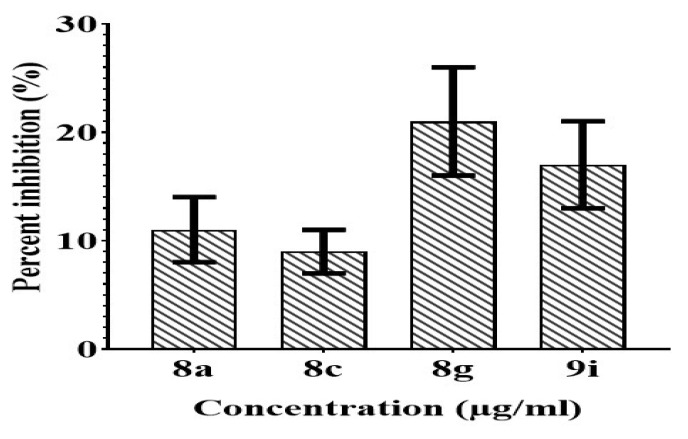
Evaluation of anticancer potential of potent compounds against Vero cell line.

The initial screening revealed that potent compounds (**8a**, **8c**, **8g**, and **9i**) exhibited less toxicity towards the normal kidney cells (Figure 17) which showed that our compounds are safe to be used as drugs. All the tested compounds displayed less than 22% inhibition.

## 3. Materials and Methods

### 3.1. General

The chemicals and solvents were purchased from Scharlau, Merck, and Fluka and were used without further purification. Ultrasonic-mediated reactions were carried out in a Clifton Ultrasonic Bath (2 x T2A, 300W, DU-4). Thin layer chromatography (TLC) was carried out on aluminum plates coated with silica gel 60 F_254_ (Merck) in an appropriate eluent, while ultraviolet light was used for visualization. Melting points were noted on the Gallenkamp melting point apparatus in open capillaries and were uncorrected. ^1^H- and ^13^C NMR spectra were recorded in DMSO-*d*_6_ on the Brücker Avance NMR instrument at 300 and 75 MHz, respectively, using TMS as an internal standard. Chemical shifts are reported as *δ* values in parts per million (ppm), and coupling constants (*J*) are given in Hertz. FTIR spectra were recorded on Bruker FTIR Spectrophotometer. High-resolution mass spectra were recorded using the electron impact (EI) technique. Elemental analysis was recorded on LECO 630-200-200 TRUSPEC CHNS micro analyzer, and the values observed were within ± 0.4% of the calculated results. Compounds **2**–**4**, **6**, and **7** were synthesized using previously reported procedures [41,43,45,46].

### 3.2. General Procedure for the Synthesis of N′-((methyl-2-(piperidin-1-yl)quinolin-3-yl)methylene)benzohydrazides (**8a-m** and **9a-j**)

#### 3.2.1. Method A: Conventional Approach

To a stirred solution of 6/8-methyl-2-(piperidin-1-yl)quinoline-3-carbaldehyde **4a** or **4b** (1 mmol) in absolute ethanol (20 mL) was added aromatic acid hydrazide **7** (1 mmol) followed by a catalytic amount of glacial acetic acid. The resulting reaction mixture was heated at reflux temperature with continuous stirring until the product precipitated out. The precipitated product was filtered, washed with hot ethanol, and dried to afford compounds **8a-m** and **9a-j**.

#### 3.2.2. Method B: Ultrasonic-Assisted Approach

The equimolar quantities of 6/8-methyl-2-(piperidin-1-yl)quinoline-3-carbaldehyde **4a** or **4b** (1 mmol) and aromatic acid hydrazide **7** (1 mmol) were dissolved in absolute ethanol (20 mL). A catalytic amount of glacial acetic acid was added to this reaction mixture and exposed to ultrasonic waves for 4–6 min at room temperature. The precipitated products were filtered, washed with hot ethanol, and dried to afford compounds **8a-m** and **9a-j**.

*N′-((6-methyl-2-(piperidin-1-yl)quinolin-3-yl)methylene)benzohydrazide* (**8a**): Yellow solid. Mp 260–262 °C. FTIR (ῡ, cm^−1^, neat): 3187 (NH), 3066 (CH-aromatic), 2960 (CH), 1641 (C=O), 1601 (C=N), 1070 (C-N); ^1^H NMR (DMSO-*d*_6_, 300 MHz) *δ* = 1.61–1.75 (m, 6H, piperidinyl-CH_2_), 2.45 (s, 3H, CH_3_), 3.19–3.22 (m, 4H, piperidinyl-N-CH_2_), 7.49–7.74 (m, 6H, ArH), 7.94 (d, *J* = 6.9 Hz, 2H, ArH), 8.52 (s, 1H, ArH), 8.61 (s, 1H, N=CH), 12.05 (s, 1H, NH); ^13^C NMR (DMSO-*d*_6_, 75 MHz) *δ* = 21.4 (CH_3_), 24.4 (piperidinyl-CH_2_), 25.9 (piperidinyl-CH_2_), 52.3 (piperidinyl-N-CH_2_), 122.0, 125.1, 127.3, 127.6, 128.2, 129.0, 132.3, 132.8, 134.0, 134.2, 135.0, 145.4, 145.8, 160.1, 163.8 (C=O); HRMS (EI) Exact mass calcd for C_23_H_25_N_4_O [M+H]^+^: 373.2025, found: 373.2021; Anal. Calcd. for C_23_H_24_N_4_O: C, 74.17; H, 6.49; N, 15.04%. Found: C, 74.25; H, 6.53; N, 15.10%.

*N′-((6-methyl-2-(piperidin-1-yl)quinolin-3-yl)methylene)-3-nitrobenzohydrazide* (**8b**): Yellow solid. Mp 228–230 °C. FTIR (ῡ, cm^−1^, neat): 3186 (NH), 3034 (CH-aromatic), 2940 (CH), 1654 (C=O), 1608 (C=N), 1077 (C-N); ^1^H NMR (DMSO-*d*_6_, 300 MHz) *δ* = 1.59–1.75 (m, 6H, piperidinyl-CH_2_), 2.45 (s, 3H, CH_3_), 3.19–3.22 (m, 4H, piperidinyl-N-CH_2_), 7.50 (dd, *J* = 8.7, 1.5 Hz, 1H, ArH), 7.53 (d, *J* = 6.9 Hz, 1H, ArH), 7.81 (d, *J* = 7.8 Hz, 1H, ArH), 7.87 (t, *J* = 8.1 Hz, 1H, ArH), 8.40 (d, *J* =7.8 Hz, 1H, ArH), 8.47 (dd, *J* = 8.1, 1.5 Hz, 1H, ArH), 8.59 (s, 1H, ArH), 8.62 (s, 1H, N=CH), 8.76 (t, *J* = 1.5 Hz, 1H, ArH), 12.32 (s, 1H, NH); ^13^C NMR (DMSO-*d*_6_, 75 MHz) *δ* = 21.4 (CH_3_), 24.4 (piperidinyl-CH_2_), 25.9 (piperidinyl-CH_2_), 52.3 (piperidinyl-N-CH_2_), 121.1, 122.8, 124.6, 124.7, 126.8, 126.9, 130.8, 134.7, 134.9, 135.3, 136.2, 146.1, 146.4, 148.3, 159.3, 161.6 (C=O); HRMS (EI) Exact mass calcd for C_23_H_24_N_5_O_3_ [M+H]^+^: 418.1877, found: 418.1875; Anal. Calcd. for C_23_H_23_N_5_O_3_: C, 66.17; H, 5.55; N, 16.78%. Found: C, 66.31; H, 5.69; N, 16.92%.

*N′-((6-methyl-2-(piperidin-1-yl)quinolin-3-yl)methylene)-4-nitrobenzohydrazide* (**8c**): Yellow solid. Mp 242–244 °C. FTIR (ῡ, cm^−1^, neat): 3210 (NH), 3047 (CH-aromatic), 2940 (CH), 1629 (C=O), 1604 (C=N), 1071 (C-N); ^1^H NMR (DMSO-*d*_6_, 300 MHz) *δ* = 1.61–1.75 (m, 6H, piperidinyl-CH_2_), 2.45 (s, 3H, CH_3_), 3.18–3.21 (m, 4H, piperidinyl-N-CH_2_), 7.50 (dd, *J* = 8.7, 1.5 Hz, 1H, ArH), 7.65–7.76 (m, 2H, ArH), 8.40 (d, *J* = 8.4 Hz, 2H, ArH), 8.56 (s, 1H, ArH), 8.61 (s, 1H, N=CH), 8.71 (d, *J* = 8.7 Hz, 2H, ArH), 12.31 (s, 1H, NH); ^13^C NMR (DMSO-*d*_6_, 75 MHz) *δ* = 21.4 (CH_3_), 24.4 (piperidinyl-CH_2_), 25.9 (piperidinyl-CH_2_), 52.3 (piperidinyl-N-CH_2_), 121.1, 122.8, 124.6, 124.7, 126.8, 126.9, 130.8, 134.7, 134.9, 135.3, 136.2, 146.4, 148.3, 159.3, 161.6 (C=O); HRMS (EI) Exact mass calcd for C_23_H_24_N_5_O_3_ [M+H]^+^: 418.1876, found: 418.1870; Anal. Calcd. for C_23_H_23_N_5_O_3_: C, 66.17; H, 5.55; N, 16.78%. Found: C, 66.29; H, 5.71; N, 16.84%.

*2-Amino-N′-((6-methyl-2-(piperidin-1-yl)quinolin-3-yl)methylene)benzohydrazide* (**8d**): Greenish yellow solid. Mp 260–262 °C. FTIR (ῡ, cm^−1^, neat): 3493, 3381 (NH_2_), 3202 (NH), 3057 (CH-aromatic), 2949 (CH), 1635 (C=O), 1587 (C=N), 1067 (C-N); ^1^H NMR (DMSO-*d*_6_, 300 MHz) *δ* = 1.60–1.75 (m, 6H, piperidinyl-CH_2_), 2.45 (s, 3H, CH_3_), 3.18–3.21 (m, 4H, piperidinyl-N-CH_2_), 6.40 (s, 2H, NH_2_), 6.61 (t, *J* = 7.5 Hz, 1H, ArH), 6.78 (d, *J* = 8.1 Hz, 1H, ArH), 7.23 (td, *J* = 7.8, 1.5 Hz, 1H, ArH), 7.48 (dd, *J* = 8.7, 1.8 Hz, 1H, ArH), 7.59 (dd, *J* = 7.8, 1.2 Hz, 1H, ArH), 7.66 (d, *J* = 8.4 Hz, 1H, ArH), 7.71 (s, 1H, ArH), 8.49 (s, 1H, ArH), 8.54 (s, 1H, N=CH), 11.85 (s, 1H, NH); ^13^C NMR (DMSO-*d*_6_, 75 MHz) *δ* = 21.4 (CH_3_), 24.4 (piperidinyl-CH_2_), 25.9 (piperidinyl-CH_2_), 52.3 (piperidinyl-N-CH_2_), 114.0, 115.1, 116.8, 122.2, 125.1, 127.3, 127.6, 128.9, 132.7, 132.8, 134.2, 134.7, 144.3, 145.7, 150.5, 160.1, 165.9 (C=O); HRMS (EI) Exact mass calcd for C_23_H_26_N_5_O [M+H]^+^: 388.2130, found: 388.2124; Anal. Calcd. for C_23_H_25_N_5_O: C, 71.29; H, 6.50; N, 18.07%. Found: C, C, 71.43; H, 6.62; N, 18.15%.

*4-Amino-N′-((6-methyl-2-(piperidin-1-yl)quinolin-3-yl)methylene)benzohydrazide* (**8e**): Greenish Yellow solid. Mp 252–254 °C. FTIR (ῡ, cm^−1^, neat): 3480, 3386 (NH_2_), 3212 (NH), 3047 (CH-aromatic), 2939 (CH), 1629 (C=O), 1605 (C=N), 1072 (C-N); ^1^H NMR (DMSO-*d*_6_, 300 MHz) *δ* = 1.61–1.75 (m, 6H, piperidinyl-CH_2_), 2.44 (s, 3H, CH_3_), 3.18–3.21 (m, 4H, piperidinyl-N-CH_2_), 5.83 (s, 2H, NH_2_), 6.62 (d, *J* = 8.4 Hz, 2H, ArH), 7.48 (dd, *J* = 8.7, 1.8 Hz, 1H, ArH), 7.63–7.71 (m, 4H, ArH), 8.46 (s, 1H, ArH), 8.54 (s, 1H, N=CH), 11.68 (s, 1H, NH); ^13^C NMR (DMSO-*d*_6_, 75 MHz) *δ* = 21.4 (CH_3_), 24.4 (piperidinyl-CH_2_), 25.9 (piperidinyl-CH_2_), 52.2 (piperidinyl-N-CH_2_), 113.0, 119.9, 122.4, 125.1, 127.3, 127.5, 129.9, 130.0, 132.6, 134.2, 134.5, 143.4, 145.6, 152.9, 160.1 (C=O); HRMS (EI) Exact mass calcd for C_23_H_26_N_5_O [M+H]^+^: 388.2131, found: 388.2127; Anal. Calcd. for C_23_H_25_N_5_O: C, 71.29; H, 6.50; N, 18.07%. Found: C, 71.17; H, 6.32; N, 18.01%.

*2-Chloro-N′-((6-methyl-2-(piperidin-1-yl)quinolin-3-yl)methylene)benzohydrazide* (**8f**): Light yellow solid. Mp 244–246 °C. FTIR (ῡ, cm^−1^, neat): 3186 (NH), 3060 (CH-aromatic), 2918 (CH), 1658 (C=O), 1575 (C=N), 1050 (C-N); ^1^H NMR (DMSO-*d*_6_, 300 MHz) *δ* = 1.59–1.72 (m, 6H, piperidinyl-CH_2_), 2.45 (s, 3H, CH_3_), 3.16–3.19 (m, 4H, piperidinyl-N-CH_2_), 7.49–7.65 (m, 6H, ArH), 7.75 (s, 1H, ArH), 8.44 (s, 1H, ArH), 8.55 (s, 1H, N=CH), 12.14 (s, 1H, NH); ^13^C NMR (DMSO-*d*_6_, 75 MHz) *δ* = 21.4 (CH_3_), 24.4 (piperidinyl-CH_2_), 25.9 (piperidinyl-CH_2_), 52.3 (piperidinyl-N-CH_2_), 121.6, 125.1, 127.3, 127.8, 129.9, 130.3, 130.9, 132.0, 132.9, 134.3, 135.0, 135.6, 145.6, 145.9, 160.1, 162.9, 169.0 (C=O); HRMS (EI) Exact mass calcd for C_23_H_24_ClN_4_O [M+H]^+^: 407.1638, found: 407.1629; Anal. Calcd. for C_23_H_23_ClN_4_O: C, 67.89; H, 5.70; N, 13.77%. Found: C, 68.01; H, 5.84; N, 13.91%.

*3-Chloro-N′-((6-methyl-2-(piperidin-1-yl)quinolin-3-yl)methylene)benzohydrazide* (**8g**): Yellow solid. Mp 216–218 °C. FTIR (ῡ, cm^−1^, neat): 3178 (NH), 3066 (CH-aromatic), 2921 (CH), 1655 (C=O), 1589 (C=N), 1049 (C-N); ^1^H NMR (DMSO-*d*_6_, 300 MHz) *δ* = 1.59–1.75 (m, 6H, piperidinyl-CH_2_), 2.45 (s, 3H, CH_3_), 3.19–3.22 (m, 4H, piperidinyl-N-CH_2_), 7.50 (dd, *J* = 8.7, 1.5 Hz, 1H, ArH), 7.60 (t, *J* = 7.8 Hz, 1H, ArH), 7.65–7.74 (m, 3H, ArH), 7.91 (d, *J* = 7.8 Hz, 1H, ArH), 7.98 (t, *J* = 1.5 Hz, 1H, ArH), 8.52 (s, 1H, ArH), 8.60 (s, 1H, N=CH), 12.13 (s, 1H, NH); ^13^C NMR (DMSO-*d*_6_, 75 MHz) *δ* = 21.4 (CH_3_), 24.4 (piperidinyl-CH_2_), 25.9 (piperidinyl-CH_2_), 52.3 (piperidinyl-N-CH_2_), 121.8, 125.1, 127.0, 127.3, 127.7, 127.8, 131.1, 132.1, 132.9, 133.8, 134.3, 135.1, 135.9, 145.8, 145.9, 160.1, 162.4 (C=O); HRMS (EI) Exact mass calcd for C_23_H_24_ClN_4_O [M+H]^+^: 407.1632, found: 407.1630; Anal. Calcd. for C_23_H_23_ClN_4_O: C, 67.89; H, 5.70; N, 13.77%. Found: C, 67.95; H, 5.74; N, 13.89%.

*4-Chloro-N′-((6-methyl-2-(piperidin-1-yl)quinolin-3-yl)methylene)benzohydrazide* (**8h**): Yellow solid. Mp 250–252 °C. FTIR (ῡ, cm^−1^, neat): 3200 (NH), 3069 (CH-aromatic), 2939 (CH), 1644 (C=O), 1593 (C=N), 1065 (C-N); ^1^H NMR (DMSO-*d*_6_, 300 MHz) *δ* = 1.59–1.75 (m, 6H, piperidinyl-CH_2_), 2.45 (s, 3H, CH_3_), 3.19–3.22 (m, 4H, piperidinyl-N-CH_2_), 7.50 (dd, *J* = 8.7, 1.5 Hz, 1H, ArH), 7.64 (d, *J* = 8.1 Hz, 2H, ArH), 7.66–7.74 (m, 2H, ArH), 7.97 (d, *J* = 8.4 Hz, 2H, ArH), 8.52 (s, 1H, ArH), 8.60 (s, 1H, N=CH), 12.11 (s, 1H, NH); ^13^C NMR (DMSO-*d*_6_, 75 MHz) *δ* = 21.4 (CH_3_), 24.4 (piperidinyl-CH_2_), 25.9 (piperidinyl-CH_2_), 52.3 (piperidinyl-N-CH_2_), 121.9, 125.1, 127.3, 127.6, 129.1, 130.1, 132.6, 132.8, 134.3, 135.1, 137.1, 145.7, 145.8, 160.1, 162.7 (C=O); HRMS (EI) Exact mass calcd for C_23_H_24_ClN_4_O [M+H]^+^: 407.1631, found: 407.1633; Anal. Calcd. for C_23_H_23_ClN_4_O: C, 67.89; H, 5.70; N, 13.77%. Found: C, 67.75; H, 5.56; N, 13.59%.

*2,4-Dichloro-N′-((6-methyl-2-(piperidin-1-yl)quinolin-3-yl)methylene)benzohydrazide* (**8i**): Light yellow solid. Mp 240–242 °C. FTIR (ῡ, cm^−1^, neat): 3184 (NH), 3055 (CH-aromatic), 2944 (CH), 1662 (C=O), 1590 (C=N), 1073 (C-N); ^1^H NMR (DMSO-*d*_6_, 300 MHz) *δ* = 1.59–1.72 (m, 6H, piperidinyl-CH_2_), 2.45 (s, 3H, CH_3_), 3.16–3.19 (m, 4H, piperidinyl-N-CH_2_), 7.50 (dd, *J* = 8.7, 1.5 Hz, 1H, ArH), 7.57–7.77 (m, 4H, ArH), 7.82 (d, *J* = 1.8 Hz, 1H, ArH), 8.44 (s, 1H, ArH), 8.55 (s, 1H, N=CH), 12.16 (s, 1H, NH); ^13^C NMR (DMSO-*d*_6_, 75 MHz) *δ* = 21.4 (CH_3_), 24.4 (piperidinyl-CH_2_), 25.9 (piperidinyl-CH_2_), 52.4 (piperidinyl-N-CH_2_), 121.5, 125.1, 127.3, 127.7, 128.1, 129.9, 131.0, 131.3, 132.3, 133.0, 134.3, 134.4, 135.8, 142.9, 145.9, 160.1, 162.0 (C=O); HRMS (EI) Exact mass calcd for C_23_H_23_Cl_2_N_4_O [M+H]^+^: 441.1245, found: 441.1240; Anal. Calcd. for C_23_H_22_Cl_2_N_4_O: C, 62.59; H, 5.02; N, 12.69%. Found: C, 62.81; H, 5.28; N, 12.85%.

*2-Methoxy-N′-((6-methyl-2-(piperidin-1-yl)quinolin-3-yl)methylene)benzohydrazide* (**8j**): Yellow solid. Mp 228–230 °C. FTIR (ῡ, cm^−1^, neat): 3182 (NH), 3051 (CH-aromatic), 2930 (CH), 1636 (C=O), 1596 (C=N), 1246 (C-O), 1070 (C-N); ^1^H NMR (DMSO-*d*_6_, 300 MHz) *δ* = 1.60–1.74 (m, 6H, piperidinyl-CH_2_), 2.44 (s, 3H, CH_3_), 3.18–3.21 (m, 4H, piperidinyl-N-CH_2_), 3.85 (s, 3H, OCH_3_), 7.19 (ddd, *J* = 7.2, 2.4, 1.2 Hz, 1H, ArH), 7.45–7.53 (m, 4H, ArH), 7.66 (d, *J* = 8.4 Hz, 1H, ArH), 7.72 (s, 1H, ArH), 8.51 (s, 1H, ArH), 8.61 (s, 1H, N=CH), 12.03 (s, 1H, NH); ^13^C NMR (DMSO-*d*_6_, 75 MHz) *δ* = 21.4 (CH_3_), 24.4 (piperidinyl-CH_2_), 25.9 (piperidinyl-CH_2_), 52.3 (piperidinyl-N-CH_2_), 55.8, 113.5, 117.9, 120.3, 122.0, 125.1, 127.3, 127.6, 130.2, 132.8, 134.2, 135.0, 135.3, 145.5, 145.8, 159.7, 160.1, 163.6 (C=O); HRMS (EI) Exact mass calcd for C_24_H_27_N_4_O_2_ [M+H]^+^: 403.2134, found: 403.2125; Anal. Calcd. for C_24_H_26_N_4_O_2_: C, 71.62; H, 6.51; N, 13.92%. Found: C, 71.78; H, 6.67; N, 14.08%.

*3-Methoxy-N′-((6-methyl-2-(piperidin-1-yl)quinolin-3-yl)methylene)benzohydrazide* (**8k**): Yellow solid. Mp 190–192 °C. FTIR (ῡ, cm^−1^, neat): 3181 (NH), 3051 (CH-aromatic), 2931 (CH), 1635 (C=O), 1595 (C=N), 1245 (C-O), 1071 (C-N); ^1^H NMR (DMSO-*d*_6_, 300 MHz) *δ* = 1.58–1.73 (m, 6H, piperidinyl-CH_2_), 2.45 (s, 3H, CH_3_), 3.17–3.21 (m, 4H, piperidinyl-N-CH_2_), 3.87 (s, 3H, OCH_3_), 7.07 (t, *J* = 7.5 Hz, 1H, ArH), 7.17 (d, *J* = 8.4 Hz, 1H, ArH), 7.48–7.58 (m, 3H, ArH), 7.66 (d, *J* = 8.4 Hz, 1H, ArH), 7.73 (s, 1H, ArH), 8.45 (s, 1H, ArH), 8.51 (s, 1H, N=CH), 11.75 (s, 1H, NH); ^13^C NMR (DMSO-*d*_6_, 75 MHz) *δ* = 21.4 (CH_3_), 24.4 (piperidinyl-CH_2_), 25.9 (piperidinyl -CH_2_), 52.3 (piperidinyl-N-CH_2_), 56.2, 112.4, 120.9, 121.9, 124.6, 125.1, 127.3, 127.6, 130.1, 132.5, 132.8, 134.2, 134.9, 144.7, 145.8, 157.1, 160.1, 163.1 (C=O); HRMS (EI) Exact mass calcd for C_24_H_27_N_4_O_2_ [M+H]^+^: 403.2128, found: 403.2131; Anal. Calcd. for C_24_H_26_N_4_O_2_: C, 71.62; H, 6.51; N, 13.92%. Found: C, 71.44; H, 6.39; N, 13.80%.

*4-Methoxy-N′-((6-methyl-2-(piperidin-1-yl)quinolin-3-yl)methylene)benzohydrazide* (**8l**): Greenish yellow solid. Mp 248–250 °C. FTIR (ῡ, cm^−1^, neat): 3209 (NH), 3051 (CH-aromatic), 2955 (CH), 1640 (C=O), 1601 (C=N), 1247 (C-O), 1068 (C-N); ^1^H NMR (DMSO-*d*_6_, 300 MHz) *δ* = 1.62–1.76 (m, 6H, piperidinyl-CH_2_), 2.46 (s, 3H, CH_3_), 3.20–3.23 (m, 4H, piperidinyl-N-CH_2_), 3.86 (s, 3H, OCH_3_), 7.10 (d, *J* = 9.0 Hz, 2H, ArH), 7.50 (dd, *J* = 8.4, 1.5 Hz, 1H, ArH), 7.67 (d, *J* = 8.4 Hz, 1H, ArH), 7.74 (d, *J* = 0.9 Hz, 1H, ArH), 7.94 (d, *J* = 8.7 Hz, 2H, ArH), 8.51 (s, 1H, ArH), 8.60 (s, 1H, N=CH), 11.94 (s, 1H, NH); ^13^C NMR (DMSO-*d*_6_, 75 MHz) *δ* = 21.4 (CH_3_), 24.4 (piperidinyl-CH_2_), 25.9 (piperidinyl-CH_2_), 52.3 (piperidinyl-N-CH_2_), 55.9, 114.2, 122.1, 125.1, 125.9, 127.3, 127.6, 130.1, 132.7, 134.2, 134.8, 144.7, 145.7, 160.1, 162.5, 163.2 (C=O); HRMS (EI) Exact mass calcd for C_24_H_27_N_4_O_2_ [M+H]^+^: 403.2132, found: 403.2126; Anal. Calcd. for C_24_H_26_N_4_O_2_: C, 71.62; H, 6.51; N, 13.92%. Found: C, 71.60; H, 6.45; N, 13.86%.

*2-Hydroxy-N′-((6-methyl-2-(piperidin-1-yl)quinolin-3-yl)methylene)benzohydrazide* (**8m**): Yellow solid. Mp 210–212 °C. FT-IR (ῡ cm^−1^; neat): 3405 (OH), 3209 (NH), 3047 (CH-Aromatic), 2939 (CH), 1629 (C=O), 1604 (C=N), 1070 (C-N); ^1^H NMR (DMSO-*d*_6_, 300 MHz) δ = 1.59–1.75 (m, 6H, Piperidinyl -CH_2_-), 2.45 (s, 3H, -CH_3_), 3.20–3.23 (m, 4H, Piperidinyl N-CH_2_-), 6.96–7.01 (m, 2H, ArH), 7.46 (dd, *J* = 8.1 Hz, 1.5 Hz, 1H, ArH), 7.51 (dd, *J* = 8.4 Hz, 1.8 Hz, 1H, ArH), 7.67 (d, *J* = 8.4 Hz, 1H, ArH), 7.74 (s, 1H, ArH), 7.89 (dd, *J* = 8.1 Hz, 2.1 Hz, 1H, ArH), 8.53 (s, 1H, ArH), 8.60 (s, 1H, -N=CH-), 11.81 (s, 1H, -OH), 12.07 (s, 1H, -NH-) ppm. ^13^C NMR (DMSO-*d*_6_, 75 MHz) δ = 21.4 (-CH_3_), 24.4 (Piperidinyl -CH_2_), 25.9 (2C; Piperidinyl -CH_2_), 52.3 (2C; Piperidinyl-N-CH_2_), 116.5, 117.8, 119.4, 121.8, 125.1, 127.3, 127.7, 128.9, 132.9, 134.2, 134.3, 135.2, 145.9, 146.3, 159.6, 160.1, 165.6 (C=O) ppm; HRMS (EI) Exact mass calcd for C_23_H_25_N_4_O_2_ [M+H]^+^: 389.1975, found: 389.1980; Anal. Calcd. for C_23_H_24_N_4_O_2_: C, 71.11; H, 6.23; N, 14.42%. Found: C, 71.33; H, 6.49; N, 14.60%.

*N′-((8-methyl-2-(piperidin-1-yl)quinolin-3-yl)methylene)benzohydrazide* (**9a**): Yellow solid. Mp 254–256 °C. FTIR (ῡ, cm^−1^, neat): 3186 (NH), 3065 (CH-aromatic), 2960 (CH), 1642 (C=O), 1553 (C=N), 1070 (C-N); ^1^H NMR (CDCl_3_, 300 MHz) *δ* = 1.66–1.83 (m, 6H, piperidinyl-CH_2_), 2.72 (s, 3H, CH_3_), 3.34–3.37 (m, 4H, piperidinyl-N-CH_2_), 7.25–7.30 (m, 1H, ArH), 7.49–7.64 (m, 5H, ArH), 7.91–7.95 (m, 2H, ArH), 8.46 (s, 1H, ArH), 8.70 (s, 1H, N=CH), 9.27 (s, 1H, NH); ^13^C NMR (DMSO-*d*_6_, 75 MHz) *δ* = 17.8 (CH_3_), 24.5 (piperidinyl-CH_2_), 25.8 (piperidinyl-CH_2_), 52.1 (piperidinyl-N-CH_2_), 114.5, 114.2, 121.5, 124.6, 124.8, 125.9, 126.6, 130.1, 130.6, 134.9, 135.8, 144.7, 145.9, 159.3, 162.5 (C=O); HRMS (EI) Exact mass calcd for C_23_H_25_N_4_O [M+H]^+^: 373.2028, found: 373.2024; Anal. Calcd. for C_23_H_24_N_4_O: C, 74.17; H, 6.49; N, 15.04%. Found: C, 74.21; H, 6.57; N, 15.16%.

*N′-((8-methyl-2-(piperidin-1-yl)quinolin-3-yl)methylene)-3-nitrobenzohydrazide* (**9b**): Yellow solid. Mp 226–228 °C. FTIR (ῡ, cm^−1^, neat): 3186 (NH), 3033 (CH-aromatic), 2939 (CH), 1654 (C=O), 1609 (C=N), 1078 (C-N); ^1^H NMR (DMSO-*d*_6_, 300 MHz) *δ* = 1.64–1.77 (m, 6H, piperidinyl-CH_2_), 2.63 (s, 3H, CH_3_), 3.28–3.31 (m, 4H, piperidinyl-N-CH_2_), 7.31 (t, *J* = 7.5 Hz, 1H, ArH), 7.53 (d, *J* = 6.9 Hz, 1H, ArH), 7.81 (d, *J* = 7.8 Hz, 1H, ArH), 7.87 (t, *J* = 8.1 Hz, 1H, ArH), 8.40 (d, *J* =7.8 Hz, 1H, ArH), 8.47 (dd, *J* = 8.1, 1.5 Hz, 1H, ArH), 8.59 (s, 1H, ArH), 8.63 (s, 1H, N=CH), 8.77 (t, *J* = 1.5 Hz, 1H, ArH), 12.33 (s, 1H, NH); ^13^C NMR (DMSO-*d*_6_, 75 MHz) *δ* = 17.8 (CH_3_), 24.5 (piperidinyl-CH_2_), 25.8 (piperidinyl-CH_2_), 52.2 (piperidinyl-N-CH_2_), 121.1, 122.8, 124.6, 124.7, 126.8, 126.9, 130.8, 134.7, 134.9, 135.3, 136.2, 146.1, 146.4, 148.3, 159.3, 161.6 (C=O); HRMS (EI) Exact mass calcd for C_23_H_24_N_5_O_3_ [M+H]^+^: 418.1870, found: 418.1864; Anal. Calcd. for C_23_H_23_N_5_O_3_: C, 66.17; H, 5.55; N, 16.78%. Found: C, 66.25; H, 5.59; N, 16.86%.

*N′-((8-methyl-2-(piperidin-1-yl)quinolin-3-yl)methylene)-4-nitrobenzohydrazide* (**9c**): Yellow solid. Mp 248–250 °C. FTIR (ῡ, cm^−1^, neat): 3209 (NH), 3047 (CH-aromatic), 2939 (CH), 1630 (C=O), 1604 (C=N), 1071 (C-N); ^1^H NMR (DMSO-*d*_6_, 300 MHz) *δ* = 1.61–1.76 (m, 6H, piperidinyl-CH_2_), 2.62 (s, 3H, CH_3_), 3.25–3.29 (m, 4H, piperidinyl-N-CH_2_), 7.31 (t, *J* = 7.5 Hz, 1H, ArH), 7.53 (d, *J* = 6.9 Hz, 1H, ArH), 7.81 (d, *J* = 7.8 Hz, 1H, ArH), 8.40 (d, *J* = 7.8 Hz, 2H, ArH), 8.54 (s, 1H, ArH), 8.60 (s, 1H, N=CH), 8.75 (d, *J* = 8.4 Hz, 2H, ArH), 12.30 (s, 1H, NH); ^13^C NMR (DMSO-*d*_6_, 75 MHz) *δ* = 17.8 (CH_3_), 24.5 (piperidinyl-CH_2_), 25.8 (piperidinyl-CH_2_), 52.2 (piperidinyl-N-CH_2_), 121.1, 122.8, 124.6, 124.7, 126.8, 126.9, 130.8, 134.7, 134.9, 135.3, 136.2, 146.4, 148.3, 159.3, 163.6 (C=O); HRMS (EI) Exact mass calcd for C_23_H_24_N_5_O_3_ [M+H]^+^: 418.1873, found: 418.1871; Anal. Calcd. for C_23_H_23_N_5_O_3_: C, 66.17; H, 5.55; N, 16.78%. Found: C, 66.43; H, 5.79; N, 16.90%.

*2-Chloro-N′-((8-methyl-2-(piperidin-1-yl)quinolin-3-yl)methylene)benzohydrazide* (**9d**): Yellow solid. Mp 248–250 °C. FTIR (ῡ, cm^−1^, neat): 3187 (NH), 3061 (CH-aromatic), 2918 (CH), 1659 (C=O), 1560 (C=N), 1050 (C-N); ^1^H NMR (DMSO-*d*_6_, 300 MHz) *δ* = 1.59–1.73 (m, 6H, piperidinyl-CH_2_), 2.62 (s, 3H, CH_3_), 3.23–3.26 (m, 4H, piperidinyl-N-CH_2_), 7.31 (t, *J* = 7.5 Hz, 1H, ArH), 7.47–7.65 (m, 5H, ArH), 7.82 (d, *J* = 7.8 Hz, 1H, ArH), 8.44 (s, 1H, ArH), 8.62 (s, 1H, N=CH), 12.14 (s, 1H, NH); ^13^C NMR (DMSO-*d*_6_, 75 MHz) *δ* = 17.8 (CH_3_), 24.5 (piperidinyl-CH_2_), 25.8 (piperidinyl-CH_2_), 52.2 (piperidinyl-N-CH_2_), 121.0, 124.6, 124.8, 126.8, 127.8, 129.5, 129.9, 130.3, 130.9, 132.0, 134.9, 135.6, 136.0, 145.6, 146.1, 159.4, 162.9 (C=O); HRMS (EI) Exact mass calcd for C_23_H_24_ClN_4_O [M+H]^+^: 407.1630, found: 407.1633; Anal. Calcd. for C_23_H_23_ClN_4_O: C, 67.89; H, 5.70; N, 13.77%. Found: C, 68.07; H, 5.94; N, 13.91%.

*3-Chloro-N′-((8-methyl-2-(piperidin-1-yl)quinolin-3-yl)methylene)benzohydrazide* (**9e**): Yellow solid. Mp 220–222 °C. FTIR (ῡ, cm^−1^, neat): 3200 (NH), 3068 (CH-aromatic), 2940 (CH), 1645 (C=O), 1600 (C=N), 1066 (C-N); ^1^H NMR (DMSO-*d*_6_, 300 MHz) *δ* = 1.60–1.75 (m, 6H, piperidinyl-CH_2_), 2.62 (s, 3H, CH_3_), 3.26–3.29 (m, 4H, piperidinyl-N-CH_2_), 7.30 (t, *J* = 7.5 Hz, 1H, ArH), 7.52 (d, *J* = 6.9 Hz, 1H, ArH), 7.58 (t, *J* = 7.8 Hz, 1H, ArH), 7.68 (t, *J* = 8.4 Hz, 1H, ArH), 7.78 (d, *J* = 8.1 Hz, 1H, ArH), 7.92 (d, *J* = 7.8 Hz, 1H, ArH), 8.00 (t, *J* = 1.5 Hz, 1H, ArH), 8.56 (s, 1H, ArH), 8.59 (s, 1H, N=CH), 12.20 (s, 1H, NH); ^13^C NMR (DMSO-*d*_6_, 75 MHz) *δ* = 17.8 (CH_3_), 24.5 (piperidinyl-CH_2_), 25.8 (piperidinyl-CH_2_), 52.1 (piperidinyl-N-CH_2_), 121.3, 124.6, 124.8, 126.7, 127.0, 127.9, 130.7, 131.0, 132.0, 133.7, 134.9, 136.0, 145.8, 146.0, 162.5 (C=O); HRMS (EI) Exact mass calcd for C_23_H_24_ClN_4_O [M+H]^+^: 407.1635, found: 407.1628; Anal. Calcd. for C_23_H_23_ClN_4_O: C, 67.89; H, 5.70; N, 13.77%. Found: C, 67.93; H, 5.82; N, 13.83%.

*4-Chloro-N′-((8-methyl-2-(piperidin-1-yl)quinolin-3-yl)methylene)benzohydrazide* (**9f**): Yellow solid. Mp 198–200 °C. FTIR (ῡ, cm^−1^, neat): 3201 (NH), 3068 (CH-aromatic), 2939 (CH), 1644 (C=O), 1594 (C=N), 1065 (C-N); ^1^H NMR (DMSO-*d*_6_, 300 MHz) *δ* = 1.65–1.76 (m, 6H, piperidinyl-CH_2_), 2.63 (s, 3H, CH_3_), 3.27–3.30 (m, 4H, piperidinyl-N-CH_2_), 7.31 (t, *J* = 7.5 Hz, 1H, ArH), 7.53 (d, *J* = 6.6 Hz, 1H, ArH), 7.64 (d, *J* = 8.4 Hz, 2H, ArH), 7.81 (d, *J* = 7.8 Hz, 1H, ArH), 7.97 (d, *J* = 8.4 Hz, 2H, ArH), 8.58 (s, 1H, ArH), 8.61 (s, 1H, N=CH), 12.12 (s, 1H, NH); ^13^C NMR (DMSO-*d*_6_, 75 MHz) δ = 17.8 (CH_3_), 24.5 (piperidinyl-CH_2_), 25.8 (piperidinyl-CH_2_), 52.1 (piperidinyl-N-CH_2_), 121.3, 124.6, 124.8, 126.7, 129.1, 130.1, 130.8, 132.6, 134.9, 136.0, 137.1, 145.6, 146.0, 159.4, 162.7 (C=O); HRMS (EI) Exact mass calcd for C_23_H_24_ClN_4_O [M+H]^+^: 407.1632, found: 407.1631; Anal. Calcd. for C_23_H_23_ClN_4_O: C, 67.89; H, 5.70; N, 13.77%. Found: C, 67.98; H, 5.74; N, 13.91%.

*2,4-Dichloro-N′-((8-methyl-2-(piperidin-1-yl)quinolin-3-yl)methylene)benzohydrazide* (**9g**): Yellow solid. Mp 232–234 °C. FTIR (ῡ, cm^−1^, neat): 3187 (NH), 3055 (CH-aromatic), 2944 (CH), 1663 (C=O), 1589 (C=N), 1072 (C-N); ^1^H NMR (DMSO-*d*_6_, 300 MHz) *δ* = 1.61–1.73 (m, 6H, piperidinyl-CH_2_), 2.62 (s, 3H, CH_3_), 3.23–3.26 (m, 4H, piperidinyl-N-CH_2_), 7.31 (t, *J* = 7.5 Hz, 1H, ArH), 7.52–7.61 (m, 2H, ArH), 7.68 (d, *J* = 8.4 Hz, 1H, ArH), 7.80–7.82 (m, 1H, ArH), 7.98 (s, 1H, ArH), 8.43 (s, 1H, ArH), 8.61 (s, 1H, N=CH), 12.15 (s, 1H, NH); ^13^C NMR (DMSO-*d*_6_, 75 MHz) *δ* = 17.8 (CH_3_), 24.5 (piperidinyl-CH_2_), 25.8 (piperidinyl-CH_2_), 52.2 (piperidinyl-N-CH_2_), 120.9, 124.6, 124.8, 126.8, 128.0, 129.9, 131.3, 132.3, 134.4, 135.0, 135.8, 136.0, 145.9, 159.4, 162.0 (C=O); HRMS (EI) Exact mass calcd for C_23_H_23_Cl_2_N_4_O [M+H]^+^: 441.1247, found: 441.1243; Anal. Calcd. for C_23_H_22_Cl_2_N_4_O: C, 62.59; H, 5.02; N, 12.69%. Found: C, 62.81; H, 5.34; N, 12.87%.

*2-Methoxy-N′-((8-methyl-2-(piperidin-1-yl)quinolin-3-yl)methylene)benzohydrazide* (**9h**): Light yellow solid. Mp 198–200 °C. FTIR (ῡ, cm^−1^, neat): 3119 (NH), 3050 (CH-aromatic), 2930 (CH), 1635 (C=O), 1595 (C=N), 1245 (C-O), 1070 (C-N); ^1^H NMR (DMSO-*d*_6_, 300 MHz) *δ* = 1.50–1.76 (m, 6H, piperidinyl-CH_2_), 2.63 (s, 3H, CH_3_), 3.27–3.30 (m, 4H, piperidinyl-N-CH_2_), 3.85 (s, 3H, OCH_3_), 7.19 (d, *J* = 7.8 Hz, 1H, ArH), 7.31 (t, *J* = 7.5 Hz, 1H, ArH), 7.46–7.54 (m, 4H, ArH), 7.81 (d, *J* = 7.8 Hz, 1H, ArH), 8.57 (s, 1H, ArH), 8.61 (s, 1H, N=CH), 12.02 (s, 1H, NH); ^13^C NMR (DMSO-*d*_6_, 75 MHz) *δ* = 17.8 (CH_3_), 24.5 (piperidinyl-CH_2_), 25.8 (piperidinyl-CH_2_), 52.1 (piperidinyl-N-CH_2_), 55.9, 113.5, 117.9, 120.3, 121.3, 124.6, 124.8, 126.7, 130.2, 130.7, 134.9, 135.4, 136.0, 145.4, 146.0, 159.3, 159.7, 163.5 (C=O); HRMS (EI) Exact mass calcd for C_24_H_27_N_4_O_2_ [M+H]^+^: 403.2130, found: 403.2127; Anal. Calcd. for C_24_H_26_N_4_O_2_: C, 71.62; H, 6.51; N, 13.92%. Found: C, 71.50; H, 6.29; N, 13.77%.

*3-Methoxy-N′-((8-methyl-2-(piperidin-1-yl)quinolin-3-yl)methylene)benzohydrazide* (**9i**): Yellow solid. Mp 194–196 °C. FTIR (ῡ, cm^−1^, neat): 3255 (NH), 3004 (CH-aromatic), 2931 (CH), 1650 (C=O), 1605 (C=N), 1224 (C-O), 1045 (C-N); ^1^H NMR (DMSO-*d*_6_, 300 MHz) *δ* = 1.61–1.74 (m, 6H, piperidinyl-CH_2_), 2.62 (s, 3H, CH_3_), 3.25–3.28 (m, 4H, piperidinyl-N-CH_2_), 3.87 (s, 3H, OCH_3_), 7.07 (t, *J* = 7.5 Hz, 1H, ArH), 7.18 (d, *J* = 8.1 Hz, 1H, ArH), 7.30 (t, *J* = 7.5 Hz, 1H, ArH), 7.46–7.59 (m, 3H, ArH), 7.80 (d, *J* = 8.1 Hz, 1H, ArH), 8.45 (s, 1H, ArH), 8.57 (s, 1H, N=CH), 11.74 (s, 1H, NH); ^13^C NMR (DMSO-*d*_6_, 75 MHz) *δ* = 17.8 (CH_3_), 24.5 (piperidinyl-CH_2_), 25.8 (piperidinyl-CH_2_), 52.1 (piperidinyl-N-CH_2_), 56.2, 112.4, 120.9, 121.3, 124.6, 124.8, 126.7, 130.1, 130.7, 132.5, 134.9, 135.9, 144.7, 146.0, 157.1, 159.3, 163.1 (C=O); HRMS (EI) Exact mass calcd for C_24_H_27_N_4_O_2_ [M+H]^+^: 403.2135, found: 403.2125; Anal. Calcd. for C_24_H_26_N_4_O_2_: C, 71.62; H, 6.51; N, 13.92%. Found: C, 71.70; H, 6.65; N, 14.10%.

*4-Methoxy-N′-((8-methyl-2-(piperidin-1-yl)quinolin-3-yl)methylene)benzohydrazide* (**9j**): Yellow solid. Mp 242–244 °C. FTIR (ῡ, cm^−1^, neat): 3251 (NH), 3049 (CH-aromatic), 2930 (CH), 1636 (C=O), 1596 (C=N), 1245 (C-O), 1071 (C-N); ^1^H NMR (DMSO-*d*_6_, 300 MHz) *δ* = 1.61–1.77 (m, 6H, piperidinyl-CH_2_-), 2.63 (s, 3H, CH_3_), 3.27–3.30 (m, 4H, piperidinyl-N-CH_2_), 3.85 (s, 3H, OCH_3_), 7.09 (d, *J* = 9.0 Hz, 2H, ArH), 7.30 (t, *J* = 7.5 Hz, 1H, ArH), 7.52 (d, *J* = 6.9 Hz, 1H, ArH), 7.79 (d, *J* = 7.8 Hz, 1H, ArH), 7.94 (d, *J* = 8.7 Hz, 2H, ArH), 8.56 (s, 1H, ArH), 8.60 (s, 1H, N=CH), 11.93 (s, 1H, NH); ^13^C NMR (DMSO-*d*_6_, 75 MHz) *δ* = 17.8 (CH_3_), 24.5 (piperidinyl-CH_2_), 25.8 (piperidinyl-CH_2_), 52.1 (piperidinyl-N-CH_2_), 55.9, 114.2, 114.5, 121.5, 124.6, 124.8, 125.9, 126.7, 130.1, 130.6, 134.9, 135.8, 144.7, 145.9, 159.3, 162.5 (C=O); HRMS (EI) Exact mass calcd for C_24_H_27_N_4_O_2_ [M+H]^+^: 403.2131, found: 403.2130; Anal. Calcd. for C_24_H_26_N_4_O_2_: C, 71.62; H, 6.51; N, 13.92%. Found: C, 71.88; H, 6.73; N, 14.14%.

### 3.3. In Vitro Cholinesterase Inhibition Assays

The inhibitory activity of synthesized compounds against acetylcholinesterase and butyrylcholinesterase was determined by Ellman spectrophotometric method with slight modification [48,49]. The reaction contents contained 60 µL KH_2_PO_4_ or KOH buffers, pH (7.7), 10 µL of synthesized compounds solvated in 2% DMSO, and 10 µL of enzyme (AChE, 0.5 U/mg, and BuChE, 3.4 U/mg). The reaction mixture was mixed completely and pre-incubated for 10 min at 37 °C. To start the reaction, 10 µL of 1 mM substrate, acetylthiocholine chloride, and butyrylthiocholine chloride were added to AChE and BuChE enzyme solution. A coloring reagent (DTNB 10 µL, 0.5 mM) was also added to the reaction mixture. Absorbance was determined at wavelength (405 nm) by a 96-well microplate reader after the incubation for 25 min at 37 °C. All experiments were performed in triplicate. To estimate the inhibitory activity of enzyme, the following formula was used, and percent inhibition was calculated.

Inhibition (%) = 100 − (A_c_/A_s_) × 100 (1)

A_c_ and A_s_ are absorbance estimated for AChE and BuChE enzymes in the presence or absence of inhibitors. IC_50_ value was calculated for tested compounds exhibiting more than 50% inhibition against AChE and BuChE through non-linear Prism 5.0 (GraphPad, San Diego, CA, USA).

### 3.4. Molecular Docking Protocols

#### 3.4.1. Structure Selection and Preparation

To investigate the possible interactions of the potent compounds forming complex with AChE or BuChE, molecular docking was performed. RCBS PDB database [56] was used to obtain the crystallographic structure of electric eel AChE (PDB ID:4BDT) and *h*BuChE (PDB ID:4BDS) to perform docking studies. Before the experiments, respective structures of enzymes and potent compounds were prepared. Protonate3D [57] utility within the modeling tool MOE [58] was used to protonate the enzyme structure. The structural energy of the enzyme was minimized by using the Amber99 force field, which includes all solvent molecules. During energy calculations, structurally important atoms were confined with mini force to avoid subsiding of the binding pockets. Then, co-crystallized ligands and crystallographic water molecules (solvent) were removed. Subsequently, polar hydrogen atoms were added to the stranded structure of protein with the MOE.

#### 3.4.2. Compounds Preparation

MOE was used to generate the coordinates of compounds, followed by the pH adjustment of ionization and protonation states within a specific range by the wash module. Subsequently, the minimization of the energies of compounds was achieved by the software MMFF94x force field.

#### 3.4.3. Molecular Docking Studies

Molecular docking was performed using specific software LeadIT from BioSolveIT, GmbH Germany [59]. Compounds were docked using the FlexX receptor utility of LeadIT. The active site of the receptor (9.0 Å) was used for the docking analysis, designed derivatives, and related ligands. Binding energies were determined from the complex of ligand and receptor conformations. The maximal docked poses were selected for analysis (LeadIT). Such poses having low binding free energy were considered strong showing promising interactions with the receptor. The ligand and protein complex containing low binding energy for interaction was evaluated, and three-dimensional interaction modes were envisaged using discovery studio and PyMOL [51,60].

#### 3.4.4. Molecular Dynamics Simulation

A molecular dynamics simulation was performed to analyze the stiffness, wide-ranging mobility, and sub-cellular functions of proteins. Based upon docking analysis, we carried out molecular dynamics simulation studies on the least binding energy value and highest docked pose complex. MD simulations were performed using online software, iMODS [61]. This software gives an appropriate association for this magnified normal mode analysis method within their coordinates. The web server is very unprompted and flexible to all important browsers and modern appliances. iMODs server also gives different parameters such as deformability of protein structure, B-factor values, computing the eigenvalues, variance, covariance map, and elastic network models. Keeping all the parameters by default, the PDB file of the docked complex was uploaded to an online server as an input file, and the results were observed within a few minutes.3.5. Anticancer Activity (SRB Dye Assay).

To investigate the cytotoxicity of potent compounds (**8a**, **8c**, **8g**, and **9i**), an SRB assay was performed against Vero cell lines (normal kidney epithelial cells). The total protein generated when cells were treated with putative anticancer drugs was assessed using a very precise calorimetric technique. A negative charge fluorescent dye, sulforhodamine B (SRB), a water-soluble luminous dye that electrostatically binds to proteins when the pH of the medium is significantly lowered, was applied. It can readily connect with cells that have been fixed with trichloroacetic acid (TCA) [62]. A bright color is produced by the SRB dye when it binds solely to intracellular proteins, and the color is proportional to the quantity of protein present in the cell.

## 4. Conclusions

In summary, a library of new piperidinyl-quinoline acylhydrazones was designed and synthesized using a hybridization strategy. The robust multistep synthetic route was established where the final step involves an ultrasound-assisted condensation of 6/8-methyl-2-(piperidin-1-yl)quinoline-3-carbaldehydes and (un)substituted aromatic acid hydrazides. The target compounds were obtained in 4–6 min in 80–97% yield. The estimation of inhibitory potential against cholinesterases exhibited the discovery of various potent and therapeutically effective inhibitors, specifically compound **8c** with an IC_50_ value of 5.3 ± 0.51 μM against AChE and compound **8g** and with an IC_50_ value of 1.31 ± 0.05 μM against BuChE. Compound **8g** was identified as a selective inhibitor of BuChE, whereas compounds **9h-j** were selective inhibitors of AChE. Compounds **8a-c** showed promising dual inhibition. The diverse structural variations on the hybrid scaffold offered ample opportunity to explore the in vitro results further and draw structural-activity relationships considering the important role of various substitutions on the inhibitory activity profile. Molecular docking and dynamics simulation data, as well as physicochemical properties results, also favored the development of piperidinyl-quinoline acylhydrazones hybrids as promising compounds for the treatment of Alzheimer’s disease.

## Figures and Tables

**Table 1:** Comparative yields (conventional vs. ultrasonic-assisted synthesis) of piperidinyl-quinoline *N*-acylhydrazones (**8a-m** and **9a-j**).

Compound	Substituent			Yield (%)	
	R^1^	R^2^	R^3^	Conventional Approach	Ultrasonic-Assisted Approach
**8a**	Me	H	H	78	86
**8b**	Me	H	3-NO_2_	75	82
**8c**	Me	H	4-NO_2_	71	80
**8d**	Me	H	2-NH_2_	93	97
**8e**	Me	H	4-NH_2_	87	94
**8f**	Me	H	2-Cl	74	83
**8g**	Me	H	3-Cl	70	81
**8h**	Me	H	4-Cl	79	87
**8i**	Me	H	2,4-Cl_2_	72	81
**8j**	Me	H	2-OMe	84	91
**8k**	Me	H	3-OMe	65	84
**8l**	Me	H	4-OMe	94	98
**8m**	Me	H	2-OH	77	85
**9a**	H	Me	H	77	85
**9b**	H	Me	3-NO_2_	71	80
**9c**	H	Me	4-NO_2_	70	81
**9d**	H	Me	2-Cl	73	82
**9e**	H	Me	3-Cl	75	83
**9f**	H	Me	4-Cl	80	89
**9g**	H	Me	2,4-Cl_2_	75	87
**9h**	H	Me	2-OMe	85	93
**9i**	H	Me	3-OMe	71	83
**9j**	H	Me	4-OMe	91	95

**Table 2:** Anti-cholinesterase (AChE and BuChE) potential of synthesized compounds **8a–m**.

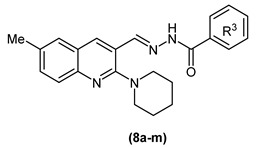			
Compound	Substituent (R^3^)	Acetylcholinesterase (AChE) Inhibition	Butyrylcholinesterase (BuChE) Inhibition
		IC_50_ ± SEM (μM)/%Inhibition	
**8a**	H	6.1 ± 0.31	4.64 ± 0.43
**8b**	3-NO_2_	6.3 ± 0.23	4.71 ± 0.45
**8c**	4-NO_2_	5.3 ± 0.51	1.74 ± 0.03
**8d**	2-NH_2_	35	21
**8e**	4-NH_2_	61.3 ± 0.71	23
**8f**	2-Cl	40	15
**8g**	3-Cl	32	1.31 ± 0.05
**8h**	4-Cl	44	34
**8i**	2,4-Cl_2_	41	39
**8j**	2-OMe	43	30
**8k**	3-OMe	42	28
**8l**	4-OMe	37	15
**8m**	2-OH	31	13
**Neostigmine**	—	16.3 ± 1.12	—
**Donepezil**	—	—	7.23 ± 0.12

**Table 3:** Anti-cholinesterase (AChE and BuChE) potential of synthesized compounds **9a-j**.

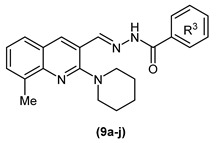			
Compound	Substituent (R^3^)	Acetylcholinesterase (AChE) Inhibition	Butyrylcholinesterase (BuChE) Inhibition
		IC_50_ ± SEM (μM)/%Inhibition	
**9a**	H	41	32
**9b**	3-NO_2_	35	27
**9c**	4-NO_2_	32	32
**9d**	2-Cl	36	38
**9e**	3-Cl	35	40
**9f**	4-Cl	33	36
**9g**	2,4-Cl_2_	27	32
**9h**	2-OMe	16.4 ± 0.09	33
**9i**	3-OMe	9.6 ± 0.02	27
**9j**	4-OMe	14.06 ± 0.06	38
**Neostigmine**	—	16.3 ± 1.12	—
**Donepezil**	—	—	7.23 ± 0.12

## Data Availability

The data presented in this study are available in supplementary material.

## Supplementary Materials

The following supporting information can be downloaded at: https://www.mdpi.com/article/10.3390/molecules28052131/s1, Table S1. Types of binding interactions, distance of bonds and atoms involved in interactions (AChE). Table S2. Types of binding interactions, distance of bonds and atoms involved in interactions (BuChE). Table S3. ADMET prediction scores for the potent compounds. ^1^H- and ^13^C-NMR spectra of all the synthesized compounds.

## Sample Availability

Samples of the synthetic compounds are available from the authors upon reasonable request.
