# Supplementary material for: Ultrasound-Assisted Synthesis of Piperidinyl-Quinoline Acylhydrazones as New Anti-Alzheimer’s Agents: Assessment of Cholinesterase Inhibitory Profile, Molecular Docking Analysis, and Drug-like Properties
Source: Molecules. 2023 Feb 24;28(5):2131. doi: 10.3390/molecules28052131 (PMC10004187; doi:10.3390/molecules28052131)

SUPPLEMENTARY INFORMATION

Ultrasound-Assisted Synthesis of Piperidinyl-Quinoline Acylhydrazones as New Anti-Alzheimer's Agents: Assessment of Cholinesterase Inhibitory Profile, Molecular Docking Analysis, and Drug-like Properties

Rubina Munir [1,2,*], Sumera Zaib [3,*], Muhammad Zia-ur-Rehman [4], Nadia Hussain [5,6], Faryal Chaudhry [2], Muhammad Tayyab Younas [3], Fatima Tuz Zahra [7], Zainab Tajammul [3], Noman Javid [8], Ayed A. Dera [9], Hanan A. Ogaly [10,11] and Imtiaz Khan [12,*]

1 School of Chemistry, University of the Punjab, Lahore 54590, Pakistan

2 Department of Chemistry, Kinnaird College for Women, Lahore 54000, Pakistan; faryal.chaudhry@kinnaird.edu.pk

3 Department of Basic and Applied Chemistry, Faculty of Science and Technology, University of Central Punjab, Lahore 54590, Pakistan; muhammadtayyabyounassst@gmail.com (M.T.Y.); zainabbakht97@gmail.com (Z.T.)

4 Applied Chemistry Research Centre, PCSIR Laboratories Complex, Lahore 54600, Pakistan; rehman_pcsir@hotmail.com

5 Department of Pharmaceutical Sciences, College of Pharmacy, Al Ain University, Al Ain P.O. Box 64141, United Arab Emirates; nadia.hussain@aau.ac.ae

6 AAU Health and Biomedical Research Center, Al Ain University, Abu Dhabi P.O. Box 144534, United Arab Emirates

7 Department of Chemistry, Quaid-i-Azam University, Islamabad 45320, Pakistan; fatimaz1425@gmail.com

8 Chemistry Department (C-Block), Forman Christian College, Ferozepur Road, Lahore 54600, Pakistan; noumanjavid@gmail.com

9 Department of Clinical Laboratory Sciences, College of Applied Medical Sciences, King Khalid University, Abha, Saudi Arabia; ayedd@kku.edu.sa

10 Chemistry Department, College of Science, King Khalid University, Abha 61421, Saudi Arabia; ohanan@kku.edu.sa

11 Biochemistry and Molecular Biology Department, Faculty of Veterinary Medicine, Cairo University, Giza 12211, Egypt

12 Department of Chemistry and Manchester Institute of Biotechnology, The University of Manchester, 131 Princess Street, Manchester M1 7DN, UK

* Correspondence: organist94@gmail.com (R.M.); sumera.zaib@ucp.edu.pk (S.Z.); kimtiaz@hotmail.co.uk (I.K.)

Table S1. Types of binding interactions, distance of bonds and atoms involved in interactions (AChE).

Compound	Binding interactions			
	**Ligand Atom**	**Receptor Atom**	**Interaction Type**	**Distance (Å)**
**8a**	O22	TRP86	H-bond	2.54
	Quinoline	TRP86	π-π stacked	4.92
	N19	TRP86	C-H bond	3.04
	C14	TRP86	π-sigma	3.64
	Benzohydrazide	TRP439	π-π stacked	4.89
	Benzohydrazide	TYR337	π-π stacked	4.15
	Benzohydrazide	MET443	π-sulfur	4.17
	Benzohydrazide	PRO446	π-alkyl	4.87
	C11	Val73	Alkyl	4.20
	C11	PRO88	Alkyl	3.89
**8c**	O22	ARG296	H-bond	2.21
	O31	GLN369	H-bond	2.81
	C11	HIS405	Alkyl	4.51
	C11	TRP532	Alkyl	4.04
	C17	ASN233	C-H bond	3.20
	Piperidine	PRO312	Alkyl	5.05
	Quinoline	GLY234	Amide-π stacked	4.53, 4.07
	Quinoline	PRO235	π-sigma	3.92
	Quinoline	PRO235	π-alkyl	5.36
	Quinoline	PRO410	π-alkyl	4.89
**9i**	O21	TRP86	H-bond	2.62
	C15	TRP86	π-sigma	3.94
	Piperidine	TRP86	π-alkyl	5.07
	Quinoline	TRP86	π-π stacked	5.26
	Quinoline	SER125	π-donor H-bond	2.99
	Benzohydrazide	MET443	π-sulfur	4.40
	Benzohydrazide	TRP439	π-π stacked	3.58, 3.64
	Benzohydrazide	PRO446	π-alkyl	5.18
	C30	TYR337	Alkyl	4.28
	C30	TYR341	Alkyl	5.22
	C30	TRP439	Alkyl	3.92
	C30	MET443	Alkyl	5.37
	C30	VAL340	Alkyl	4.19
**Huprine W**	O2	SER203	H-bond	2.47
	Quinoline	TRP86	π-π stacked, π-alkyl	4.72, 4.34, 4.70, 4.40
	Quinoline	TYR337	π-π stacked	3.62, 4.81
	Cl1	TRP439	Alkyl	4.09
	Cl1	MET443	Alkyl	4.92
	Cl1	PRO446	Alkyl	3.99
	Cl1	TYR447	Alkyl	4.89
	Cl1	TYR337	Alkyl	4.85

Table S2. Types of binding interactions, distance of bonds and atoms involved in interactions (BuChE).

Compound	Binding interactions			
	**Ligand Atom**	**Receptor Atom**	**Interaction Type**	**Distance (Å)**
**8a**	H	PRO285	H-bond	1.93
	H	PRO285	C-H bond	3.08
	Piperidine ring	TYR332	π-alkyl	4.07
	Quinoline ring	ALA277	π-alkyl	4.87
	C11	ALA277	Alkyl	3.61
	O22	GLY117	C-H bond	2.25
	Benzohydrazide	TRP231	π-π T-shaped	6.16
	Benzohydrazide	PHE329	π-π T-shaped	5.25
	Benzohydrazide	GLY116	Amide-π Stacked	5.20
	Benzohydrazide	LEU286	π-Alkyl	5.06
**8c**	O30	TRP82	H-bond	3.12
	O30	TRP430	H-bond	2.82
	O30	TYR440328	H-bond	2.73
	C13	PRO285	C-H bond	3.34
	C17	LEU286	C-H bond	3.30
	C17	SER287	C-H bond	3.41
	Quinoline	GLY116	Amide-π stacked	4.75
	Aromatic ring	ALA328	π-alkyl	4.36
	Piperidine	TRP231	Alkyl	6.90
	Piperidine	LEU286	Alkyl	4.66
**8g**	N7	SER198	H-bond	3.38
	Piperidine	TRP82	π-sigma	3.43
	Piperidine	TRP82	π-alkyl	4.73
	Piperidine	HIS438	π-alkyl	4.92
	C17	SER198	C-H bond	3.43
	Quinoline	PHE329	π-π T-shaped	5.00
	Quinoline	GLY116	Amide-π stacked	4.35
	Quinoline	LEU286	π-alkyl	5.02
	C11	LEU286	Alkyl	4.05
	C11	VAL288	Alkyl	5.01
	C11	TRP231	π-sigma	3.45
	O22	GLY116	C-H bond	3.46
	C18	THR120	C-H bond	3.72
	Cl29	PRO84	Alkyl	3.47
	Benzohydrazide	PRO84	π-alkyl	5.17
**Tacrine**	N	SER198	H-bond	2.80
	Quinoline	GLY116	Amide-π stacked	4.13
	Quinoline	GLY116	Amide-π stacked	4.37
	Quinoline	PHE329	π-π T-Shaped	5.62
	Piperidine	PHE329	π-alkyl	5.48
	Piperidine	LEU286	π-alkyl	4.77
	Piperidine	TRP231	π-sigma	4.53, 3.57

Table S3. ADMET prediction scores for the potent compounds.

**Properties**	**8a**	**8c**	**8g**	**9i**
**Physicochemical Properties**				
Formula	$C_{23}H_{24}N_4O$	$C_{23}H_{23}N_5O_3$	$C_{23}H_{23}ClN_4O$	$C_{24}H_{26}N_4O_2$
Molecular weight (g/mol)	372.46	405.45	406.91	402.49
Number heavy atoms	28	30	29	30
Number of aromatic heavy atoms	16	16	16	16
Fraction C(sp^3)	0.26	0.23	0.26	0.29
Number of rotatable bonds	5	8	5	6
Number of H-bond acceptors	3	5	3	4
Number of H-bond donors	1	1	1	1
Molar refractivity	117.33	120.52	122.34	123.82
TPSA (Å²)	57.59	103.41	57.59	66.82
**Lipophilicity**				
Log $P_{o/w}$ (iLOGP)	3.68	3.45	3.60	3.48
Log $P_{o/w}$ (XLOGP3)	4.77	4.64	5.39	4.74
Log $P_{o/w}$ (WLOGP)	3.92	4.06	4.57	3.93
Log $P_{o/w}$ (MLOGP)	3.65	2.51	4.13	3.31
Log $P_{o/w}$ (SILICOS-IT)	4.54	2.29	5.17	4.59
Consensus Log $P_{o/w}$	4.11	3.39	4.57	4.01
**Water Solubility**				
Log S (ESOL)	-3.67	-5.14	-5.84	-5.32
Solubility	2.11e-03; 5.66e-06	2.91e-03 mg/ml; 7.18e-06 mol/l	5.93e-04; 1.46e-06	1.93e-03 mg/ml; 4.78e-06 mol/l
Class	Moderately soluble	Moderately soluble	Moderately soluble	Moderately soluble
Log S (Ali)	-5.71	-6.54	-6.35	-5.87
Solubility	7.26e-04; 1.95e-06	1.18e-04 mg/ml; 2.90e-07 mol/l	1.80e-04; 4.43e-07	5.39e-04 mg/ml; 1.34e-06 mol/l
Class	Moderately soluble	poorly soluble	poorly soluble	moderately soluble
Log S (SILICOS-IT)	-7.37	-6.92	-7.96	-7.47
Solubility	1.60e-05; 4.30e-08	4.92e-05 mg/ml; 1.21e-07 mol/l	4.51e-06; 1.11e-08	1.36e-05 mg/ml; 3.39e-08 mol/l
Class	Poorly soluble	Poorly soluble	Poorly soluble	Poorly soluble
**Pharmacokinetics**				
GI absorption	High	High	High	High
BBB permeant	Yes	No	Yes	Yes
P-gp substrate	No	No	No	No
CYP1A2 inhibitor	Yes	No	No	No
CYP2C19 inhibitor	Yes	Yes	Yes	Yes
CYP2C9 inhibitor	Yes	Yes	Yes	Yes
CYP2D6 inhibitor	No	No	No	Yes
CYP3A4 inhibitor	Yes	Yes	Yes	Yes
Log K_p (skin permeation)	-5.19 cm/s	-5.48 cm/s	-4.96	-5.39 cm/s
**Druglikeness**				
Lipinski	Yes; 0 violation	Yes; 0 violation	Yes; 0 violation	Yes; 0 violation
Ghose	Yes	Yes	Yes	Yes

Veber	Yes	Yes	Yes	Yes
Egan	Yes	Yes	Yes	Yes
Muegge	Yes	Yes	No; 1 violation: XLOGP3>5	Yes
Bioavailability score	0.55	0.55	0.55	0.55
**Medicinal Chemistry**				
PAINS	0 alert	0 alert	0 alert	0 alert
Brenk	1 alert: imine 1	3 alerts: imine 1, nitro group, oxygen-nitrogen single bond	1 alert: imine 1	1 alert: imine 1
Leadlikeness	No; 2 violations: MW>350, XLOGP3>3.5	No; 3 violations: MW>350, Rotors>7, XLOGP3>3.5	No; 2 violations: MW>350, XLOGP3>3.5	No; 2 violations: MW>350, XLOGP3>3.5
Synthetic accessibility	3.04	3.25	3.07	3.23

NMR Spectra of 8a

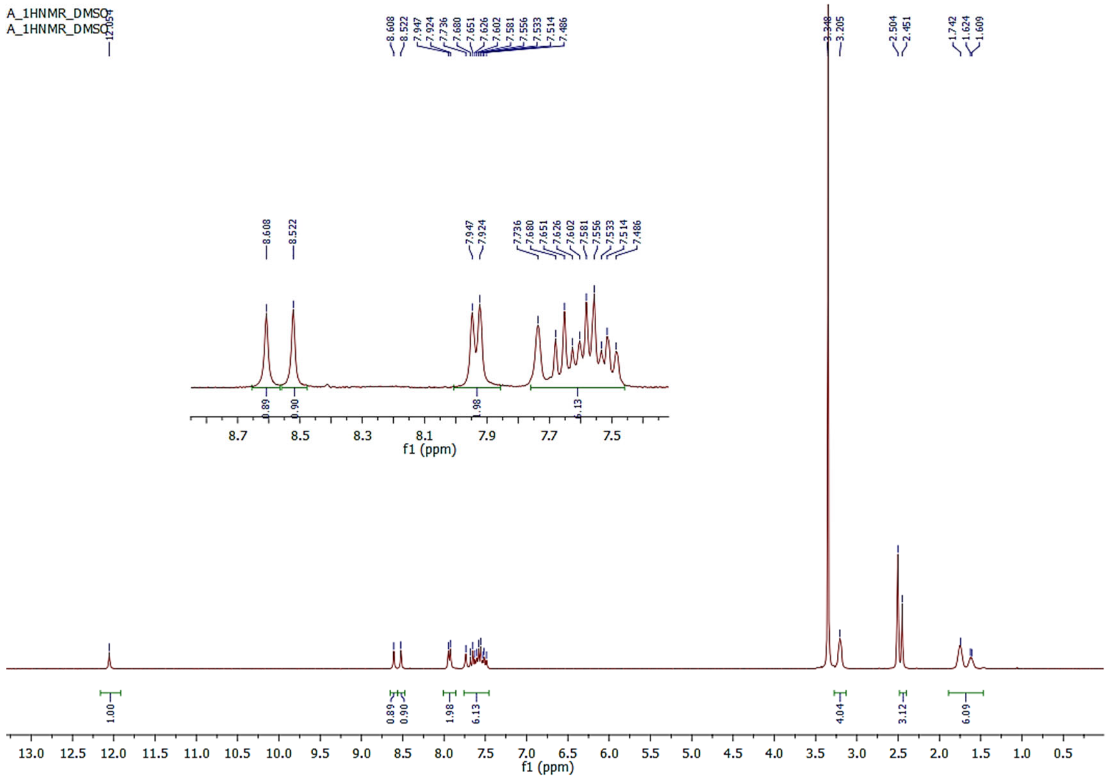

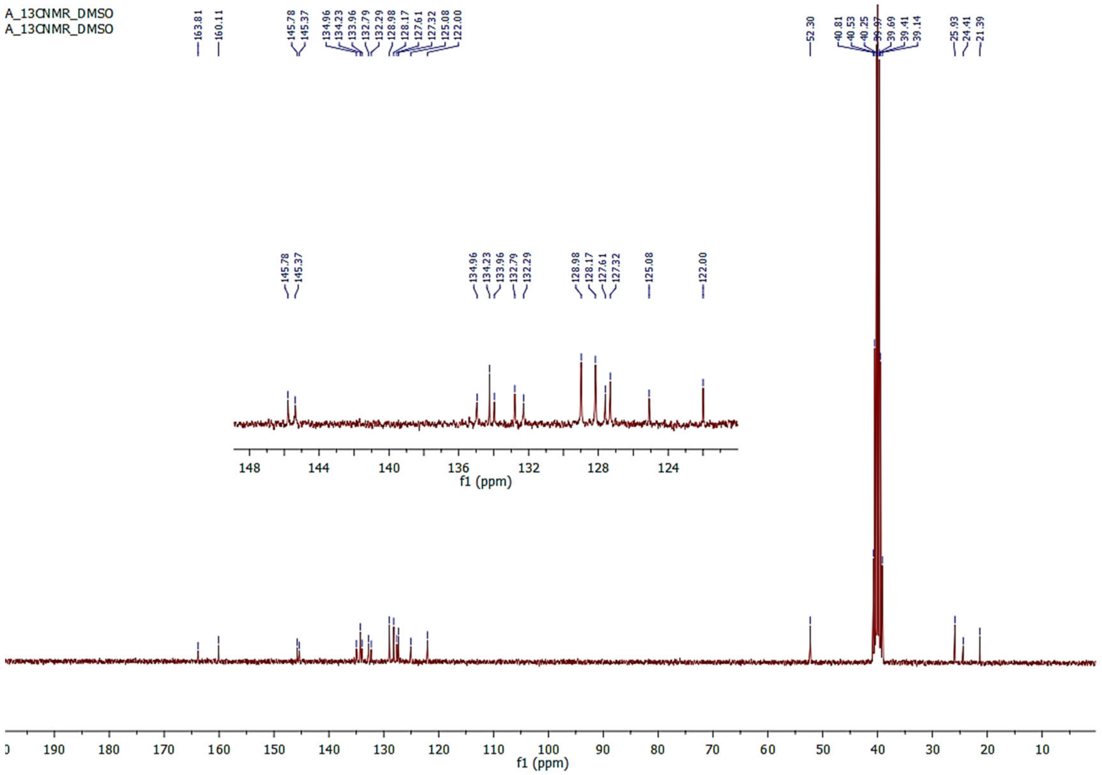

Spectra of 8d

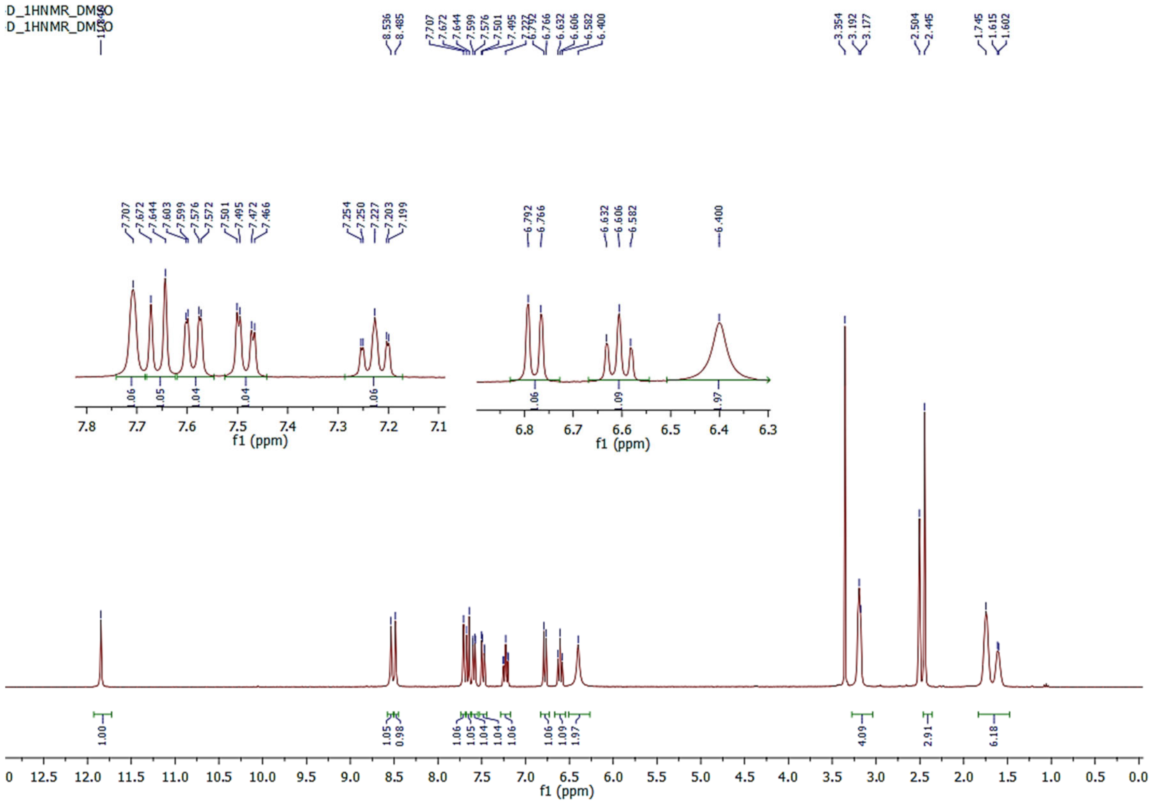

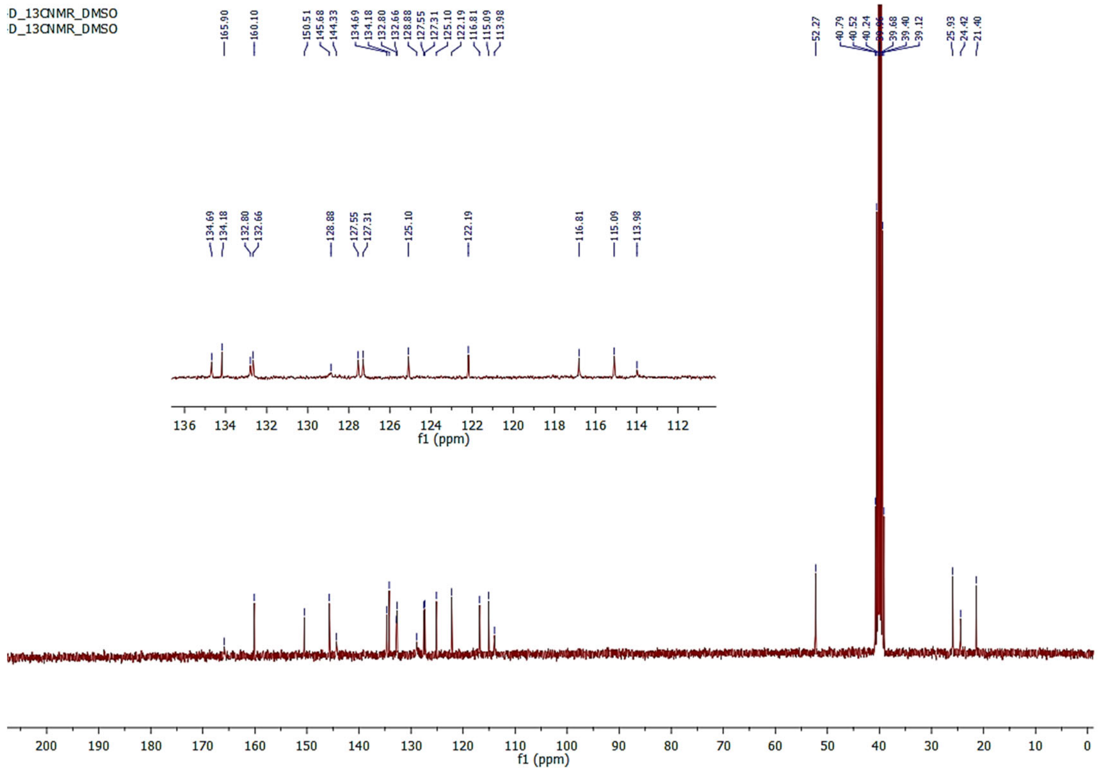

Spectra of 8e

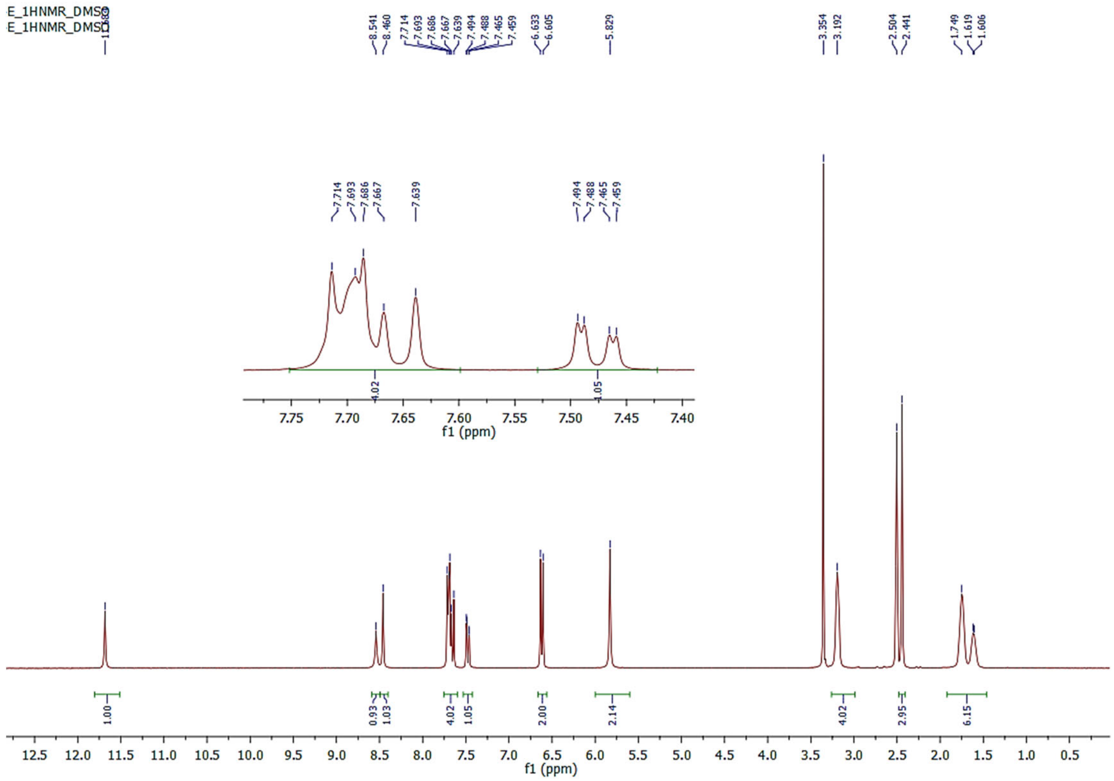

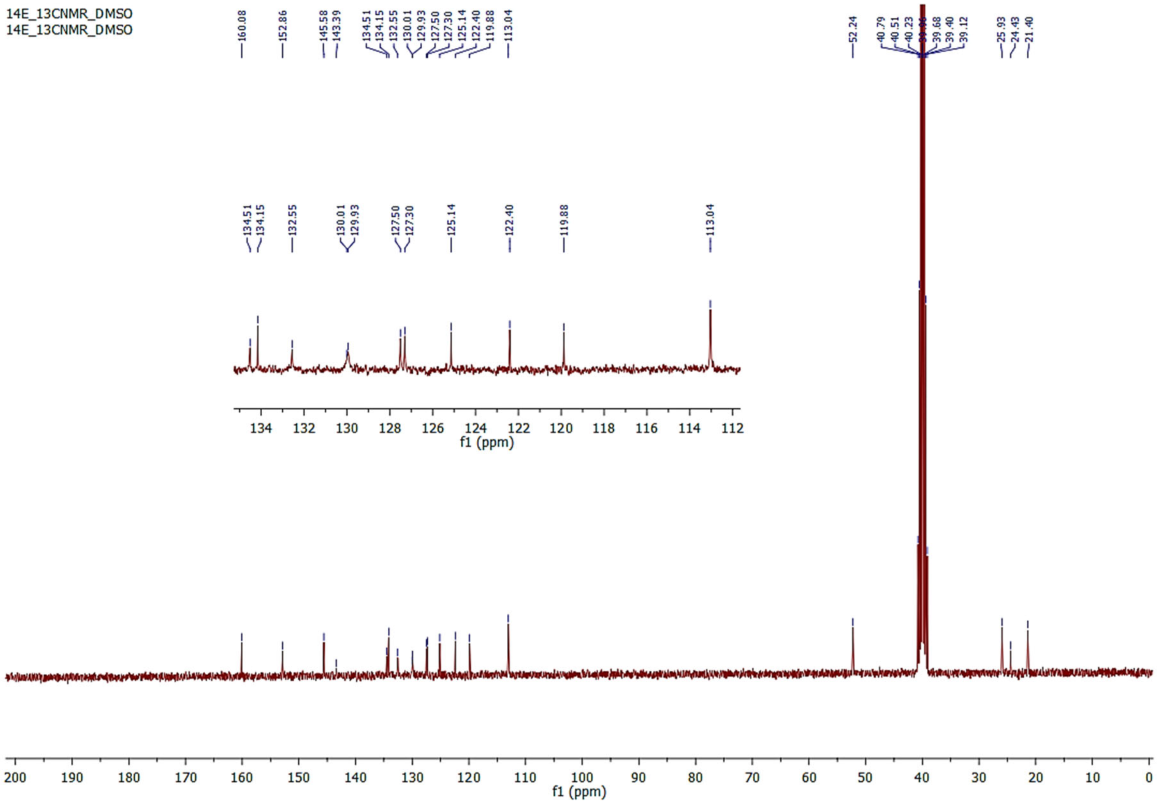

Spectra of 8f

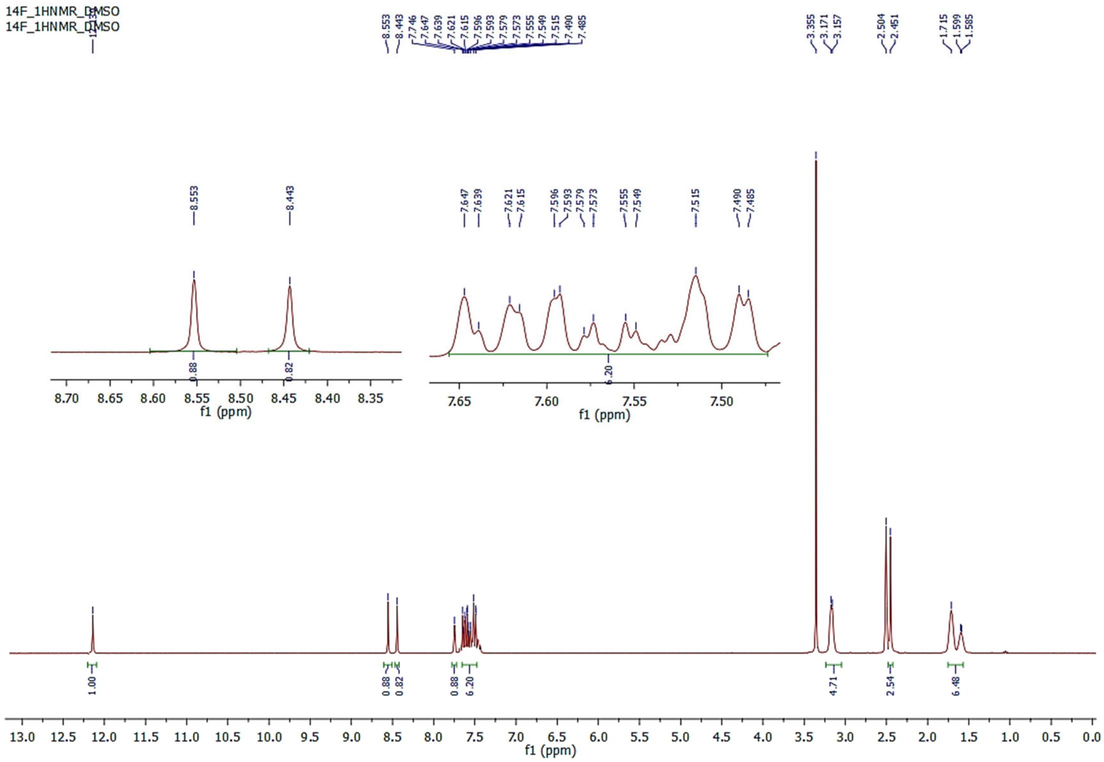

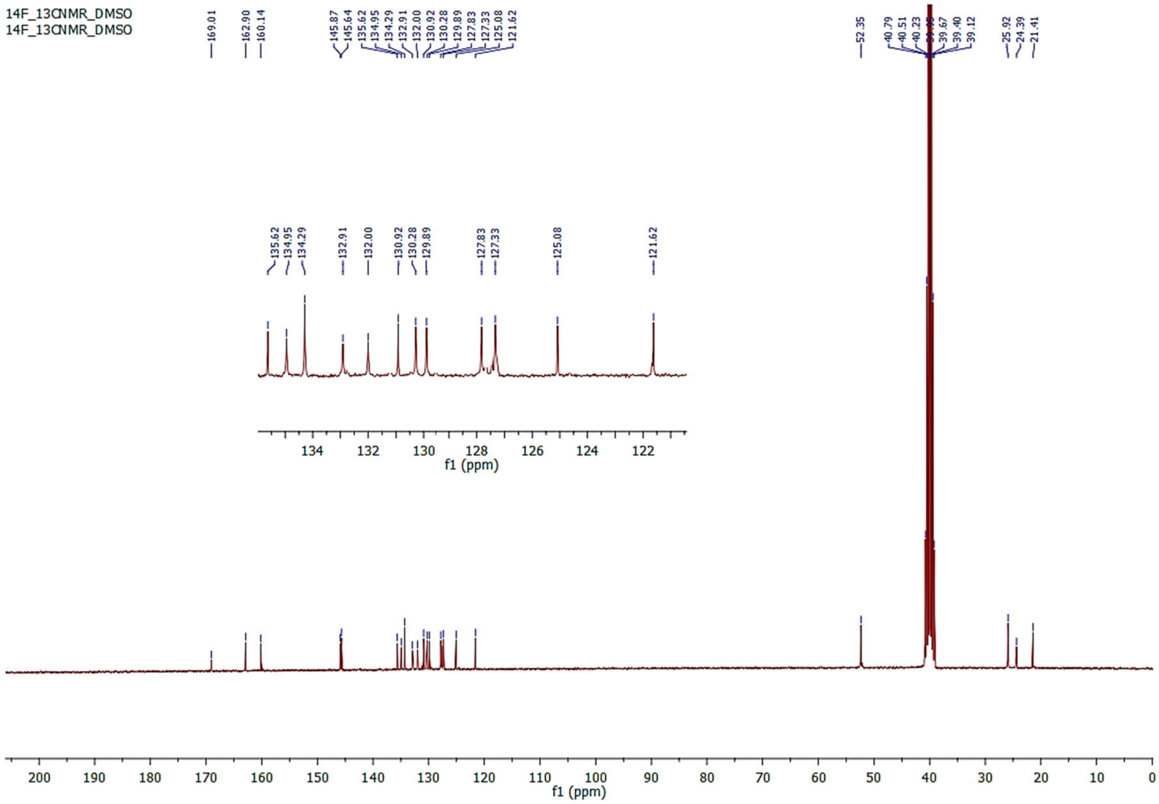

Spectra of 8g

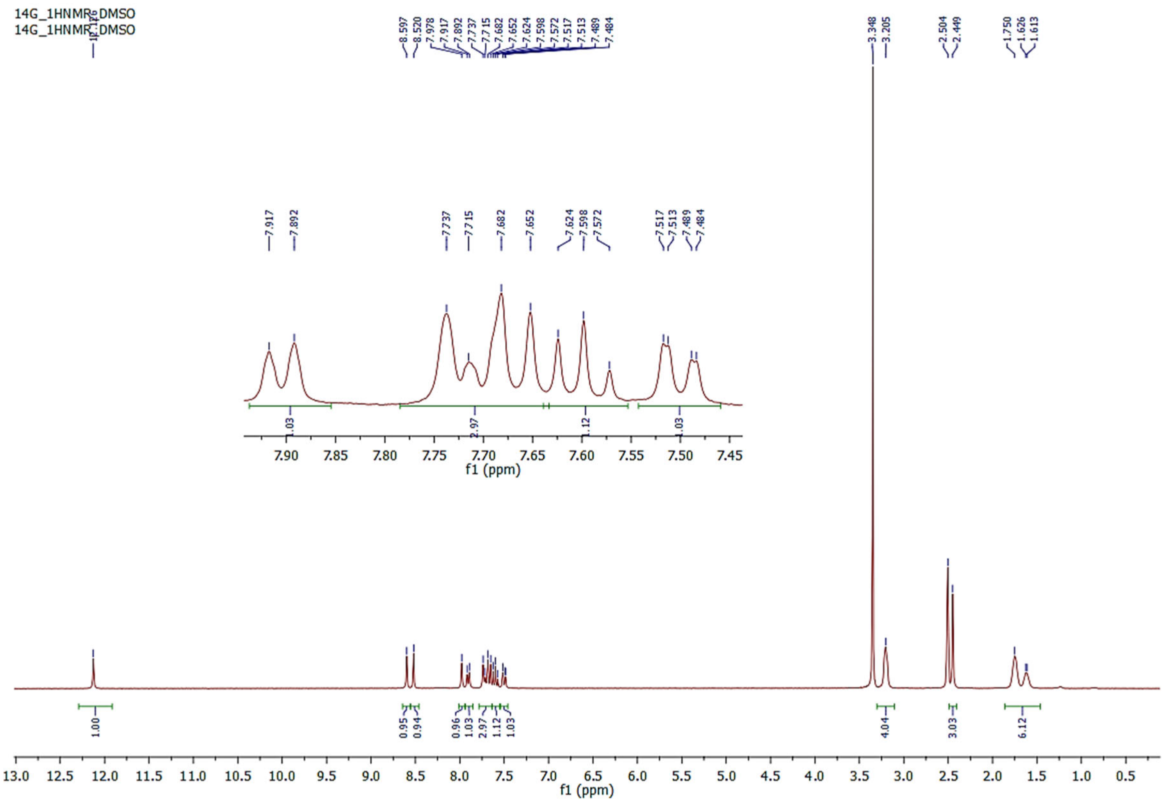

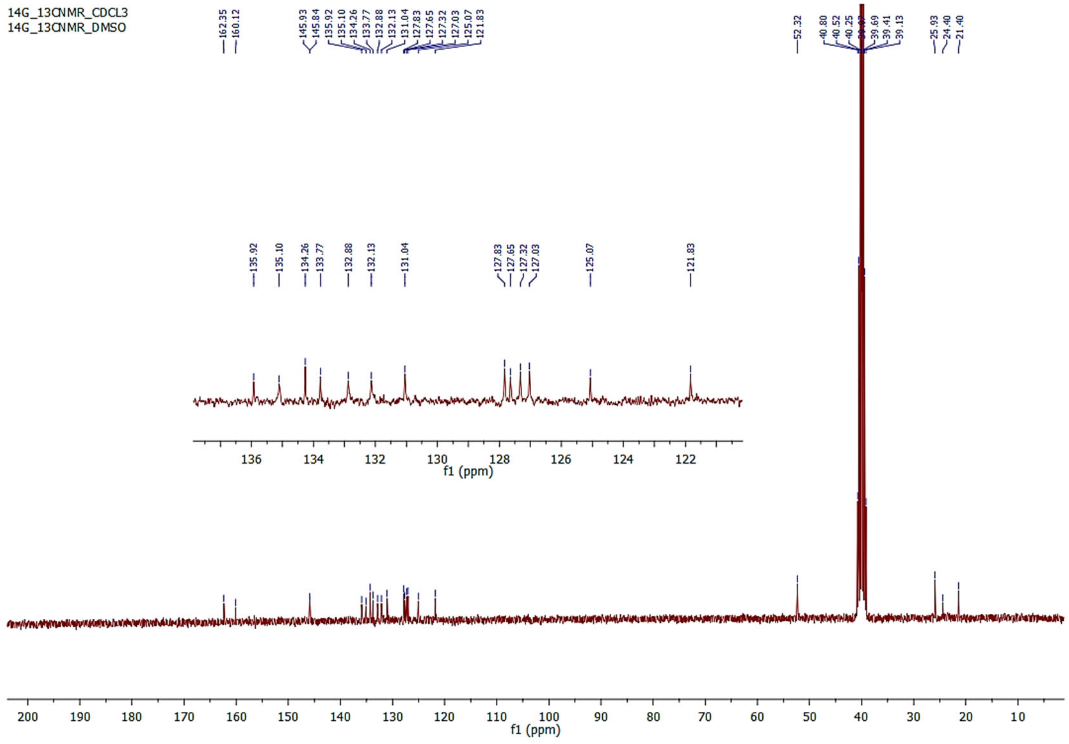

Spectra of 8h

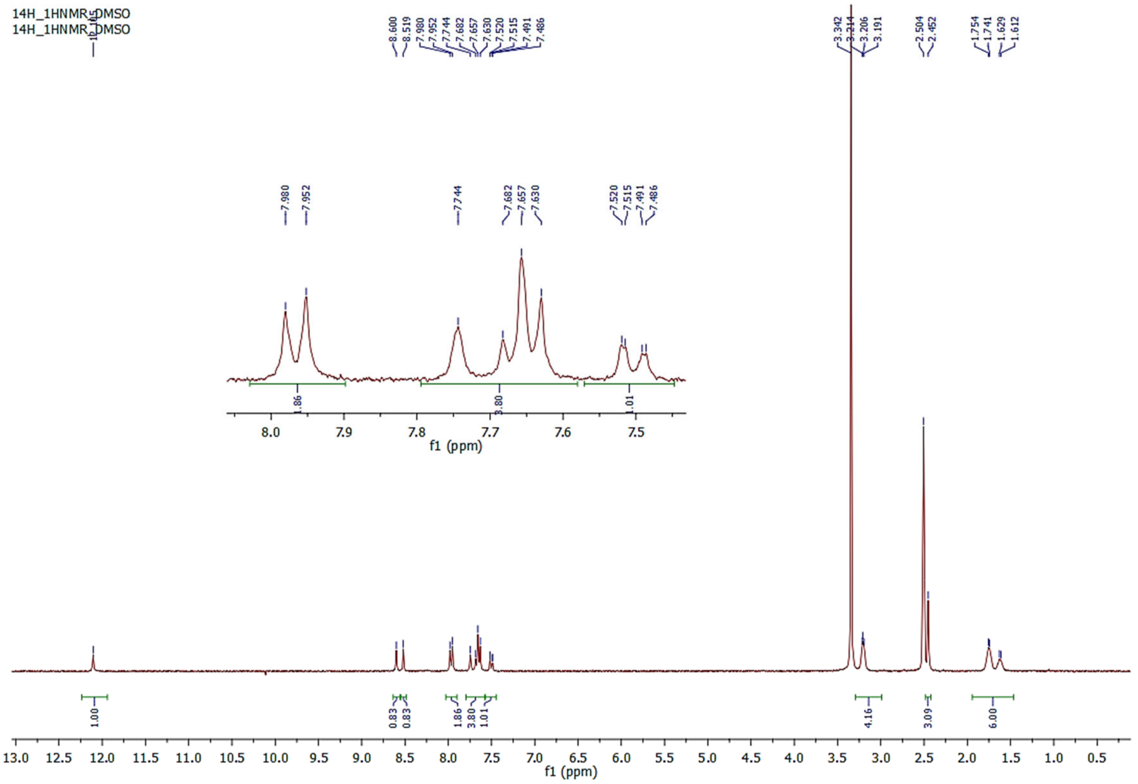

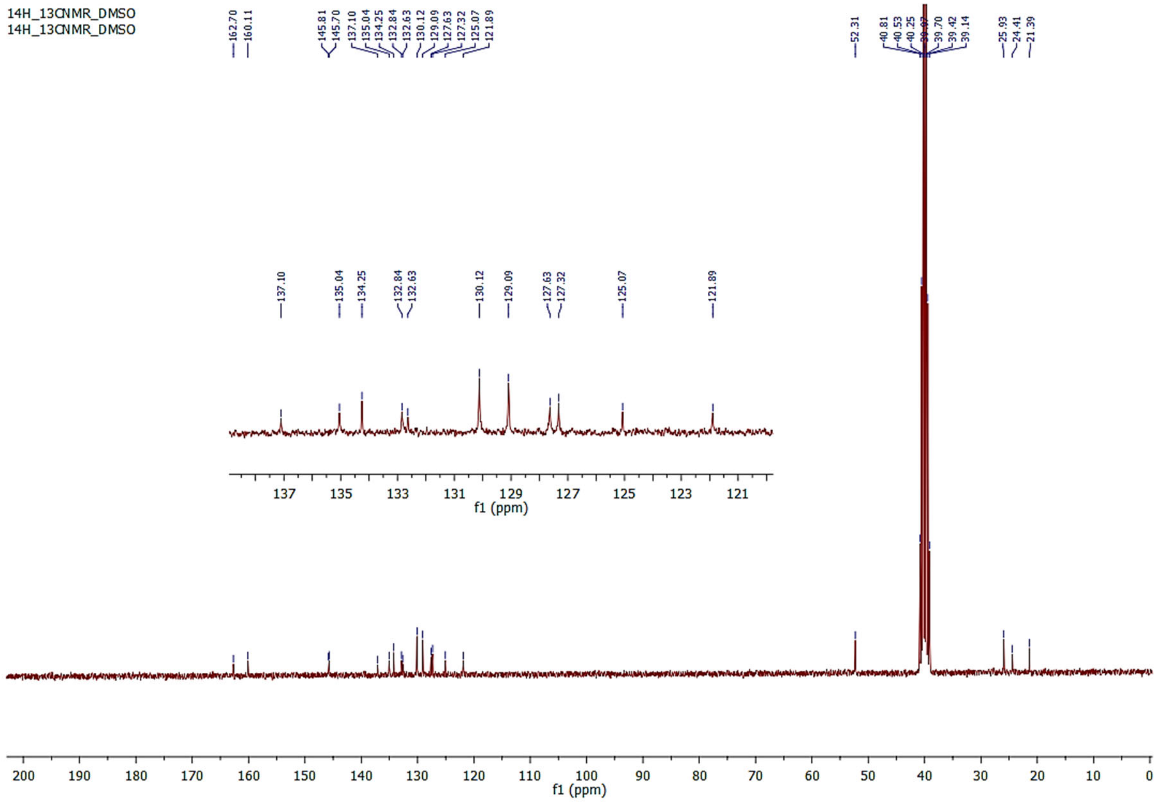

Spectra of 8i

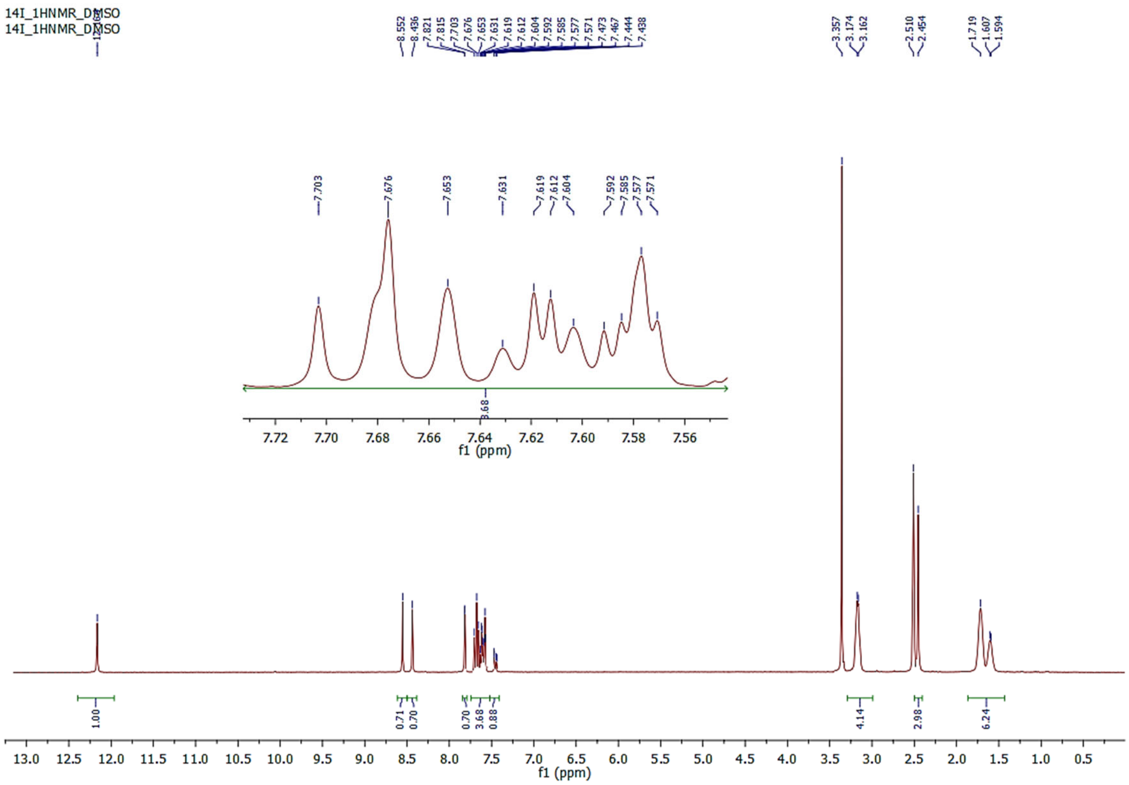

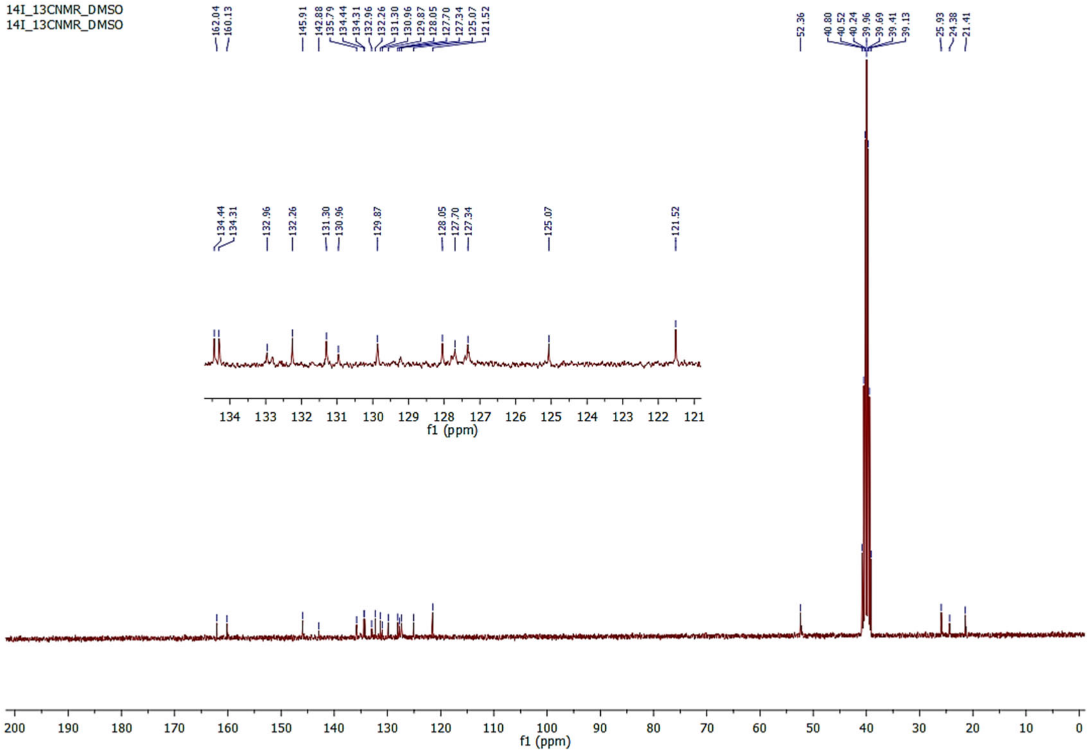

Spectra of 8j

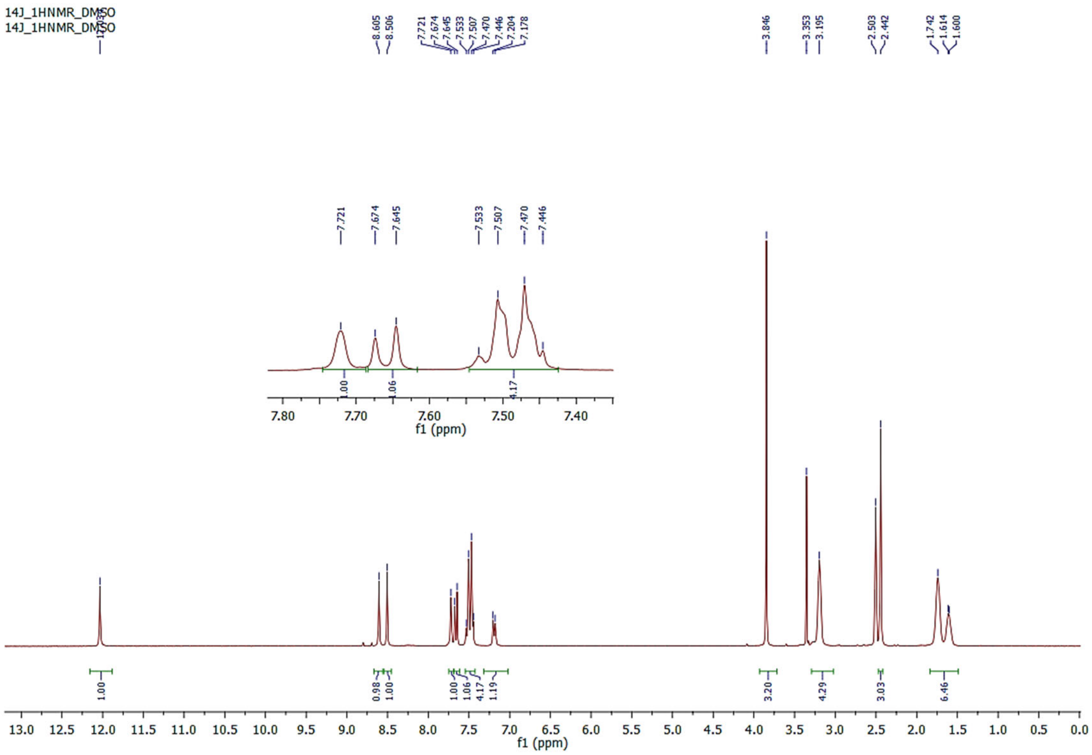

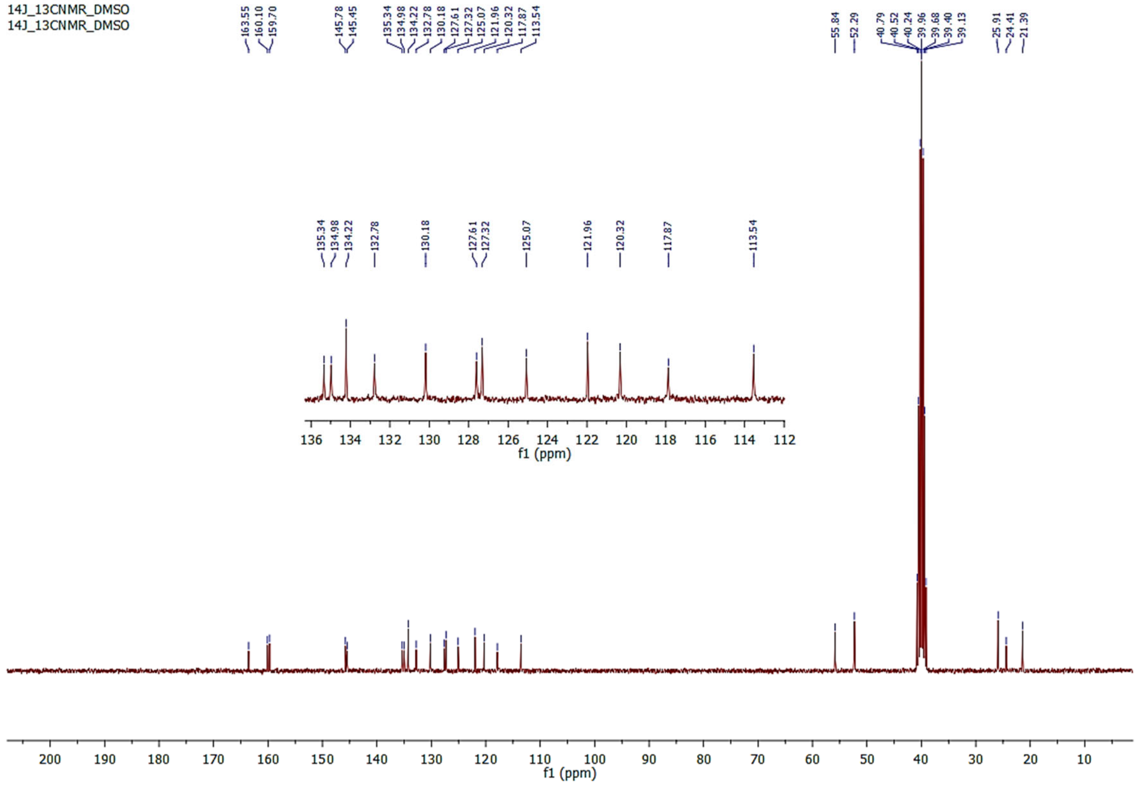

Spectra of 8k

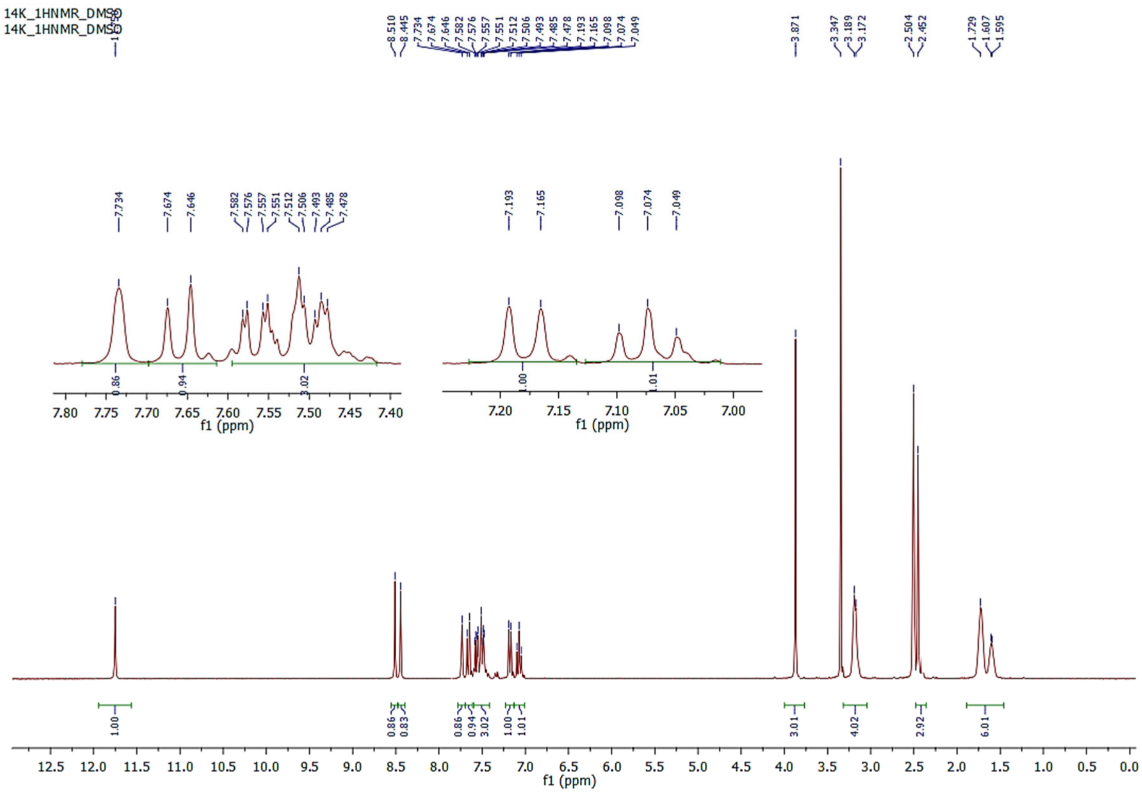

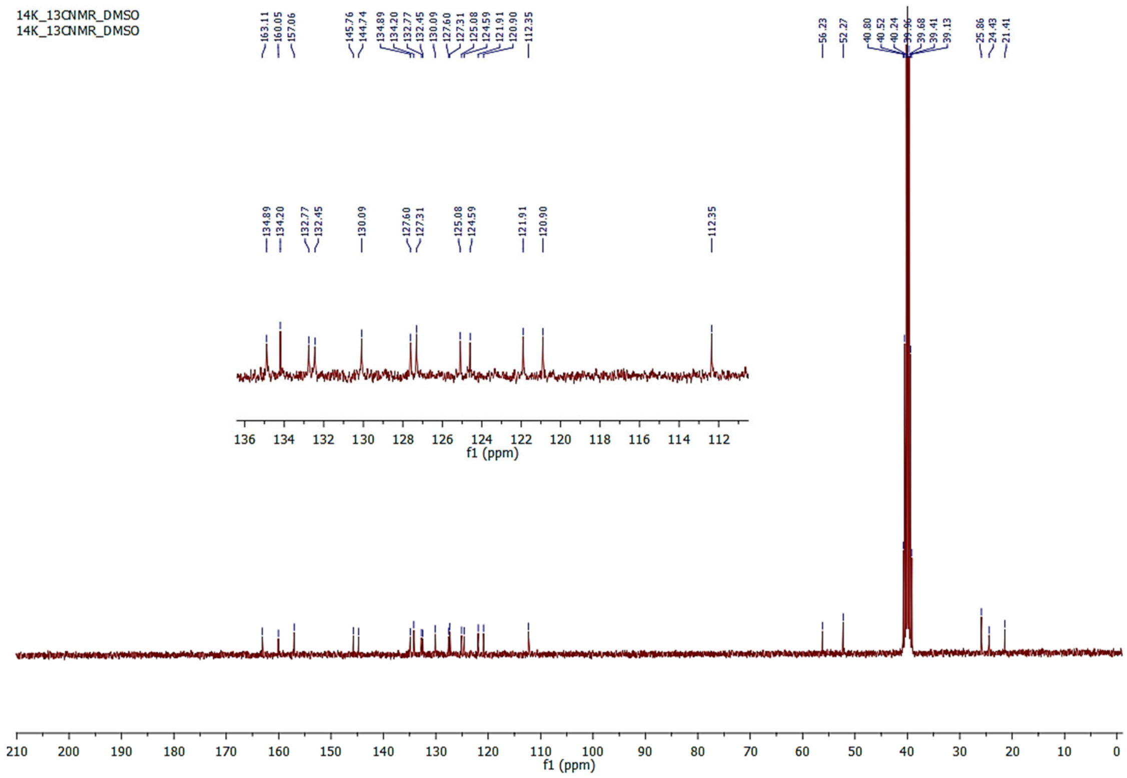

Spectra of 8l

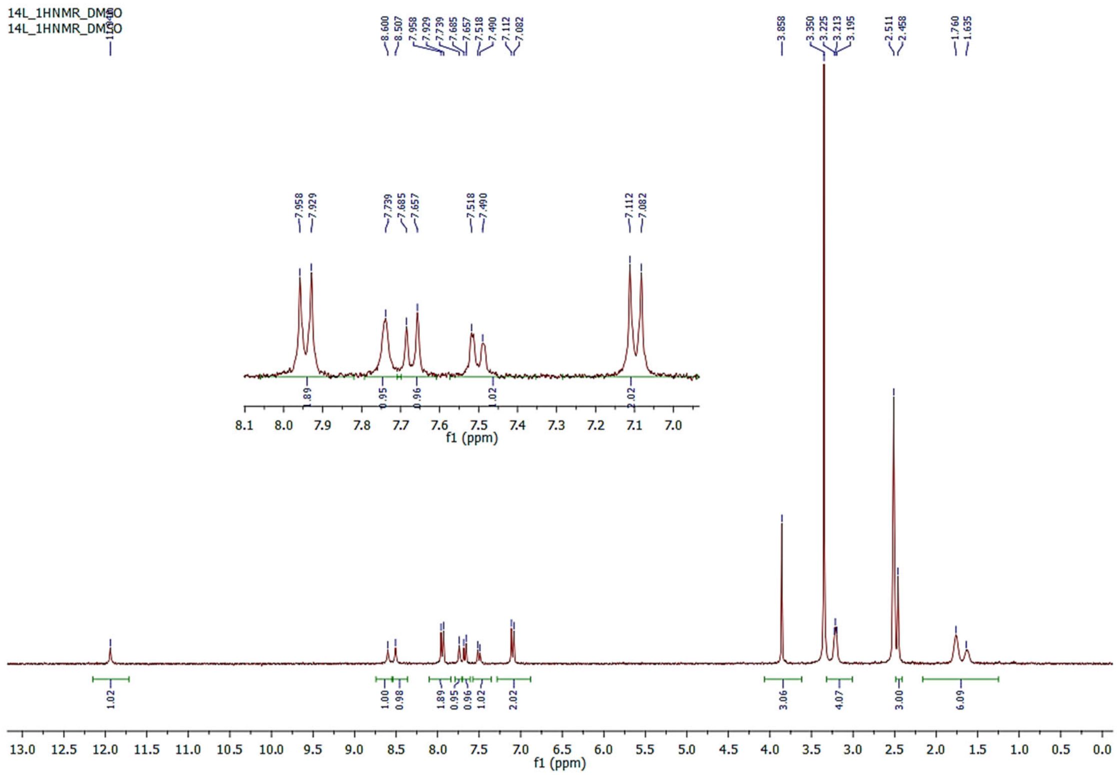

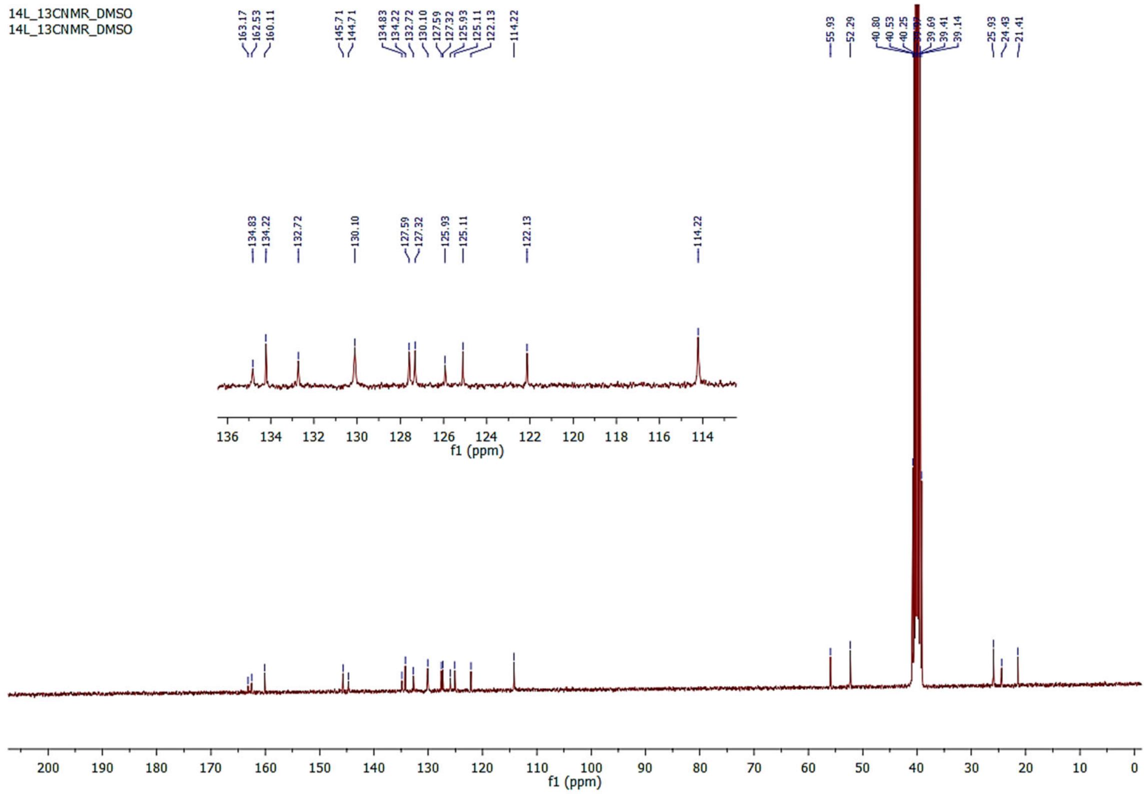

Spectra of 8m

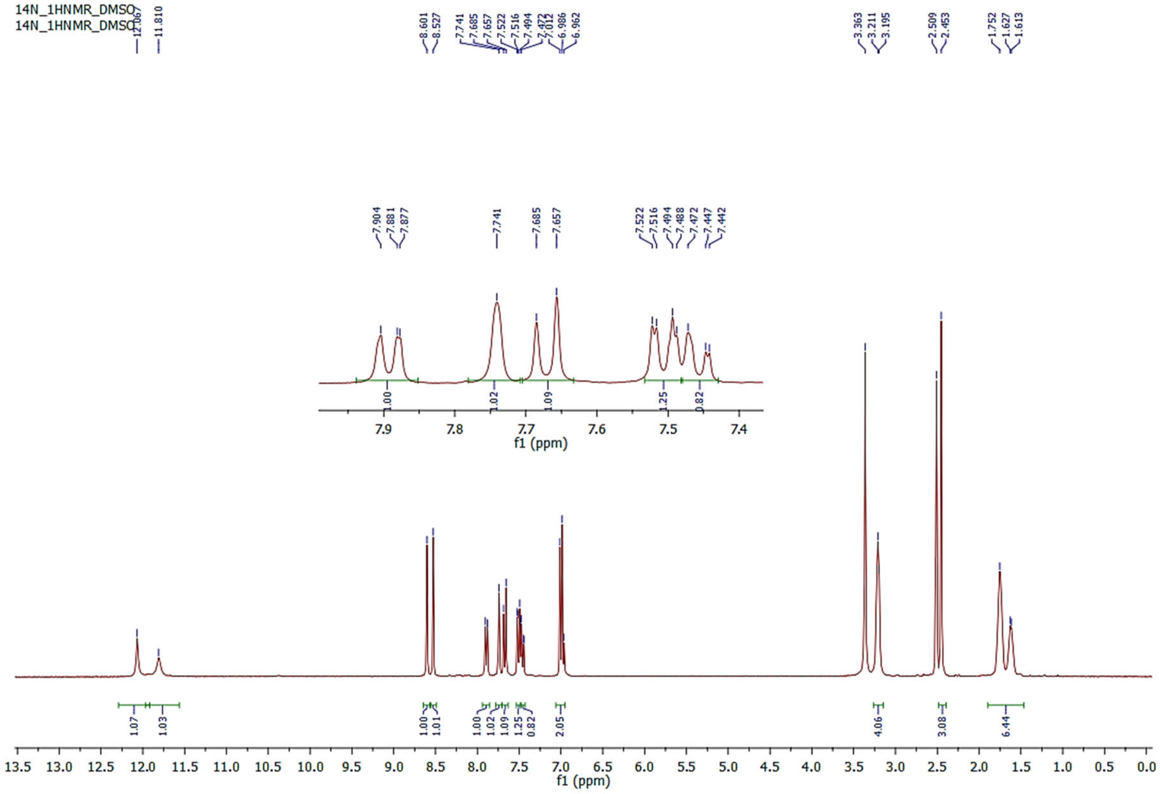

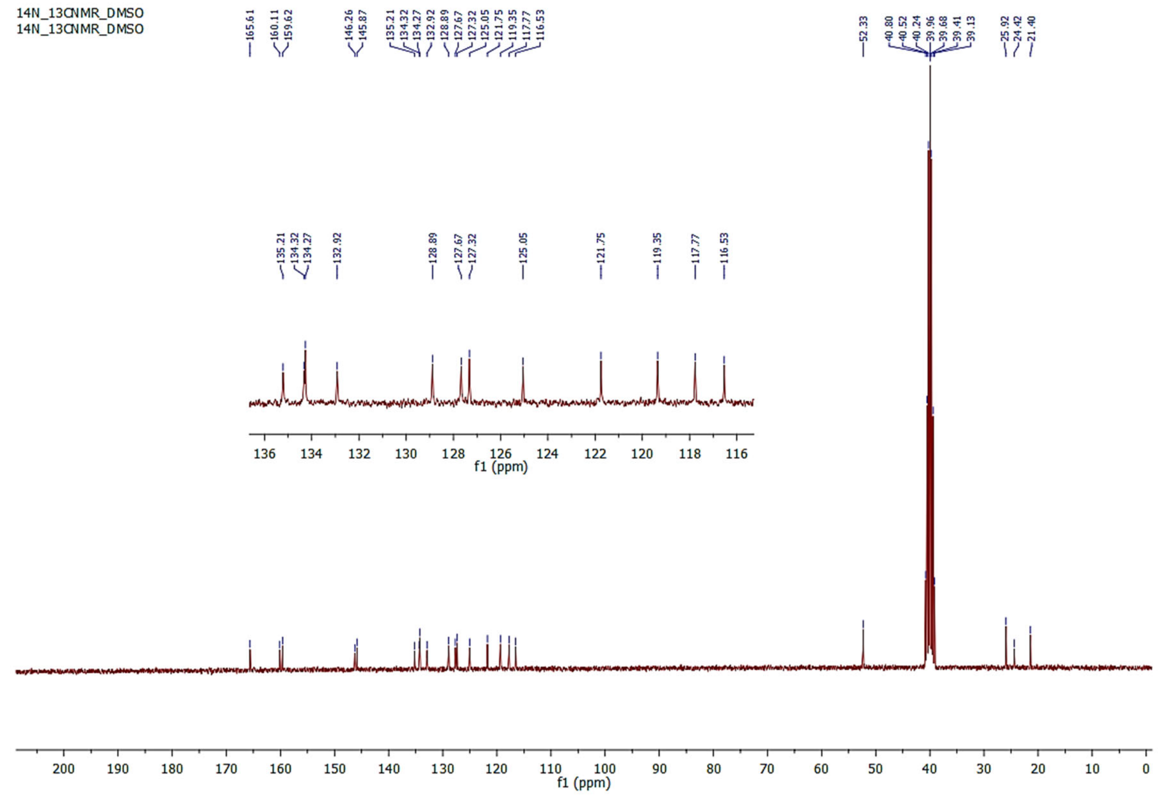

Spectra of 9a

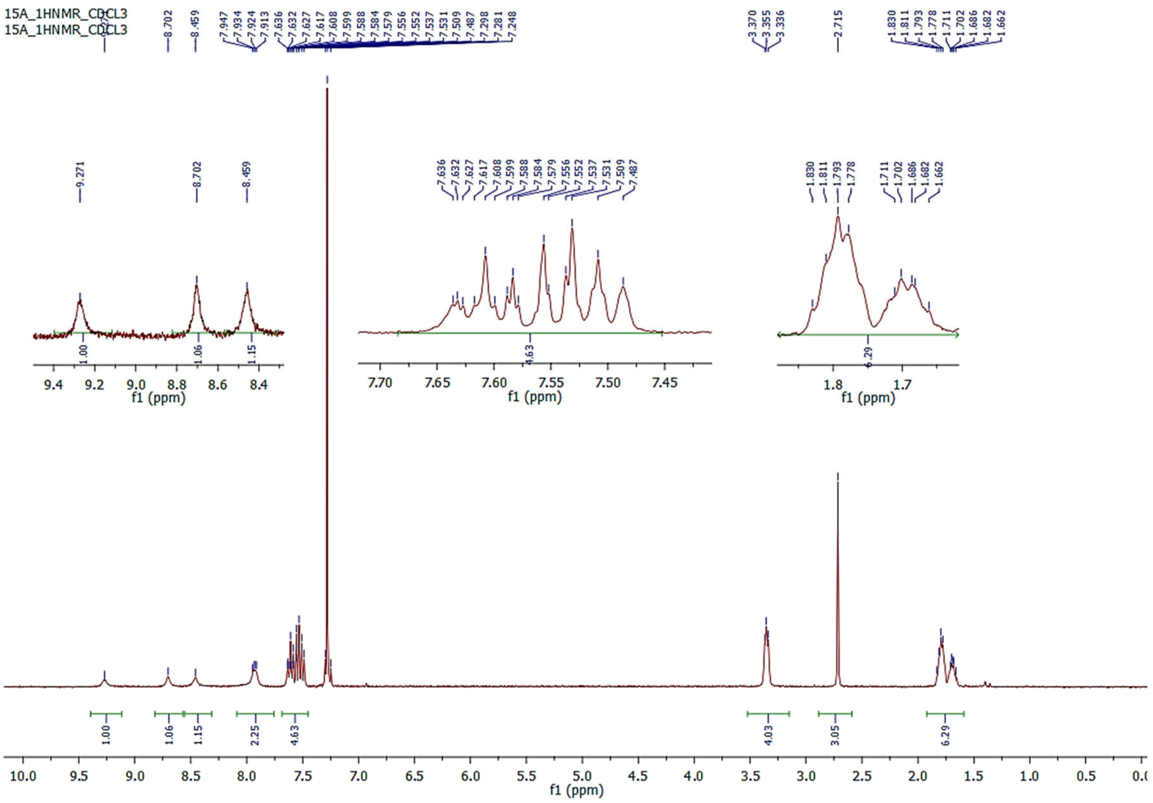

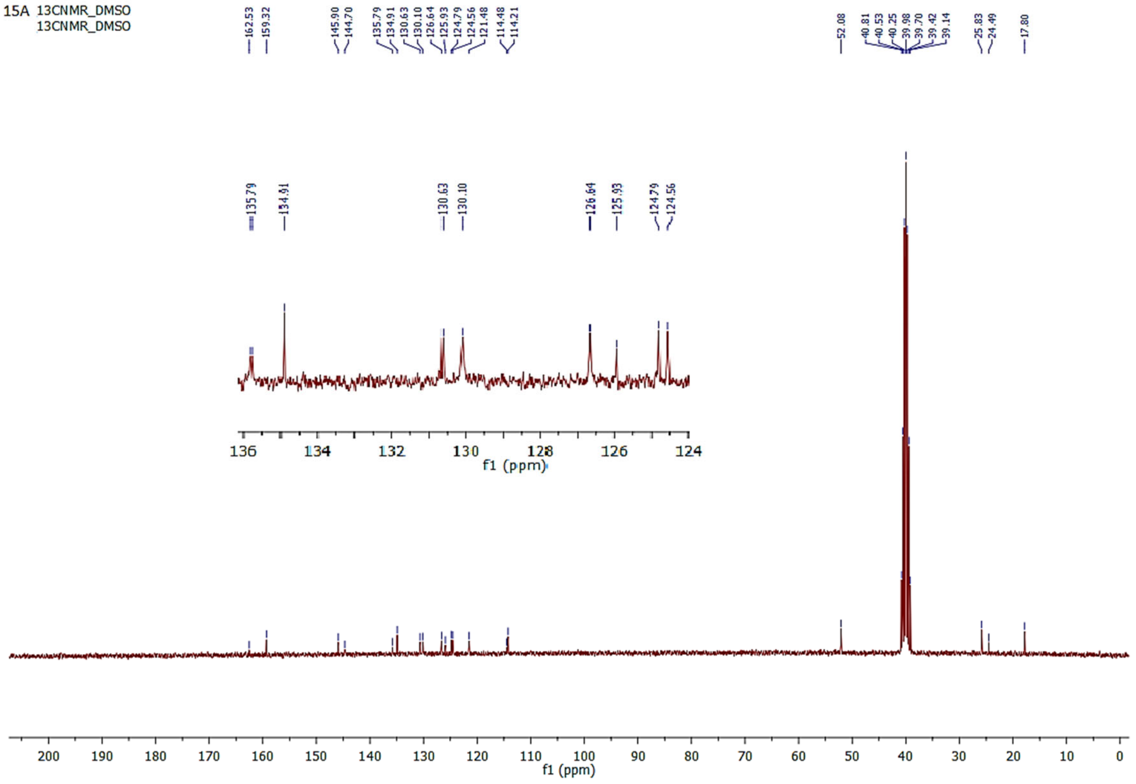

Spectra of 9b

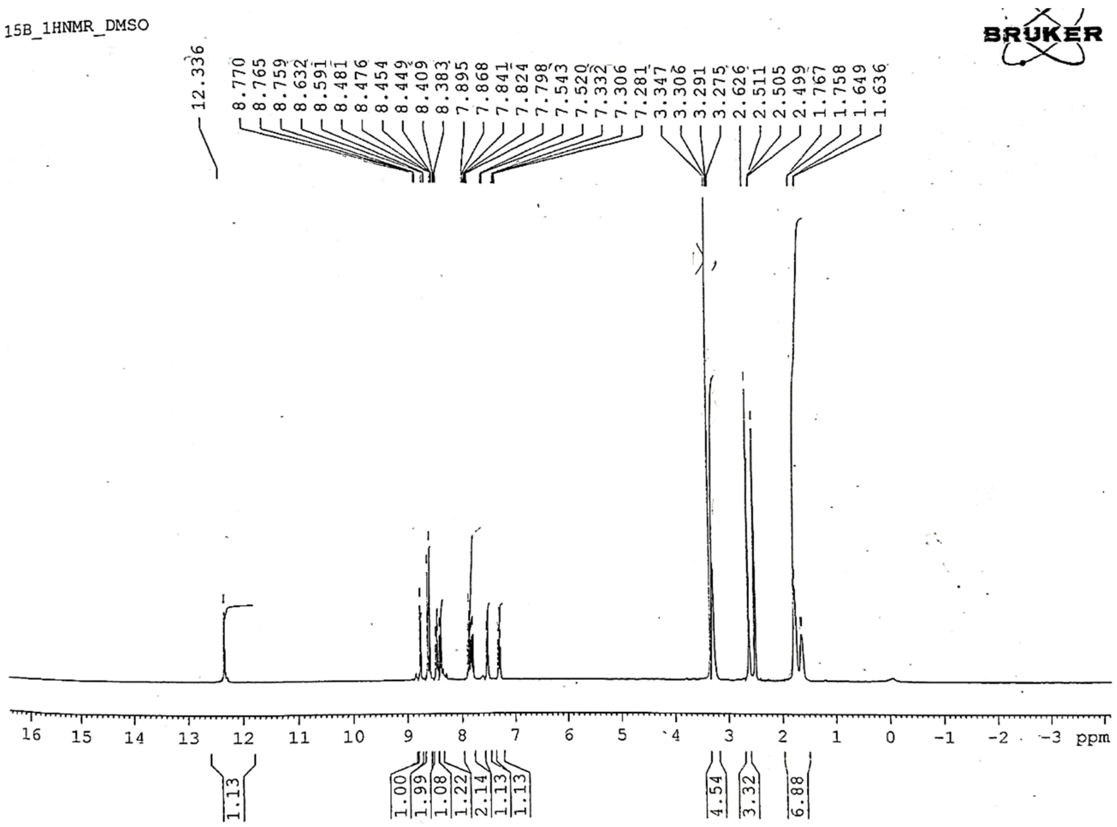

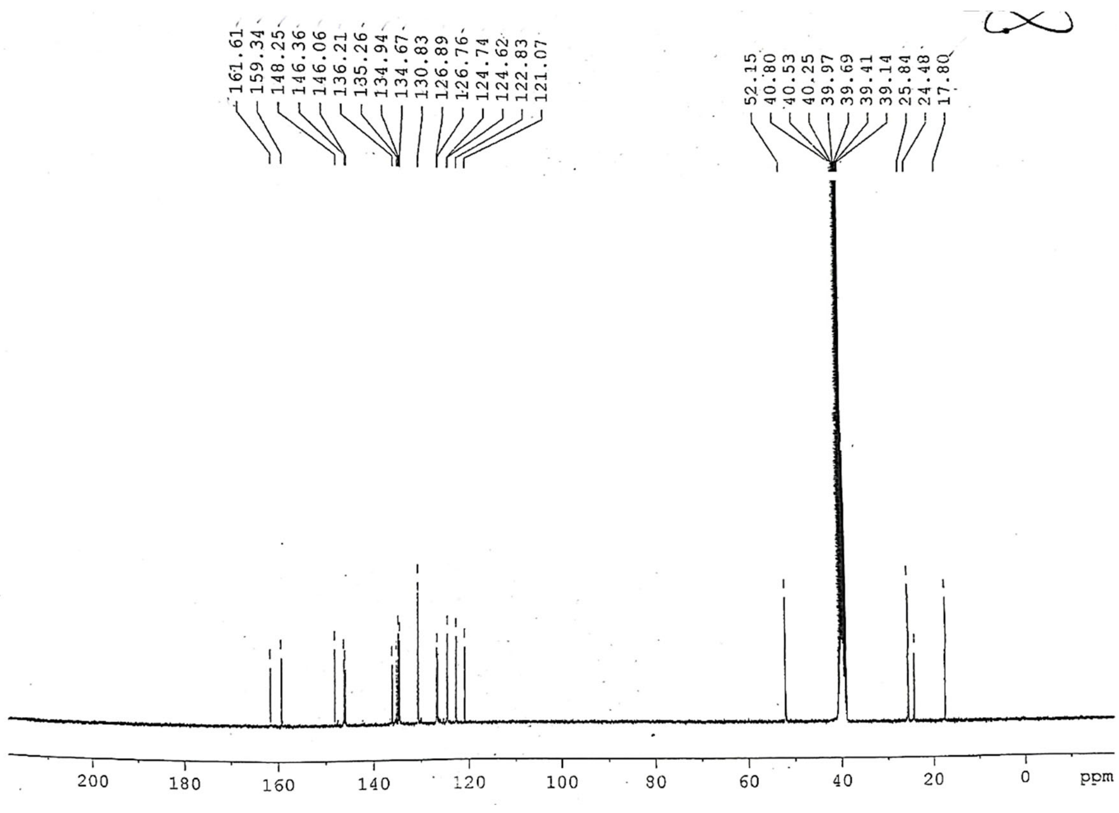

Spectra of 9d

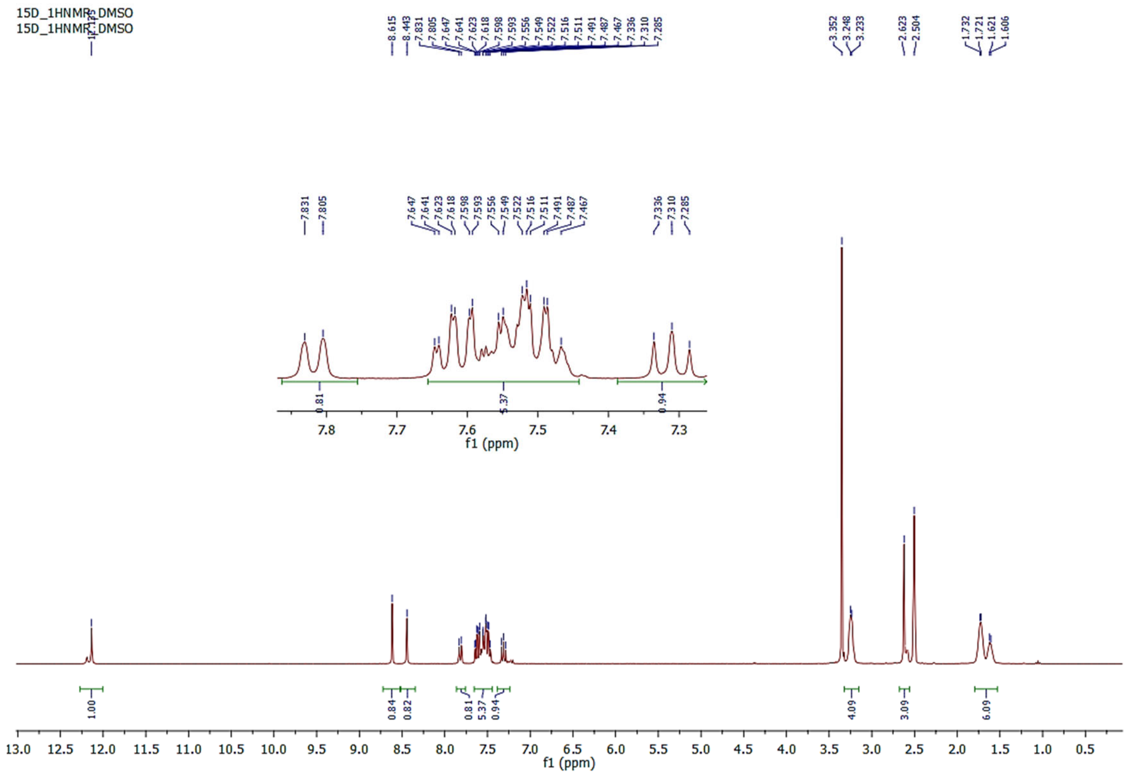

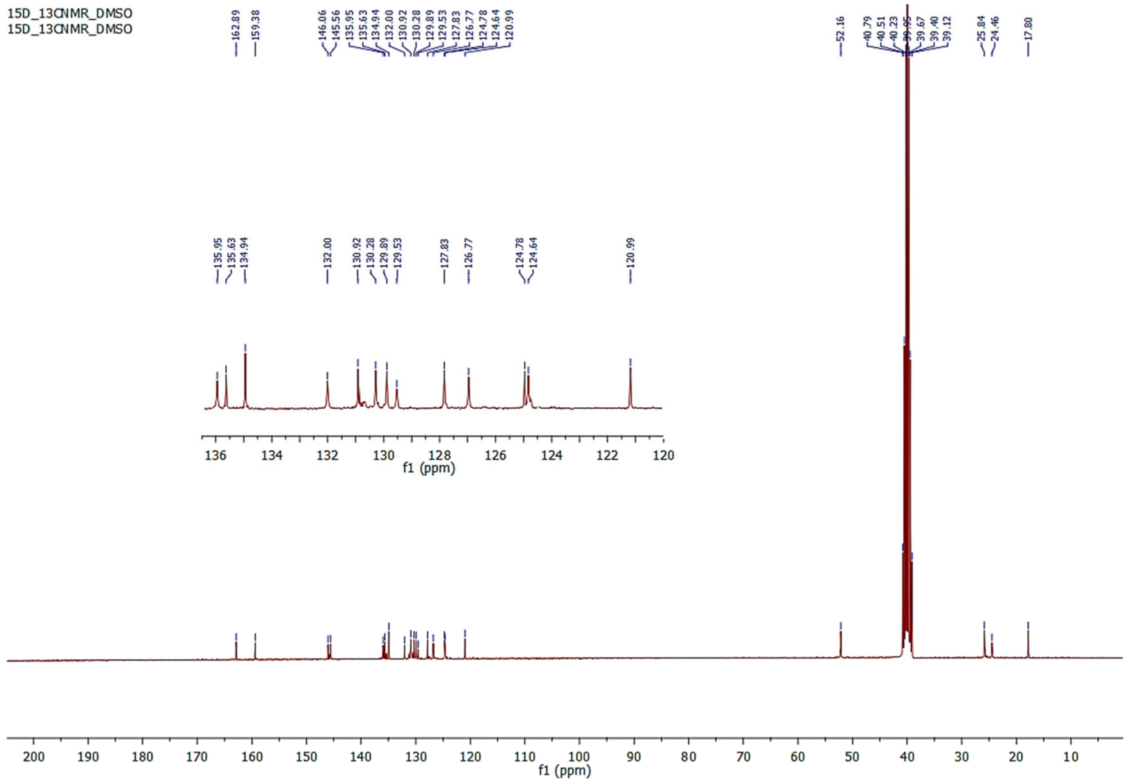

Spectra of 9e

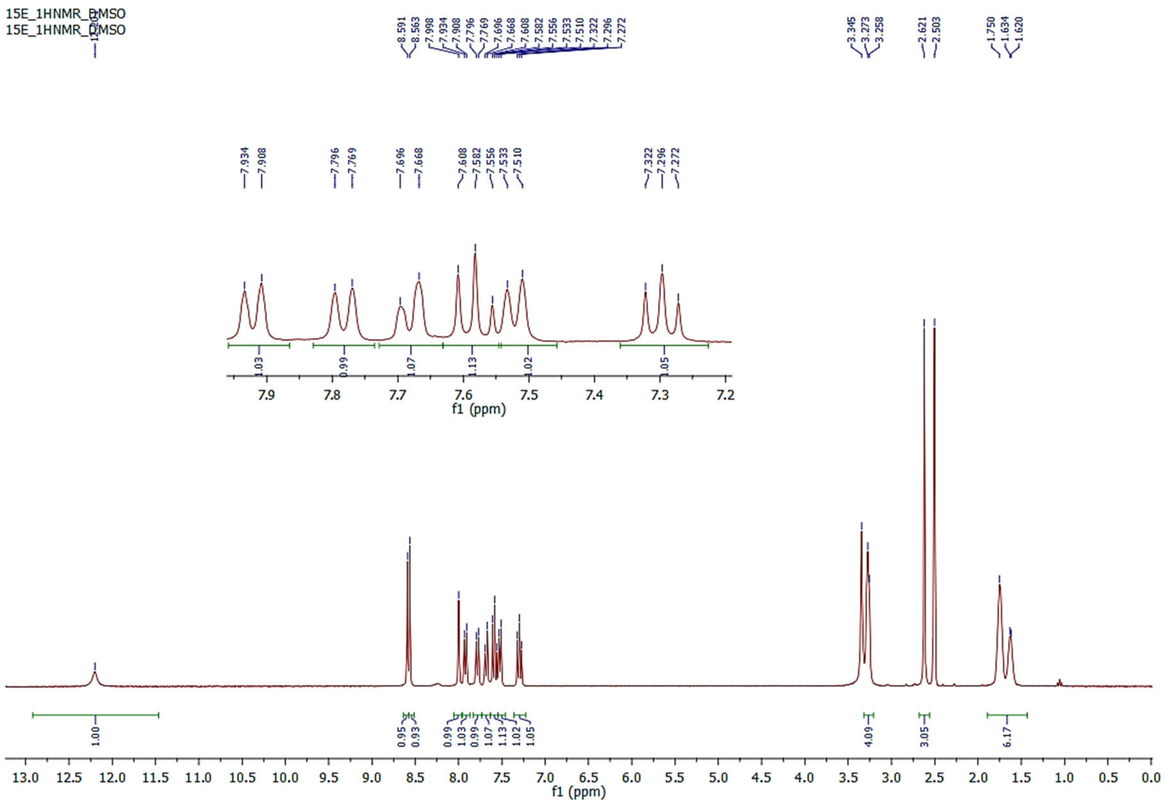

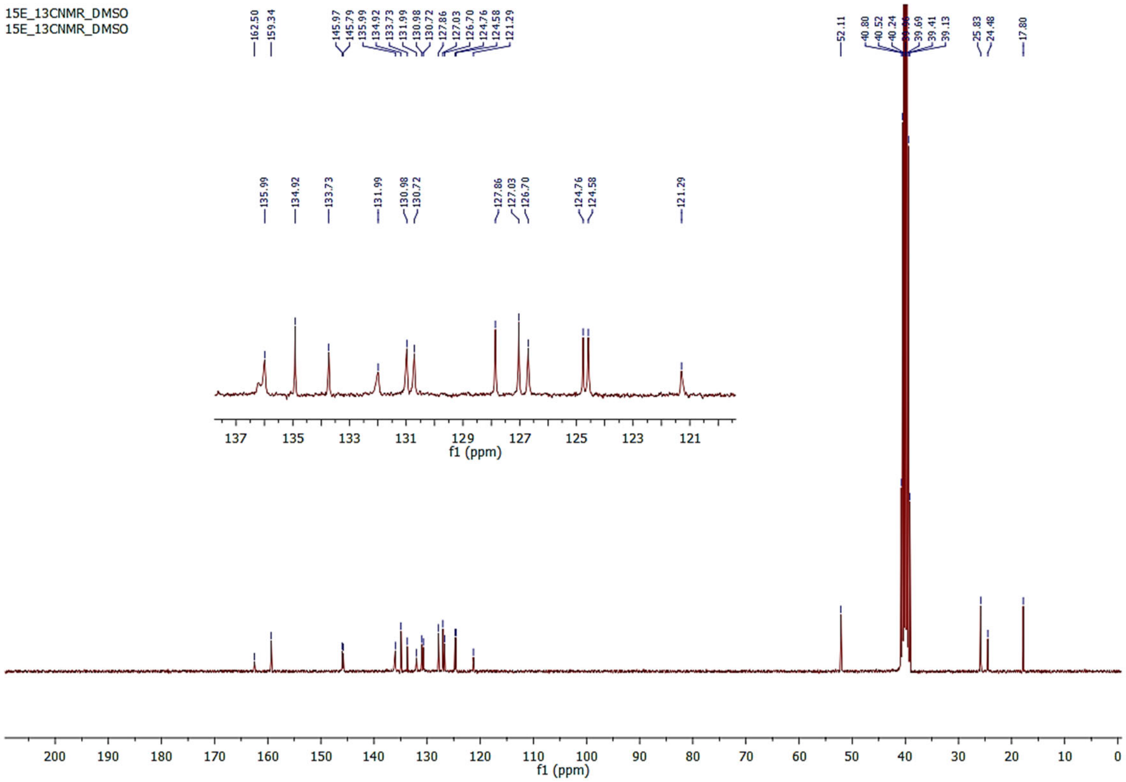

Spectra of 9f

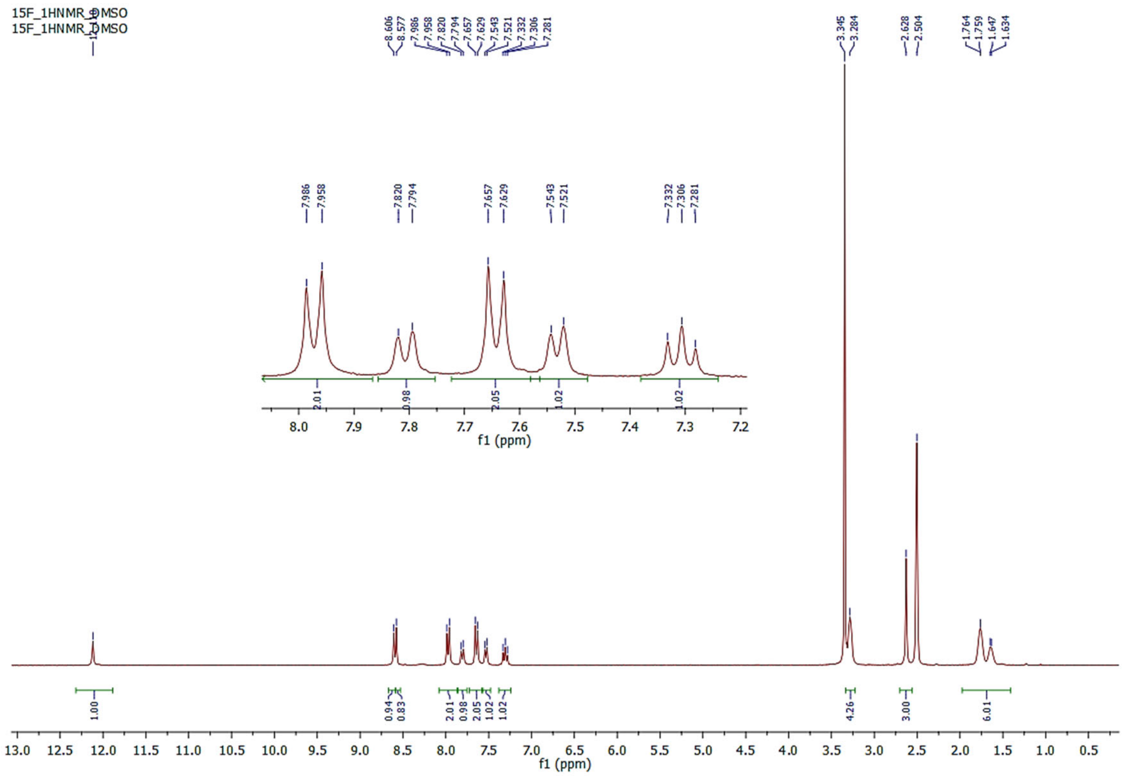

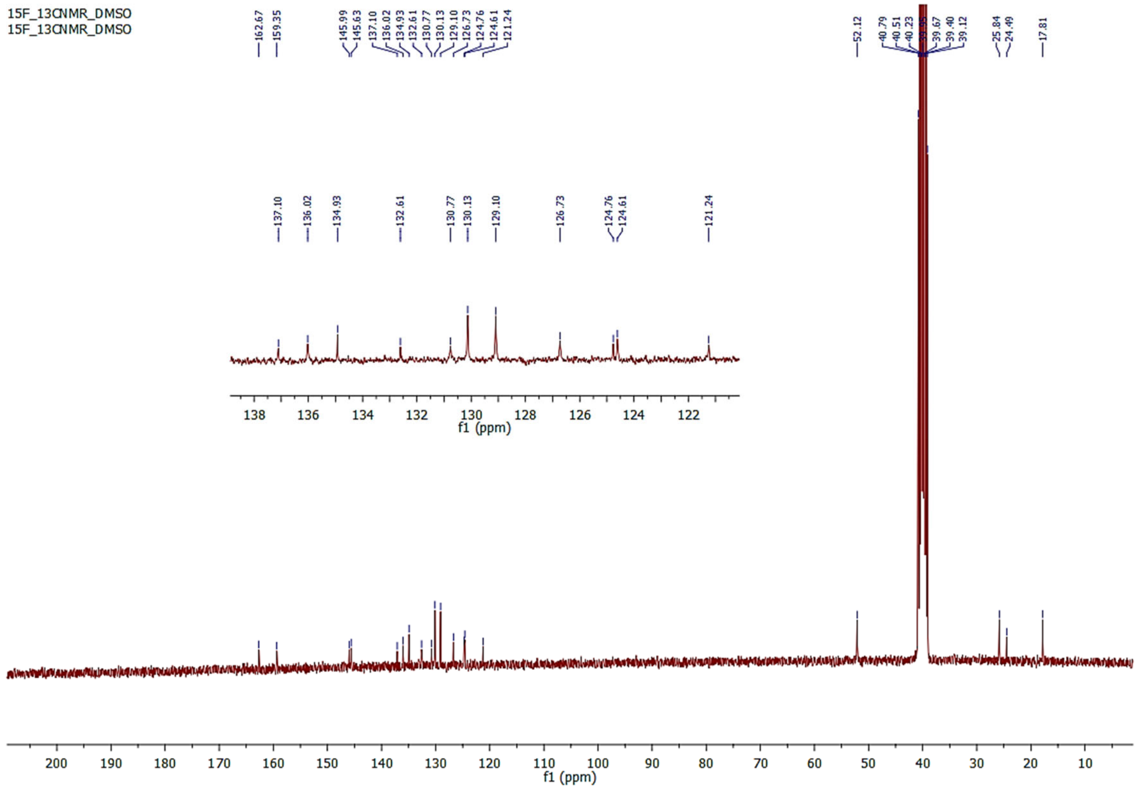

Spectra of 9g

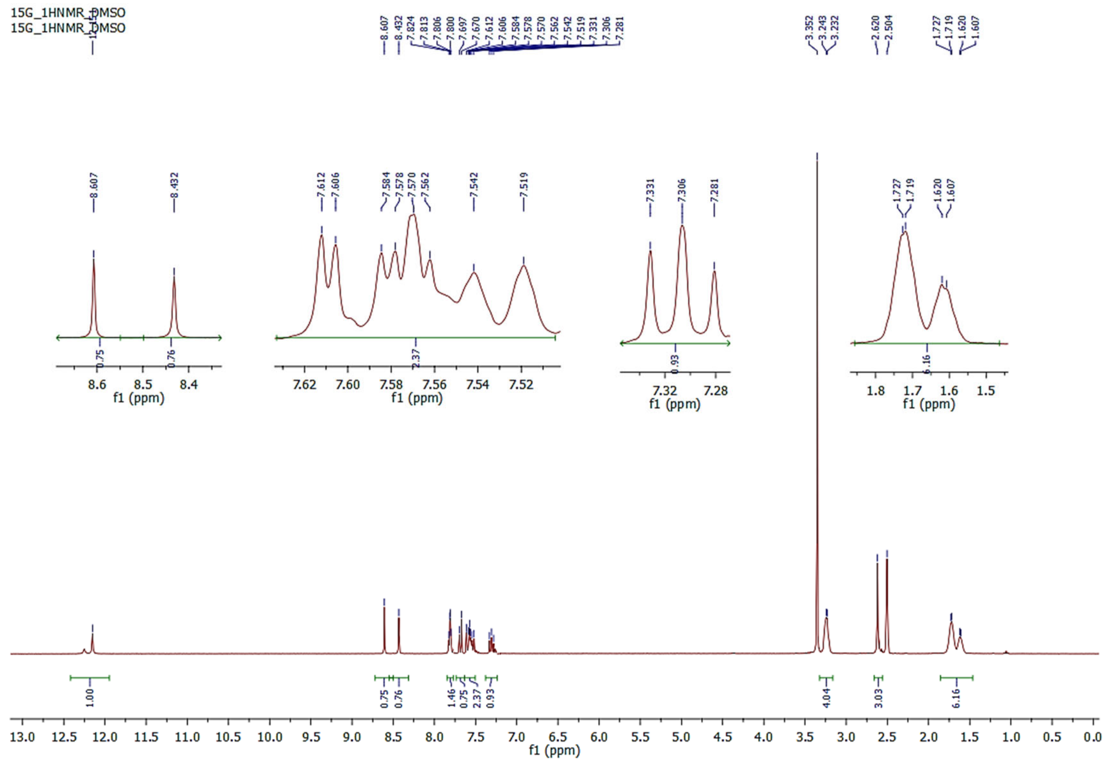

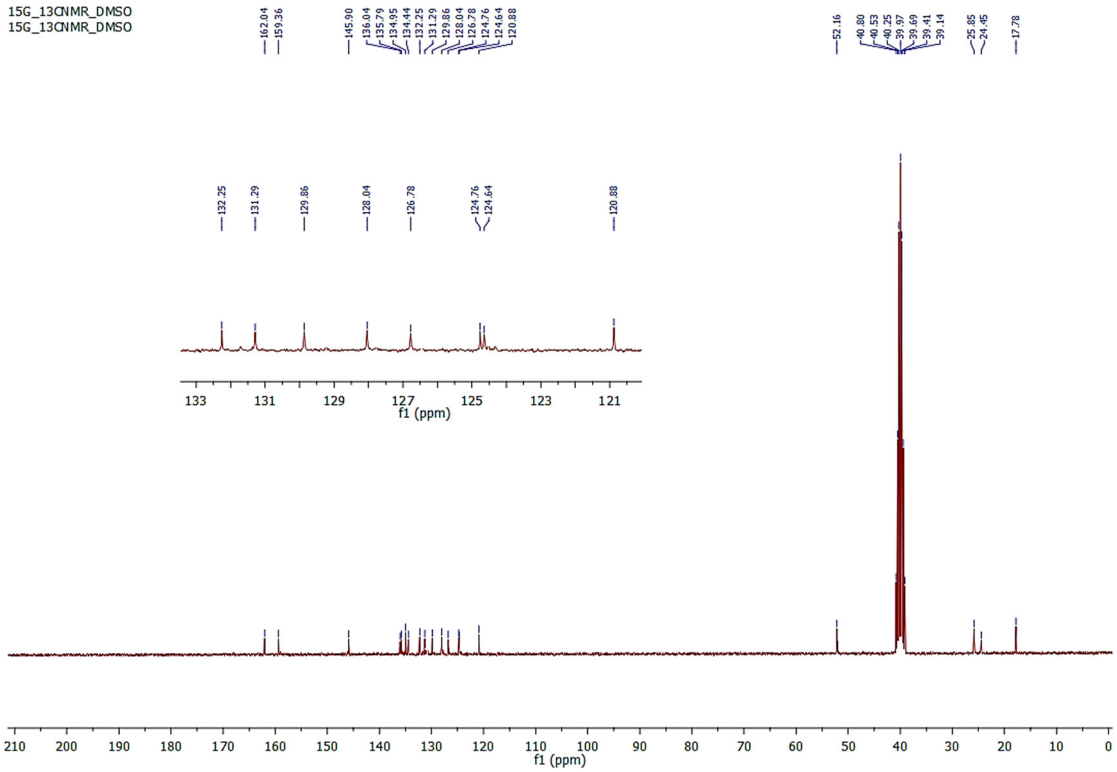

Spectra of 9h

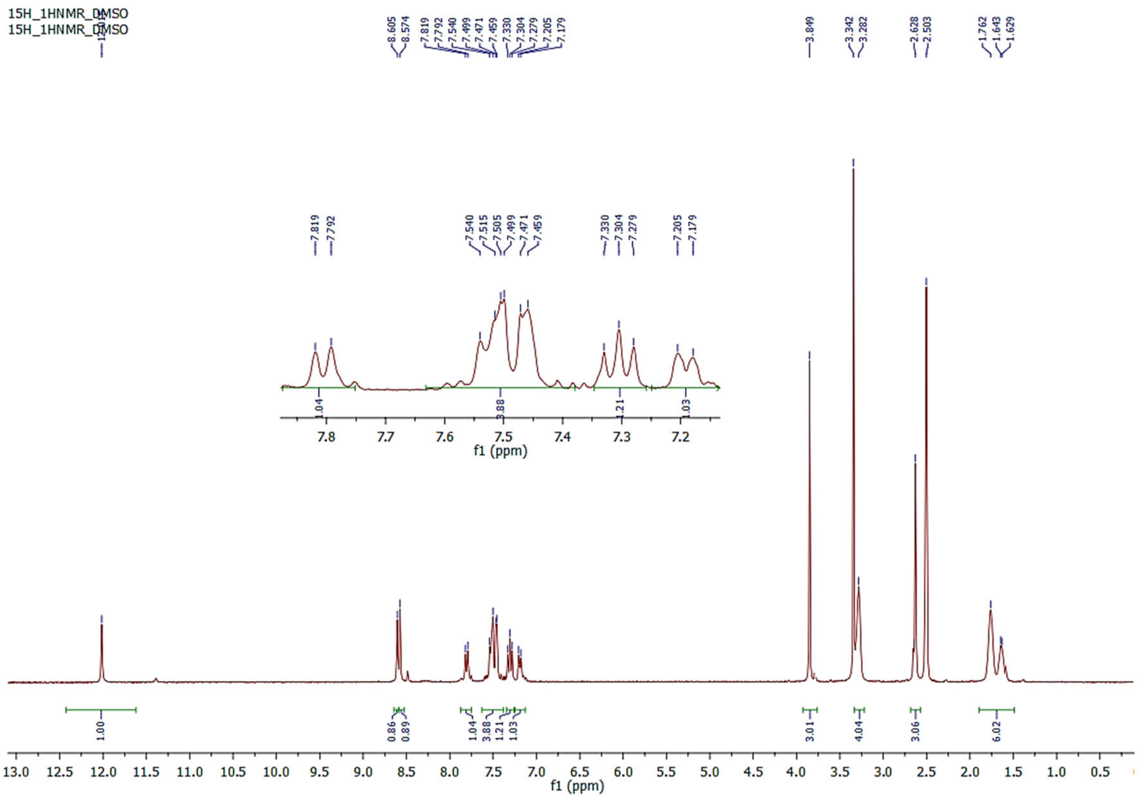

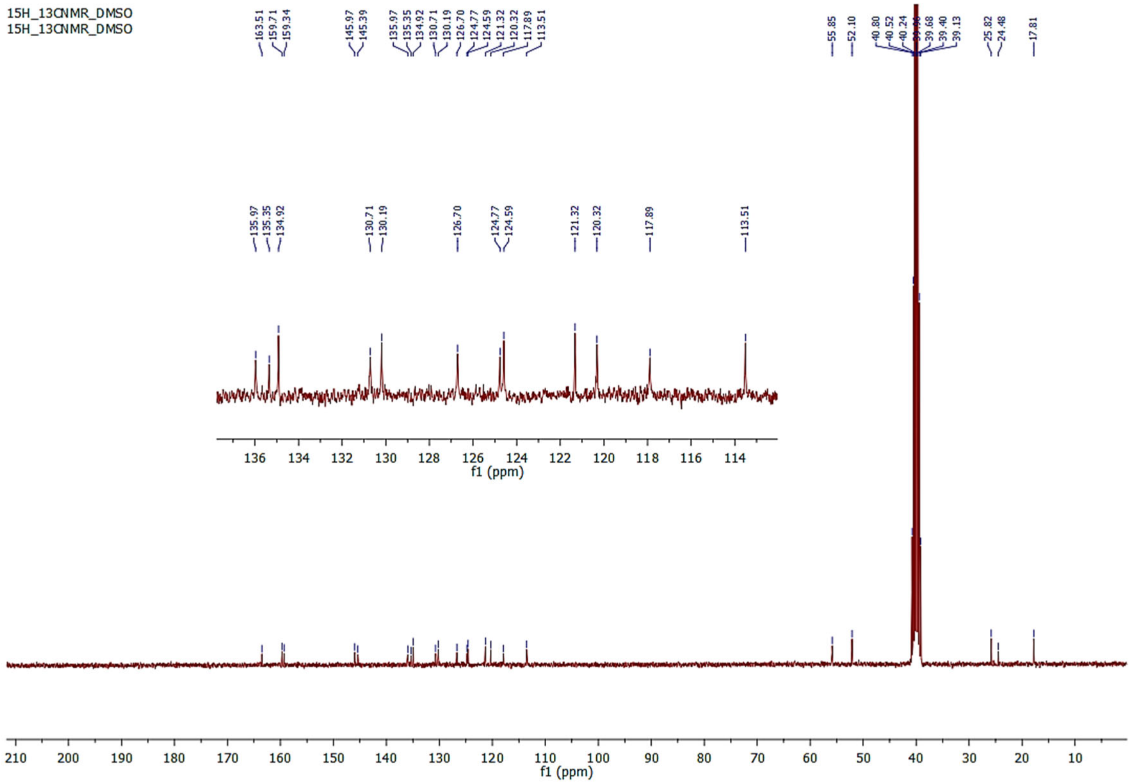

Spectra of 9i

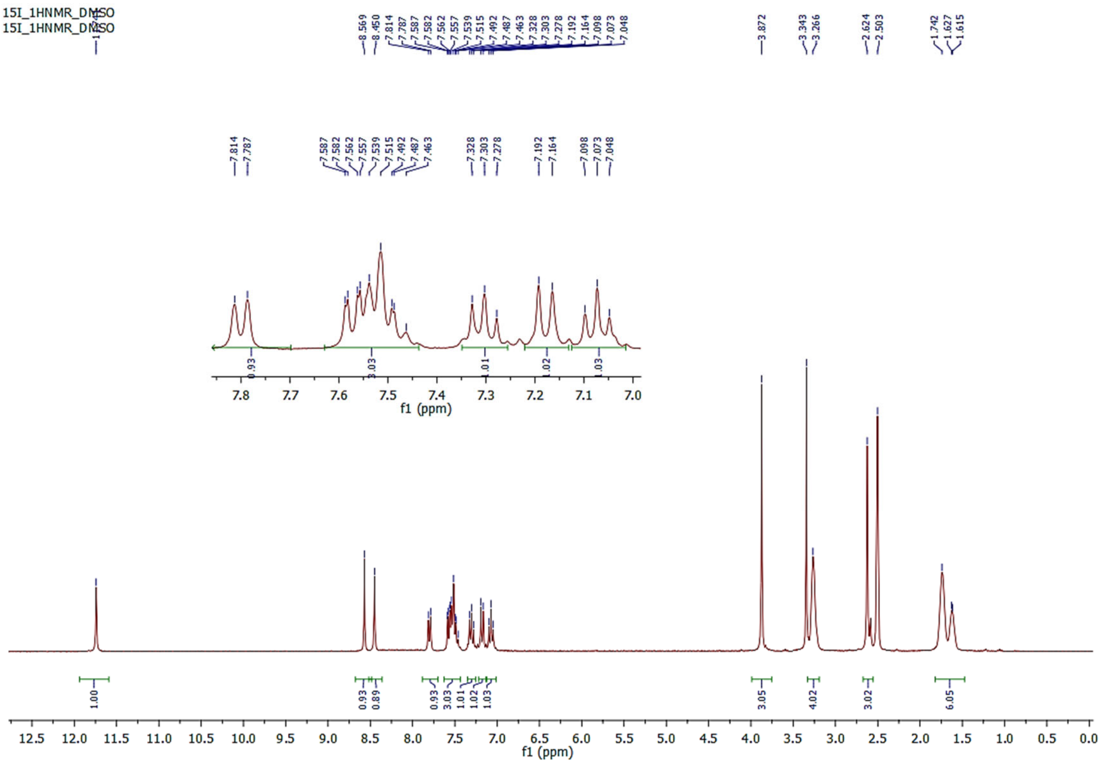

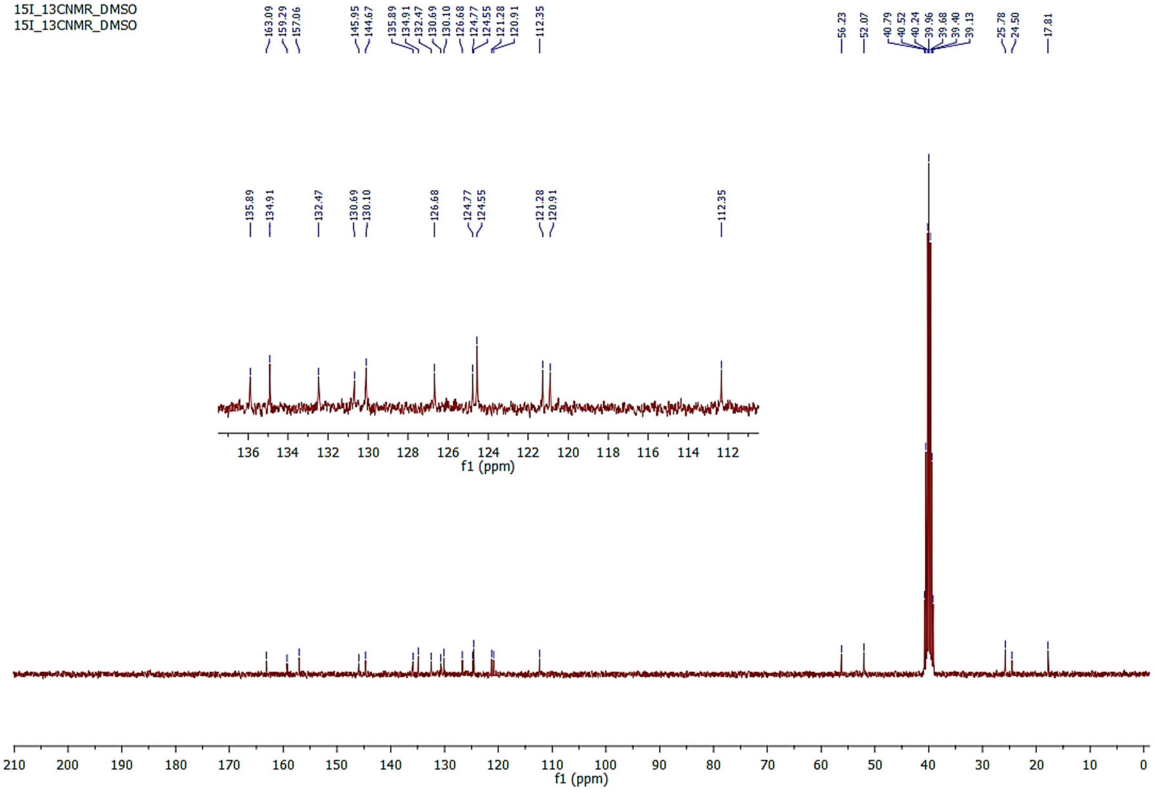

Spectra of 9j

SUPPLEMENTARY INFORMATION

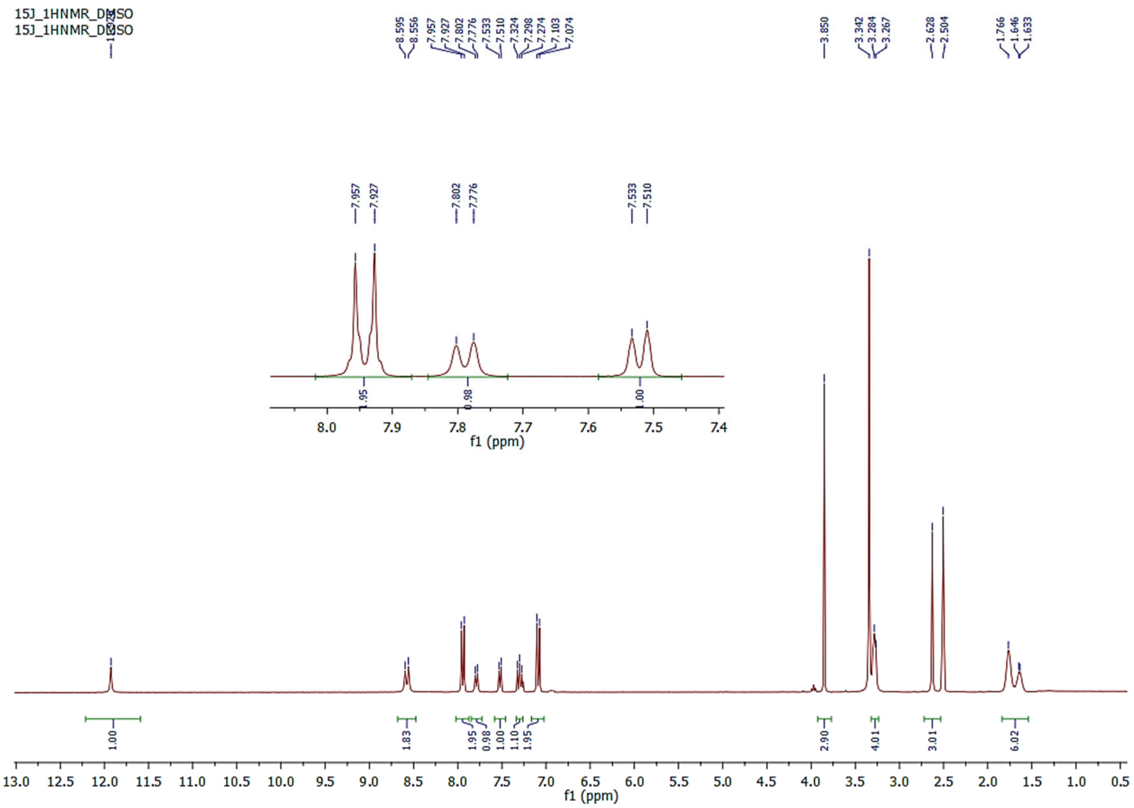

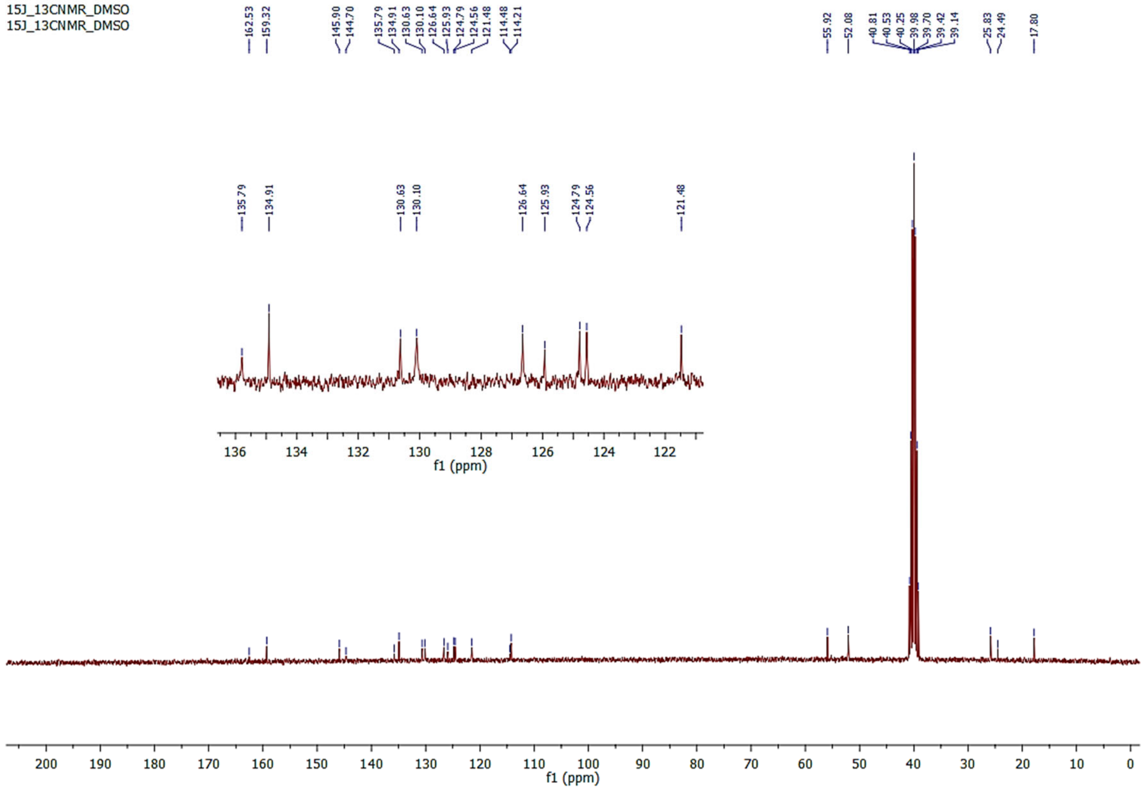

Supplement: Supplementary file 1 [file molecules-28-02131-s001.zip › molecules-2232554-supplementary.pdf]
